# Current understanding of eryptosis: mechanisms, physiological functions, role in disease, pharmacological applications, and nomenclature recommendations

**DOI:** 10.1038/s41419-025-07784-w

**Published:** 2025-07-01

**Authors:** Anton Tkachenko, Mohammad A. Alfhili, Jawaher Alsughayyir, Alessandro Attanzio, Abdulla Al Mamun Bhuyan, Bożena Bukowska, Antonio Cilla, Martha A. Quintanar-Escorza, Michael Föller, Ondrej Havranek, Kashif Jilani, Anatolii Onishchenko, Etheresia Pretorius, Volodymyr Prokopiuk, Ignazio Restivo, Luisa Tesoriere, Grazia Maria Virzì, Thomas Wieder

**Affiliations:** 1 Department of Cryobiochemistry, Institute for Problems of Cryobiology and Cryomedicine of the National Academy of Sciences of Ukraine, Kharkiv, Ukraine; 2 BIOCEV, First Faculty of Medicine, Charles University, Vestec, Czech Republic; 3 Chair of Medical and Molecular Genetics Research, Department of Clinical Laboratory Sciences, College of Applied Medical Sciences, King Saud University, Riyadh, Saudi Arabia; 4 Department of Clinical Laboratory Sciences, College of Applied Medical Sciences, King Saud University, Riyadh, Saudi Arabia; 5 Department of Biological, Chemical and Pharmaceutical Sciences and Technologies, University of Palermo, Palermo, Italy; 6 Department of Veterinary and Animal Sciences, University of Rajshahi-6205, Rajshahi, Bangladesh; 7 Department of Biophysics of Environmental Pollution, Faculty of Biology and Environmental Protection, University of Lodz, Lodz, Poland; 8 Nutrition and Food Science Area, Faculty of Pharmacy and Food Sciences, University of Valencia, Burjassot, Spain; 9 Medicine and Nutrition Faculty, Universidad Juárez del Estado de Durango, Durango, Dgo México; 10 Department of Physiology, University of Hohenheim, Stuttgart, Germany; 11 First Department of Internal Medicine-Hematology, General University Hospital and First Faculty of Medicine, Charles University, Prague, Czech Republic; 12 Department of Biochemistry, University of Agriculture, Faisalabad, Pakistan; 13 Department of Physiological Sciences, Faculty of Science, Stellenbosch University, Stellenbosch, South Africa; 14 Department of Biochemistry and Systems Biology, Institute of Systems, Molecular and Integrative Biology, Faculty of Health and Life Sciences, University of Liverpool, Liverpool, UK; 15 Research Institute of Experimental and Clinical Medicine, Kharkiv National Medical University, Kharkiv, Ukraine; 16 Department of Nephrology, Dialysis and Transplant, St Bortolo Hospital, Vicenza, Italy; 17 IRRIV - International Renal Research Institute Vicenza, Vicenza, Italy; 18 Institute of Physiology I, Eberhard Karls University Tübingen, Tübingen, Germany

**Keywords:** Apoptosis, Mechanisms of disease

## Abstract

Early studies have shown that erythrocytes have caspase-3 and caspase-8 and are capable of dying through an apoptotic-like cell death triggered by Ca^2+^ ionophores. This cell death is associated with apoptosis-like morphological signs, including cell shrinkage, membrane blebbing, and phosphatidylserine externalization. To emphasize that mature erythrocytes don’t have the apoptotic mitochondrial machinery and distinguish this unique cell death modality from apoptosis, it was named “eryptosis”. Over recent decades, our knowledge of eryptosis has been significantly expanded, providing more insights into the uniqueness of cell death pathways in erythrocytes. In this review, we aim to summarize our current understanding of eryptosis, formulate the nomenclature and guidelines to interpret results of eryptosis studies, provide a synopsis of morphological and biochemical features of eryptosis, and highlight the role of eryptosis in health and disease, including its druggability.

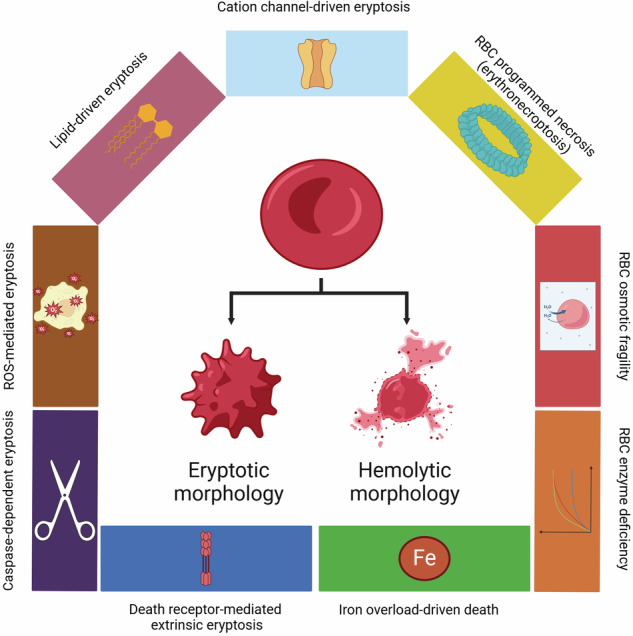

## FACTS

- Mature RBCs are capable of adjusting their cell fate based on incoming life/death signals, undergoing distinct regulated cell death modalities: eryptosis and necroptosis.
- Eryptosis is a type of regulated cell death of mature erythrocytes critically dependent on Ca^2+^ signaling and linked with cell shrinkage, membrane blebbing, and phosphatidylserine externalization.
- Eryptosis aims to shorten the lifespan of damaged erythrocytes and to facilitate their removal from circulation.

## Introduction

Cell death is one of the fundamental characteristics and principles of all eukaryotic cells. Physiologically occurring cell death, referred to as programmed cell death (PCD), is essential for appropriate embryonic development of multicellular organisms as well as for tissue remodeling during adulthood [1]. PCD represents a special category of regulated cell death (RCD), which is a more broadly defined category as a cell demise occurring in an orderly fashion through a well-coordinated genetically encoded machinery in response to a wide spectrum of internal and external perturbations [2]. When a cell faces irretrievable distress that is too strong to be adjusted for and to maintain homeostasis, it frequently activates specific signaling cascades leading to a particular cell death type [3]. Based on a collaboration between leading scientists in the field of cell death, the Nomenclature Committee on Cell Death (NCCD) summarized their consensus on RCD within an article presented in *Cell Death Differentiation*. It defined individual distinct RCD modalities, including intrinsic and extrinsic apoptosis, autophagy-dependent cell death, entotic cell death, ferroptosis, lysosome-dependent cell death, mitochondrial permeability transition-driven necrosis, mitotic death, necroptosis, NETotic cell death, parthanatos, and pyroptosis [4]. Recent advances in the field of RCD enabled the inclusion of additional newly described RCDs such as alkaliptosis [5], cuproptosis [6], disulfidptosis [7], oxeiptosis [8], or PANoptosis [9].

Classification of RCDs is driven by the underlying molecular machinery and related molecular events linked to a particular RCD. Even though different RCDs are considered individual and independent cell death modes for classification purposes, interconnectivity and extensive crosstalk on the basis of the shared molecular signaling network, redundancy, feedback, and backup function are well documented [10]. The extensive intracellular network of cell death-regulating factors implies an elaborate and refined multi-level regulation of cell death signaling. It occurs at the level of gene transcription [11, 12], epigenetic regulation [13–15], post-translational modifications of core and accessory cell death machinery components [16–18], organelle-specific mechanisms [19], and organelle crosstalk [20, 21]. It is important to mention that a core cell death machinery is evolutionarily conserved and is omnipresent in the majority of cellular types in multicellular organisms. However, it was controversial whether mature erythrocytes, terminally differentiated organelle-lacking cells, can undergo RCD. Early studies have demonstrated that erythrocytes possess caspase-3 and caspase-8 and can die via an apoptotic-like cell death triggered by Ca^2+^ ionophores. This process is associated with morphological alterations typical for apoptosis, like cell shrinkage, membrane blebbing, and phosphatidylserine (PS) translocation to the outer leaflet of the cell membrane phospholipid bilayer [22, 23]. Ca^2+^-driven cell death of erythrocytes can occur even without activation of caspases. As erythrocytes lack crucial apoptotic mitochondrial and nuclear machinery, and in order to distinguish this cell death mode of erythrocytes from apoptosis, it was named “eryptosis” [24]. Consequently, multiple studies expanded our understanding of eryptosis' underlying molecular machinery, triggers, inhibitors, and physiological significance. In this review, we aim to summarize our current understanding of eryptosis, formulate a nomenclature and guidelines to interpret results of eryptosis studies, provide a synopsis of morphological and biochemical features of eryptosis, and highlight the role of eryptosis in health and disease, including its druggability.

Of note, the NCCD suggests avoiding the term “eryptosis” due to the complexity and disputability of the life/death status of mature erythrocytes, notwithstanding the emphasis on its physiological and pathophysiological importance [4]. However, a discovery that bacterial pore-forming toxins can induce necroptosis (a programmed necrosis) also in red blood cells (RBCs) is striking and could eventually lead to a re-conception of erythrocytes as entities between life and death [25]. In nucleated cells, apoptosis and necroptosis don’t occur simultaneously, with each inhibiting the other one [26]. There is some evidence that such mutual exclusivity is present also in erythrocytes, complementing the situation in nucleated cells [27, 28]. New recent evidence might, therefore, indicate that erythrocytes are not just mechanistically disintegrated entities but might be capable of adjusting their cell fate based on incoming life/death signals.

## Erythrocytes: structural features (lack of organelles) determine the cell death signaling pathways

Unlike normal nucleated cells, mature erythrocytes are hemoglobin-filled, flexible, thin nucleus- and organelle-free entities of biconcave shape with a size of 7–8 µm [29]. Their shape and flexibility have been perfectly designed to provide highly efficient gas exchange, the main physiological function of erythrocytes.

In adults, mature erythrocytes develop as a result of erythropoiesis from bone marrow-located hematopoietic stem cells through a gradual and consequent process that includes stepwise formation of myeloid progenitors, megakaryocytic-erythroid progenitors, burst-forming unit-erythroids (BFU-e) and colony-forming unit-erythroids (CFU-e), proerythroblasts, basophilic, polychromatic, and orthochromatic erythroblasts, reticulocytes, and eventually mature erythrocytes [30, 31]. Enucleation and clearance of organelles are crucial events of erythropoiesis, which occur at the stage of erythroblasts and/or reticulocytes. The elimination of organelles evolved to provide more space for oxygen-carrying hemoglobin and to maintain the biconcave shape of RBCs [32]. Enucleation, a rate-limiting step of erythrocyte maturation, is tightly regulated via coordination of multiple transcription factors (FOXO3, E2F2, etc.), miRNAs (miR-30a, miR-191, miR-181a), cytoskeleton proteins (F-actin, dynein, tropomodulin, etc.), and kinases (p38 MAPK, p38 mitogen-activated protein kinase) [33]. Mitochondrial clearance occurs via macroautophagy or mitophagy [34, 35]. Furthermore, it has been reported that lysosomes, peroxisomes, Golgi apparatus, endoplasmic reticulum (ER), and ribosomes are also cleared from the erythroid lineage cells in an autophagy-dependent fashion [32, 36].

It is important to note that organelle-containing erythroid lineage cells can undergo intrinsic [37] and extrinsic apoptosis [38], receptor-interacting serine/threonine-protein kinase 1 (RIPK1)-dependent necroptosis [39], or iron-driven ferroptosis [40]. However, a lack of organelles, especially mitochondria, limits the cell death machinery of mature erythrocytes and their ability to undergo specific, regulated cell death modes. Certain retained components of various cell death pathways such as Fas (CD95)/Fas ligand (FasL, CD95L) signaling, caspase-3, caspase-8, or RIPK1/RIPK3 (receptor-interacting serine/threonine-protein kinase 3)/MLKL (mixed lineage kinase domain like pseudokinase) axis can be considered a heritage of the erythroid lineage cells. Importantly, despite the existing evidence for low-level protein translation in erythrocytes [41], protein synthesis in mature erythrocytes is negligible or virtually absent, and erythrocytes cannot regulate cell death by production of cell death-associated proteins. Therefore, mature erythrocytes can regulate cell death pathways only by adjusting the activity of proteins retained during their maturation.

## Erythrocytes: canonical and non-canonical functions

Oxygen (O_2_) is an indispensable gas required in humans for multiple reactions and primarily used as the terminal electron acceptor in the mitochondrial electron transport chain at the level of complex IV to generate the proton electrochemical gradient necessary for adenosine triphosphate (ATP) synthesis by oxidative phosphorylation [42]. Thus, survival and normal functionality of cells are critically dependent on oxygen delivery performed by the heme-containing hemoglobin protein, which is the most abundant protein in erythrocytes [43]. Hemoglobin, as a gas-transporting molecule, is also responsible for the transport of approximately 23% of carbon dioxide (CO_2_), a final product of metabolism, from tissues to lungs for elimination from the body [44]. The main part of the CO_2_ is transported as HCO^-^_3_ in the plasma. However, erythrocytes are also a functional part of this transport mechanism, as they contain the enzyme carbonic anhydrase and the most abundant erythrocyte membrane protein, anion exchanger 1 (AE1, Band 3 protein, SLC4A1). In the tissues, CO_2_ enters the erythrocyte by diffusion, where it is hydrated with the help of carbonic anhydrase. Then, HCO_3_^-^ is formed by deprotonation and transported to the blood plasma by AE1 (in exchange with Cl^-^). In the capillaries of the lung, these reactions run in the reverse direction, and the gaseous CO_2_ leaves the body via the alveoli of the lung.

Importantly, recent research progress has substantially broadened our understanding of the additional physiological functions of erythrocytes. In addition to the canonical role in gas exchange or maintenance of acid-base balance, accumulating evidence indicates that erythrocytes are involved in the regulation of blood flow, hemostasis, redox equilibrium, and immune response.

Erythrocytes play a crucial role in the regulation of eNOS-derived nitric oxide (NO) bioavailability, acting as scavengers of this critical vasodilator with an effect on vascular tone and blood flow [45]. In addition to NO scavenging by oxyhemoglobin, further studies have demonstrated that erythrocytes can also store and release NO in hypoxic conditions [46]. Thus, the context-dependent regulation of NO bioavailability by erythrocytes could fine-tune blood flow.

Abundant experimental evidence suggests a critical contribution of erythrocytes to blood clotting regulation. Erythrocytes affect hemostasis and thrombosis by altering blood viscosity, blood flow dynamics, secreting RBC-derived microvesicles, interactions with platelets or endothelial cells, fibrinogen-induced RBC aggregation, activation of blood coagulation factors, direct modulation of thrombin and fibrin formation, or formation of polyhedral-like erythrocytes in thrombi [47–49]. An abundance of erythrocytes affects the rheological properties of blood, exponentially increasing the viscosity [49]. The extensive crosstalk between erythrocytes and platelets or endothelial cells has been shown to regulate thrombosis. For instance, export of ATP and adenosine diphosphate (ADP) by erythrocytes in shear stress conditions stimulates platelet activation and aggregation [49, 50]. In pathological conditions, erythrocytes can adhere to endothelial cells, which promotes the formation of microaggregates with consequent blood flow reduction [50]. Externalized PS induces thrombin generation via factor X activation. Given the abundance of RBCs in blood, erythrocytes have been reported to produce up to 40% of thrombin in certain pathological conditions [47, 49, 50]. Moreover, microvesicles generated by RBCs contain PS molecules, which mediate their prothrombotic effects. Erythrocyte-derived microvesicles have been shown to induce blood clotting through activation of Hageman factor (factor XII) [51]. Additionally, protein post-translational glycosylation-mediated association between the levels of von Willebrand factor (vWF) and ABO blood groups has been established [52]. Polyhedrocytes formed from erythrocytes (up to 90% of RBCs in thrombi) have been demonstrated to have an impact on the outcome of thrombosis, decreasing the embologenicity of blood clots [48].

Despite the inability of erythrocytes to upregulate antioxidant enzymes at the transcriptional level, its antioxidant system, relying primarily on reduced glutathione (GSH), is involved in the regulation of the redox equilibrium in blood. This is achieved by the ability of erythrocytes to scavenge and detoxify exogenous neutrophils-, monocytes- or endothelial cells-derived reactive oxygen species (ROS) and reactive nitrogen species (RNS) [53, 54].

Erythrocytes have been increasingly recognized to be immunologically active. A pioneering study of Lam et al. showed expression of toll-like receptor 9 (TLR9) in intact erythrocytes, mediating the capability to bind bacterial and plasmodial cell-free DNA, as well as synthetic CpG DNA [55]. TLR9-mediated effects in mature erythrocytes include activation of erythrophagocytosis and enhancement of the innate immune response [55, 56]. Additionally, erythrocytes can scavenge chemokines via Duffy antigen receptor for chemokines (DARC), leading to changes in their availability and consequent regulation of immune cell migration [57]. Erythrocytes have also been reported to bind pathogens [58]. Extracellular erythrocyte-derived Hb is known as a potent source of ROS, which has bactericidal effects [59].

A canonical role of hemoglobin as a gas carrier was identified and explained over a century ago. However, as research continued, erythrocytes were shown to affect vascular tone, immunity, redox homeostasis, and blood clotting.

## Cell death in erythrocytes: ACD (hemolysis) and RCD (eryptosis and necroptosis) modalities

**Fig. 1:**
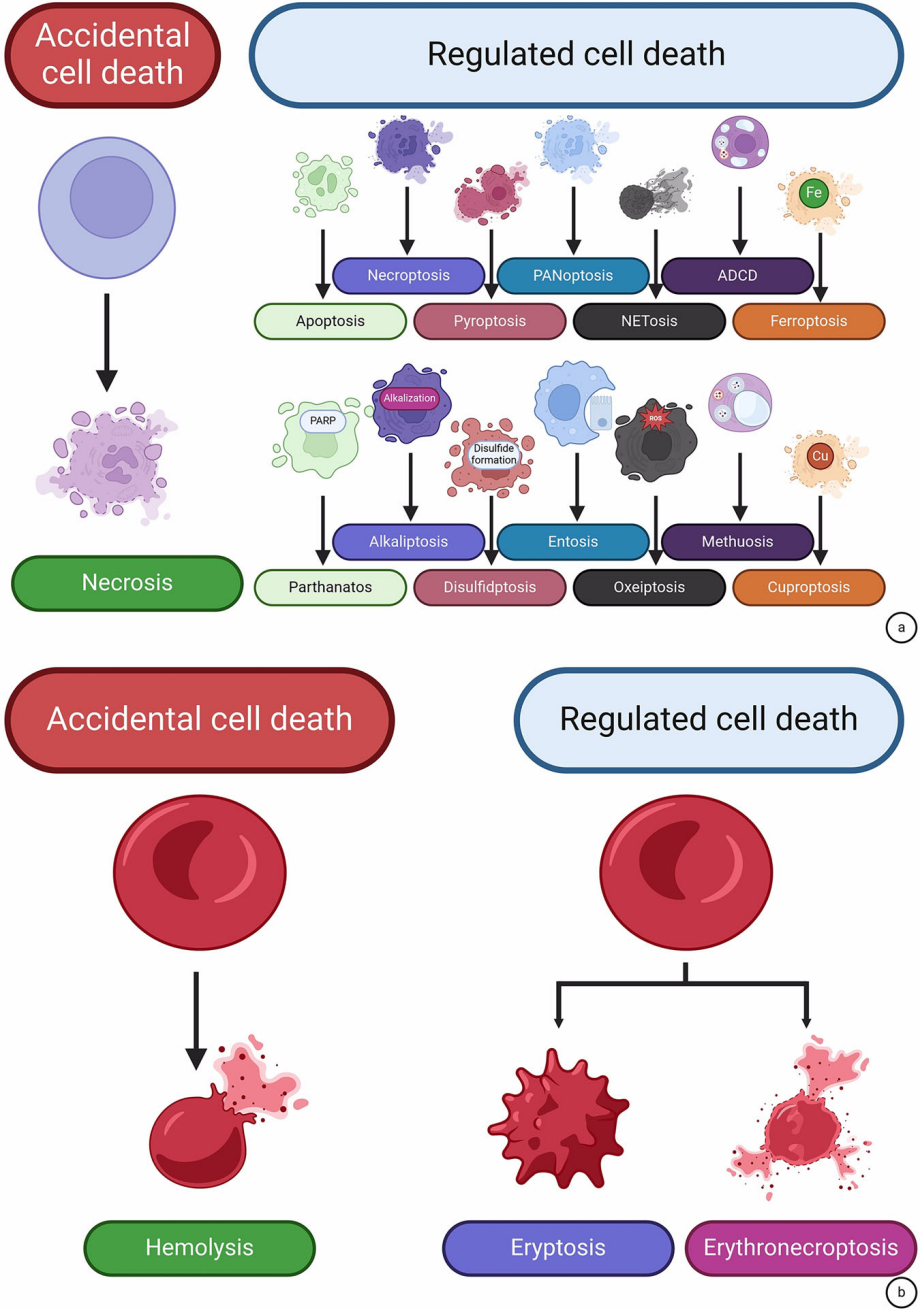
The set of cell death modalities is scarce is mature erythrocytes. Accidental and regulated cell death modalities in nucleated cells (**a**) and mature erythrocytes (**b**). Lack of organelles in mature erythrocytes restricts the diversity of the cell death machinery. ACDC autophagy-dependent cell death, PARP poly (ADP-ribose) polymerase, ROS reactive oxygen species. Created with Biorender.com.

Death of eukaryotic cells is fundamentally classified into accidental cell death (ACD) and RCD. ACD occurs in response to environmental perturbations, i.e., any mechanical insult compromising the membrane integrity. It develops at once and does not require any machinery to occur. Lack of signaling pathways that can modulate ACD occurrence suggests that ACD cannot be targeted pharmaceutically [4]. Uncontrollable necrosis has been considered an ACD [60]. On the other hand, RCD requires the genetically encoded machinery, which is frequently quite complex and sophisticated. Furthermore, RCD develops gradually, which allows a cell to launch an adaptive response, aiming at avoiding it. This indicates that RCD is preventable and hence it can be modulated by therapeutic agents [4]. RCD pathways are extensively studied. They form a tightly regulated signaling network with multiple overlaps in signaling pathways between distinct RCDs to ensure the most appropriate cellular response to stress factors [1]. However, apoptosis, necroptosis, ferroptosis, pyroptosis, cuproptosis, PANoptosis, NETosis, autophagy-dependent cell death, mitotic death, mithuosis, alkaliptosis, anoikis, autosis, disulfidptosis, entosis, oxeiptosis, and parthanatos are reported as distinct RCD modes with unique signal transduction pathways (Fig. 1a). We suggest that this approach in the classification of cell death can be applied to erythrocytes as well. Hemolysis, which is associated with the rupture (lysis) of erythrocytes, is an ACD of mature erythrocytes [61]. Osmotic fragility of erythrocytes is a parameter that at least in part regulates hemolysis under hypo-osmotic conditions. It has recently been described that enhanced band 3-aquaporin 1 interaction supports osmotic resistance, thereby reducing the tendency of hypo-osmotic burst and erythrocyte ACD.

Like in necrosis, hemolytic erythrocytes lose the integrity of their cell membranes, resulting in the release of their content. Some of the released molecules, referred to as damage-associated molecular patterns (DAMPs) like heme and its derivatives, ATP, heat shock protein (HSP70), or interleukin-33 (IL-33), are capable of triggering the immune response, amplifying inflammation [62, 63].

Due to a lack of organelles, erythrocytes have long been considered membrane-surrounded sacks carrying hemoglobin [64]. However, over two decades ago, mature erythrocytes were first shown to contain functional caspase-3 and caspase-8, which fueled research on the cell death of erythrocytes [22, 23]. It is important to note that cells in the erythroid lineage can undergo a fully-fledged apoptosis [38] and other RCDs reported for nucleated cells, like necroptosis [65] and ferroptosis [40]. However, maturation of erythrocytes and conversion of erythroblasts to mature erythrocytes during erythropoiesis is accompanied by enucleation and organelle clearance [32]. This results in the loss of an appreciable part of the cell death machinery, which determines the uniqueness of cell death pathways in mature erythrocytes. At the moment, only two RCD modalities with clearly described and distinct molecularly defined pathways have been reported for mature erythrocytes, termed eryptosis and erythronecroptosis: counterparts of apoptosis and necroptosis (Fig. 1b).

## RCD subroutines in erythrocytes: are they limited to just eryptosis and necroptosis?

The lifespan of erythrocytes is approximately 120 days. Aging of erythrocytes is associated with a wide array of structural, biochemical, and functional alterations, which facilitate clearance of senescent erythrocytes by phagocytic cells. Senescent erythrocytes are characterized by loss of surface area and volume [66], increased cell density [67], reduced deformability [68], cation loss [69], membrane desialylation [70], enzymatic dysregulation [69], oxidative stress [71], hemichrome abundance [69], elevated levels of glycated hemoglobin [72], reduced expression of phagocytosis-inhibiting CD47 [73], altered protein 4.1a/protein 4.1b ratio [74], etc. Notably, aging-related AE1 protein conformational changes result in the appearance of a senescent-specific antigen recognized by autologous antibodies [75]. Despite some reports on the similarities between senescence and eryptosis at the morphological level [67], intracellular Ca^2+^ elevation [74], Ca^2+^-driven activation of Gardos channels [70], PS exposure [76], or ROS contribution [71], senescence of erythrocytes cannot be considered an RCD of these cells and should be clearly distinguished from eryptosis. We suggest that the clearly demonstrated differences in clearance rates of senescent (cleared within days) and eryptotic cells (eliminated within minutes) emphasize that these two mechanisms for erythrocyte clearance are distinct [72, 77, 78]. It has been demonstrated that aged erythrocytes are especially prone to eryptosis associated with oxidation-induced PS exposure [72]. Thus, eryptosis can occur in compromised aged erythrocytes to further speed up their clearance. In this context, PS externalization indicates activation of eryptosis in senescent erythrocytes, suggesting that senescent erythrocytes exposing PS should be considered as eryptotic cells.

Erythrocytes don’t have the intrinsic apoptotic machinery upstream of caspase-3, including cytochrome c or apoptotic peptidase-activating factor 1 (APAF-1) [22, 23]. Notably, cell death of erythrocytes cannot be triggered by staurosporine [23], which is a potent apoptosis-inducing agent, but it is triggered by the Ca^2+^ ionophore ionomycin [79]. Unlike nucleated cells, erythrocytes are unable to store Ca^2+^ intracellularly. Thus, Ca^2+^ influx through Ca^2+^ permeable cation channels is crucial to mediate eryptosis in RBCs, which might be referred to as cation channel-driven eryptosis. Eryptosis might be ignited and propagated by ROS (ROS-mediated eryptosis), ceramide (lipid-driven eryptosis), Fas signaling (extrinsic eryptosis), and be associated with activation of caspases (caspase-dependent eryptosis). As erythrocytes from patients with inherited enzyme deficiencies (e.g., glucose-6-phosphate dehydrogenase-deficient cells) are more susceptible to different inducers of eryptosis, it seems reasonable to mention RBC enzyme deficiency-dependent erythrocyte death [80].

Another breakthrough in investigating the cell death machinery of erythrocytes occurred when expression of necroptosis-associated RIPK1, RIPK3, and MLKL was confirmed in mature erythrocytes, and they were shown to undergo regulated necrosis [25, 27, 81, 82]. This regulated necrotic cell death is activated in response to Fas/FasL signaling, CD59 ligation combined with pore formation, mediated by ROS, ceramide, and advanced glycation end products (AGEs) [28]. There is still scarce data about the signaling pathways and especially the physiological role of erythrocyte necroptosis. However, the shared signaling pathways suggest that there is an extensive crosstalk between eryptosis and RBC necroptosis. Lack of organelles and death receptors such as TNR-R1 (CD120a), TNR-R2 (CD120b), TRAIL-R1 (DR4), TRAIL-R2 (DR5) in mature erythrocytes, which are known to be involved in necroptosis induction, determine the presence of erythrocyte-specific features of erythrocyte necroptosis highlighted in a recently published review, which resulted in the emergence of the term “erythronecroptosis” to describe RIPK1/RIPK3/MLKL-dependent lytic cell death of mature erythrocytes [28].

Mature erythrocytes are unable to regulate protein content at the level of expression, since they are devoid of the nucleus and protein-synthesizing apparatus. Thus, to execute cell death, erythrocytes can rely only on the retained components of the cell death machinery inherited from the erythroid precursors. Although its functionality is not compromised, this imposes a certain level of “simplification” of cell death pathways in mature erythrocytes as compared with the nucleated cells. Moreover, an abundance of iron and susceptibility to oxidative stress have resulted in the hypothesis that erythrocytes might undergo ferroptosis, an iron-driven ROS-dependent RCD [78]. The occurrence of ferroptosis in mature erythrocytes has not been confirmed and it seems quite challenging, since ferroptosis studies suffer from the lack of well-established markers and evaluation of this cell death requires a complex approach with identification of typical cellular and subcellular morphological alterations, genetic hallmarks, biochemical changes, expression of certain ferroptosis-related proteins, etc. Furthermore, ferroptosis is tightly regulated at the epigenetic, transcriptional, and post-translational levels, which can hardly be achieved in mature erythrocytes. However, iron overload in patients with hemochromatosis results in excessive PS externalization and calpain activation in RBCs [83]. Thus, it is suggested to apply the term “iron overload-driven cell death.”

## Morphological signs of eryptosis

Eryptosis is characterized by a wide array of morphological alterations, which generally coincide with those observed in apoptotic cells [84]. It has been demonstrated that eryptosis might alter cell volume, shape, granularity of erythrocytes, as well as modify the phospholipid composition of both inner and outer leaflets of phospholipid bilayers in the cell membrane, leading primarily to PS externalization [85, 86]. Notably, cell shrinkage and membrane blebbing are the most characteristic morphological signs of apoptotic cells [87–89]. Our progress in understanding the molecular mechanisms governing eryptosis allowed shedding light on signaling pathways mediating the development of morphological alterations associated with eryptosis. For instance, abundant evidence supports that cell shrinkage is mediated by K^+^ efflux. Ca^2+^ overload, which is a crucial event in eryptosis, leads to the opening of Ca^2+^-activated K^+^ channel KCa3.1, known as the Gardos channel [90]. Erythrocytes lose K^+^ ions through the Gardos channels, which culminates in a sharp decrease in cell volume. Notably, the contribution of the Gardos channels to eryptosis-associated cell shrinkage is supported by the fact that their inhibitors charybdotoxin and clotrimazole can avert cell shrinkage [78, 79]. Moreover, intracellular Ca^2+^ elevation indirectly contributes to membrane blebbing. Ca^2+^ overload activates calpain, a cysteine protease that degrades the cytoskeleton [91]. This calpain-mediated rearrangement of the erythrocyte cytoskeleton results in membrane blebbing [88, 89]. It is important to note that AE1plays an important role in the maintenance of the structural integrity of erythrocytes, providing interactions between the membrane and the cytoskeleton, supporting the cytoskeletal connectivity, and maintaining the biconcave shape of erythrocytes [92]. Experimental evidence suggests that caspase-3 can proteolytically degrade the N-terminal cytoplasmic domain of AE1 [93, 94]. Moreover, Band 3 protein can also be degraded by calpain [95].

However, the eryptosis-associated changes in cell volume are not limited only to shrinkage. In particular, there is some evidence that eryptotic cells might undergo swelling, which is much less common [89]. Furthermore, spiculations appear in eryptotic cells, resulting in the loss of their biconcave shape [85]. Of note, Jacob et al. developed a software-based technique, which allowed performing morphometric analysis of eryptotic cells evaluating cell size (cell shrinkage), cell irregularities (membrane blebbing), granularity, and central halo (loss of a biconcave shape) [85].

Thus, it can be summarized that morphological alterations typical of eryptosis, including cell shrinkage and membrane microvesiculation, are governed by the elevation of intracellular calcium ion levels.

## Signaling in eryptosis

**Fig. 2:**
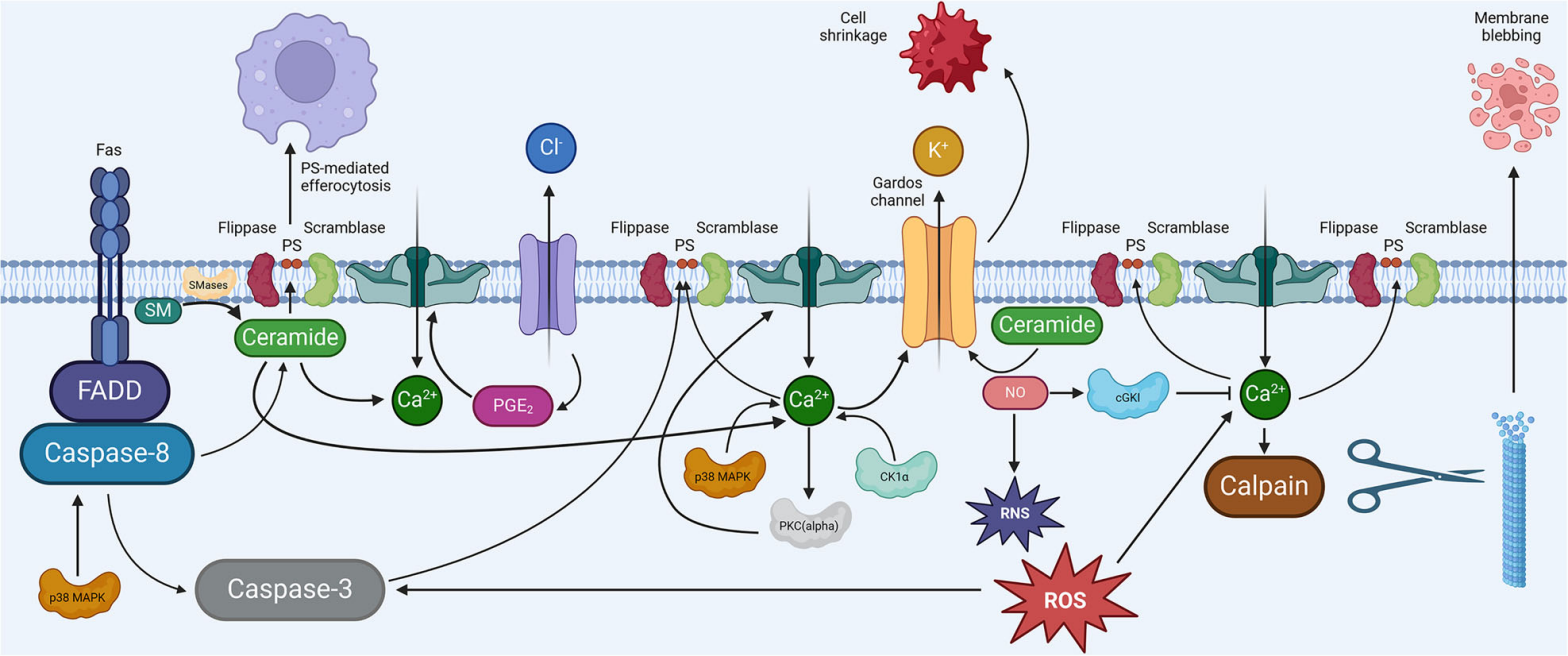
Eryptosis is a calcium-mediated, regulated cell death of erythrocytes associated with cell shrinkage, membrane microvesiculation, and phosphatidylserine exposure, resulting in efferocytosis, the phosphatidylserine-mediated clearance of eryptotic erythrocytes by macrophages. cGKI cGMP-dependent protein kinase I, CK1α casein kinase 1α, FADD Fas-associated death domain, p38 MAPK p38 mitogen-activated protein kinase, PGE_2_ prostaglandin E2, PKC protein kinase C, PS phosphatidylserine, RNS reactive nitrogen species, ROS reactive oxygen species, SM acid and neutral sphingomyelin, SMases acid and neutral sphingomyelinases. Created with Biorender.com.

The main pathways of eryptosis signaling are summarized in Fig. 2 and described in detail in the “Ca^2+^ signaling,” “ROS signaling,” “Ceramide signaling,” “Eryptosis and caspases,” “Crosstalk between eryptosis and necroptosis in erythrocytes: Is caspase-8 involved?,” “Eryptosis-associated kinases,” “Extrinsic Fas-mediated eryptosis,” “Nitric oxide signaling,” “GTPase signaling,” and “Cellular energy metabolism and eryptosis” sections

### Ca^2+^ signaling

Ca^2+^ plays an important role in apoptosis signaling of nucleated cells [96]. Especially, Ca^2+^ fluxes involving the ER and mitochondria govern apoptosis [96]. In contrast, Ca^2+^ entry from the extracellular space may be of minor importance [97]. Although erythrocytes do not have ER or mitochondria, Ca^2+^ is also of high relevance to eryptosis [90]. In fact, an increase in the intracellular Ca^2+^ concentration is a major trigger of eryptosis (Fig. 2), and many factors stimulating eryptosis do so by inducing this elevation [90]. In theory, an increase in the cytosolic Ca^2+^ concentration of an erythrocyte may be the consequence of Ca^2+^ influx from the extracellular space, decreased Ca^2+^ transport from the cytosol to the extracellular space, or release from intracellular Ca^2+^ binders.

Similar to nucleated cells, the intracellular Ca^2+^ concentration of erythrocytes is kept in the low nM range [74], whereas the extracellular Ca^2+^ is orders of magnitude larger (low mM range). This steep Ca^2+^ gradient creates a strong driving force for Ca^2+^ entry and is maintained by the low Ca^2+^ permeability of the resting erythrocyte and high Ca^2+^-extruding capacity through a Ca^2+^ ATPase and Na^+^/Ca^2+^ exchanger in the cell membrane.

The requirement of Ca^2+^-permeable channels in the erythrocyte membrane for eryptosis was postulated early, although their molecular identity has remained unknown for long [79]. Their characterization by electrophysiological approaches revealed that they are mostly closed under basal conditions and activated by different stimuli that are also known to induce eryptosis: ROS, exposure to high extracellular osmolarity, or removal of extracellular Cl^-^ and isosmotic replacement with gluconate activate the channel [98] and trigger eryptosis [90].

The molecular identification of these channels has been subject to intense research. Members of the transient receptor potential C (TRPC) family of cation channels are Ca^2+^-permeable and were suggested to be involved [90]. In progenitor cells of the erythrocytic line, TRPC3 mediates Ca^2+^ influx [99]. TRPC6 is also expressed in these erythrocytic progenitor cells [99], and in humans, erythrocytes TRPC6 accomplishes Ca^2+^ influx [100] in contrast to mouse erythrocytes, where TRPC4/5 are involved [101].

Apart from TRPC channels, a role for ionotropic glutamate receptors for Ca^2+^ entry has been suggested: NMDA receptors permeable to Ca^2+^ accomplish Ca^2+^ homeostasis [102, 103], and also AMPA receptors may be relevant as pharmacological AMPA receptor antagonism blunts Ca^2+^ entry into erythrocytes in response to Cl^-^ removal [104]. Mechanical stress induces Ca^2+^ influx through the mechanosensitive channel PIEZO1 [105]. Also, voltage-dependent Cav2.1 channel is expressed in the erythrocyte membrane, and pharmacological evidence suggests its contribution to erythrocyte Ca^2+^ entry [106]. However, whereas it appears to be established that the aforementioned channels provide Ca^2+^ entry into erythrocytes, it still remains unclear whether and to what extent these channels actually contribute to the Ca^2+^ entry leading to eryptosis.

Erythrocyte shrinkage and PS exposure are apoptotic events that are clearly linked to an elevation of intraerythrocytic Ca^2+^ levels [90]. Erythrocytes express Ca^2+^-activated K^+^ channels (KCa3.1 encoded by *KCNN4*), also called Gardos channels [107]. In response to an increase in the cytosolic Ca^2+^ concentration, the Gardos channel opens, resulting in the efflux of K^+^ with subsequent hyperpolarization driving Cl^-^ exit (through Cl^-^ channels). Water follows the electrolytes, leading to cell shrinkage, a hallmark of eryptosis [90]. Also, PS exposure, the other hallmark of eryptosis, is Ca^2+^-dependent [90]. Flippase is an enzyme active under basal conditions that shifts PS from the outer to the inner membrane leaflet, thereby preventing PS exposure. Elevated intracellular Ca^2+^ inhibits flippase and activates scramblase, moving PS to the erythrocyte surface [108, 109]. Moreover, erythrocytes express calpain-1 [110], a Ca^2+^-dependent protease that may contribute to the breakdown of the cytoskeleton during eryptosis. However, the lifespan of erythrocytes from calpain-1-deficient mice is unaltered [110].

### ROS signaling

Evolution of life in an oxygen (O_2_)-containing atmosphere inevitably brought along the appearance of O_2_-derived cellular metabolites known as ROS. ROS encompass various molecules, the most important ones being superoxide anions (^.^O_2_^-^), hydroxyl radicals (^.^OH), and hydrogen peroxide (H_2_O_2_). It is generally accepted that the production of oxidants has to be strictly regulated, and that the ability of cells and organisms to adequately respond to oxidative stress is one of the main regulators of aging and lifespan [111]. Mitochondria and peroxisomes are the major endogenous sources of ROS. In addition, the oxidants may also originate from cytosolic enzyme systems, e.g., NADPH oxidases, and from exogenous triggers such as ionizing radiation and chemotherapeutics. To maintain homeostasis, organisms and cells developed a sophisticated enzymatic and non-enzymatic antioxidant defense system including catalase (CAT), superoxide dismutase (SOD) and glutathione peroxidase (GPx) that counteracts excessive ROS levels. Importantly, ROS does not only play the role of the bad and ugly. Instead, these reactive molecules are urgently needed for physiological processes. One example is the oxidative burst of neutrophils, which is a necessary part of normal immune function. Likewise, a balanced ROS production and ROS-dependent signaling seem to be favorable for normal growth behavior and cellular differentiation. In line with this, a precision redox paradigm was introduced for the use of antioxidant pharmacology [112]. The authors propose here the new “5R” principle, which considers the “**R**ight species, **R**ight place, **R**ight time, **R**ight level, and **R**ight target.” Erythrocytes are organelle-free cells that do not possess mitochondria and peroxisomes. Nevertheless, they are hemoglobin- and thus iron-filled containers specialized to transport O_2_ [78]. Erythrocytes are therefore exposed to high levels of oxidative stress and at the same time possess an efficient antioxidant defense system, mainly glucose-6-phosphate dehydrogenase (G6PD) and 6-phosphogluconate dehydrogenase, yielding NADPH. NADPH, in turn, is a cofactor of glutathione reductase (GR), converting glutathione disulfide (GSSG) into its reduced state (GSH). GSH is then needed for detoxification and survival of the erythrocytes [113]. Tilting the redox balance towards oxidative stress in nucleated cells, i.e., by increasing the ROS levels, normally leads to cell death (apoptosis) or to an acceleration in ageing [111]. Although long neglected, it is now widely accepted that organelle-free erythrocytes can undergo a special form of RCD (eryptosis), and the differences and similarities of apoptosis and eryptosis have been reviewed in a recent article [84]. In this line, the authors of one of the first studies on programmed erythrocyte death demonstrated that erythrocytes from patients with different forms of anemia (sickle cell anemia, thalassemia, and G6PDdeficiency) are more susceptible toward oxidative stress as compared with erythrocytes from healthy volunteers. After a challenge with *tert*-butyl hydroperoxide, erythrocytes showed signs of cell death, such as PS exposure and cellular shrinkage [80]. In the meantime, ROS-mediated eryptosis (Fig. 2) is widely accepted, and several inducers of this type of erythrocyte death have been described, including the antiinflammatory compounds Bay 11-7082, parthenolide, and dimethyl fumarate [113], H_2_O_2_ [114], the food additive E407a (semi-refined carrageenan) [115], and the tricyclic antidepressant desipramine [116]. Interestingly, other data showed that erythrocyte death is closely linked to erythrocyte aging. In this study, the susceptibility to eryptosis increases with the age of the erythrocytes, a phenomenon that is partially due to enhanced sensitivity to oxidative stress [72]. To verify the hypothesis that ROS stress leads to eryptosis, antioxidants were used to protect RBCs from premature death. Indeed, it was clearly shown that the antioxidant N-acetyl-L-cysteine (NAC) can inhibit eryptosis in vitro [116] and significantly prolong the half-life of circulating mouse erythrocytes in vivo [72] presumably by interfering with efferocytosis of eryptotic cells. Interestingly, nanoparticle (NP)-induced cell death has been described recently as being consistent with the new “5 R” principle. It was shown that ultraviolet light-activated NPs give rise to high ROS levels in leukocytes without inducing ROS in erythrocytes and eryptosis in vitro [117]. In summary, high ROS levels lead to irreversible damage to erythrocyte biomolecules, mainly lipids or proteins, thereby inducing eryptosis. In the long run, these ROS-mediated effects might influence the rheological properties of erythrocytes, thereby demonstrating the clinical relevance of eryptosis research [118]. Thus, we suggest the term ROS-mediated eryptosis when O_2_-derived reactive metabolites are involved.

### Ceramide signaling

Sphingolipids are structural components of most eukaryotic cellular membranes and can also be found in the plasma membrane of organelle-free erythrocytes. Besides their special organization in lipid domains (lipid rafts) of the membrane lipid bilayers, some sphingolipids, e.g., ceramides, sphingosine, and sphingosine-1-phosphate have been described to function as lipid-derived second messengers mainly regulating fundamental cellular pathways such as apoptosis and differentiation [119, 120]. Ceramide itself can either be generated by hydrolysis of sphingomyelin in the plasma membrane or by de novo synthesis from the amino acid serine and two fatty acids [121]. As de novo synthesis of ceramide is located in the ER, which is absent in erythrocytes, it is thus assumed that erythrocytic ceramide should directly originate from the hydrolysis of sphingomyelin. Indeed, in the first report that demonstrated a significant role of ceramide in erythrocyte death pathways, it has been ruled out that the elevated ceramide levels originate from the biosynthetic pathway [122]. Instead, the authors showed that hyperosmotic shock leads to breakdown of sphingomyelin, accumulation of ceramide, and later on to PS exposure and cell shrinkage. A few years later, it was demonstrated that sphingosine but not sphingosine-1-phosphate induces eryptosis [123]. This makes perfect sense as erythrocytes take up large amounts of sphingosine and detoxify this sphingolipid by enzymatic conversion to sphingosine-1-phosphate by sphingosine kinase [124]. Thus, lipid-driven eryptosis is mainly attributed to ceramide generation in the plasma membrane (Fig. 2). Triggers of elevated ceramide levels comprise hyperosmotic shock [122], secreted sphingomyelinases (SMases) during inflammation [125], sepsis [126] or severe coronavirus disease 2019 (COVID-19) [127], *Clostridium perfringens* epsilon toxin [128], and platelet-activating factor [129]. In principle, sphingomyelin can be hydrolyzed by different enzymes: neutral SMase, acidic SMase, and secreted forms of SMases [120]. In the context of erythrocyte death, several enzymatic activities have been described to be involved in sphingomyelin breakdown: (i) cigarette smoke extracts (CSEs) elicit neutral SMase activity-dependent erythrocytic ceramide formation [130]; (ii) Cu^2+^ induces the secretion of acidic SMase from leukocytes which in turn leads to accumulation of erythrocytic ceramide [131] and (iii) treatment of erythrocytes with extracellular SMase resulted in ceramide-associated alterations in membrane-cytoskeleton interactions and membrane organization [125]. To identify the downstream targets of ceramide in nucleated cells, a ceramide signaling network was generated by mining the PubMed literature database. According to this, several proteins were listed, incl. the apoptosis-regulating proteins Bcl-2 and Bax, the protein phosphatases PP2A and PPP1C, and the cell division control protein Cdc-42 [120]. However, as human erythrocytes are nucleus- and organelle-free cells, the effects of erythrocytic ceramides should be restricted to the plasma membrane and plasma membrane-associated proteins. In this line, a consistent ceramide signaling pathway was observed in human erythrocytes; after stimulation of SMase-dependent ceramide formation, assembly of the death-inducing signaling complex (DISC) and oligomerization of Fas receptor, as well as cleaved caspase-8 and caspase-3, were detected [130]. Taken together, ceramide-mediated PS exposure and cell shrinkage are one of the best-documented signaling pathways leading to erythrocyte death and efferocytosis of eryptotic cells. We therefore suggest using the term lipid-driven eryptosis when sphingolipid-derived metabolites are involved.

### Eryptosis and caspases

The NCCD defines apoptosis (both intrinsic and extrinsic) as an RCD executed by caspase-3 [4]. Caspases comprise a family of cysteine proteases composed of 12 members in humans [132]. In nucleated cells, their functions are not limited to apoptosis, and caspases have been demonstrated to be involved in the regulation of multiple RCDs and related mechanisms, including pyroptosis (caspase-1), necroptosis (caspase-8), mitotic catastrophe (caspase-2), and regulation of inflammation [4]. Recent studies have suggested that caspases are involved in the extensive crosstalk between distinct lethal subroutines, as well as between apoptosis and inflammatory pathways [133]. Early research demonstrated that erythrocytes retained functional caspase-3 and caspase-8 [22, 23]. However, activation of caspase-3 was not essential for the execution of apoptosis-like cell death of RBCs, resulting in the hypothesis that caspases were required for appropriate erythropoiesis [23]. The key event associated with orchestration of eryptosis, which is intracellular Ca^2+^ elevation, occurs in the absence of caspase-3 activation [134]. Lack of critical involvement of caspase-3 in eryptosis has been emphasized in an extensive analysis of the caspase-3 contribution to eryptosis, which has revealed that eryptosis triggered in vitro by diverse compounds is not associated with caspase-3 recruitment in the majority of cases [84]. At the same time, caspase-3 can be activated by ROS in erythrocytes and promote PS externalization [78, 134, 135].

We believe that this limited contribution and non-essentiality of caspase-3 for the execution of eryptosis is associated with the absence of the upstream mitochondrial pathways in mature erythrocytes linked with the expulsion of this particular organelle at the terminal stages of erythropoiesis, as well as downstream effectors. However, there is compelling evidence that caspase-3 and caspase-8 are downstream effectors of the Fas cell death signaling pathway, indicating their importance for extrinsic Fas-mediated eryptosis (see the “Extrinsic Fas-mediated eryptosis” section) [130, 136].

Why are caspases retained in mature erythrocytes? Evolutionarily, the origin of RCD, in particular, apoptosis has been postulated to be associated with endosymbiosis, i.e., domestication of mitochondria [137–139]. Organelle clearance, primarily the expulsion of mitochondria in erythroblasts or reticulocytes, is accompanied by the removal of the mitochondrial apoptosis-associated machinery. Caspases in erythropoiesis play a non-apoptotic role and are imperative for cell differentiation and proper maturation [38, 140, 141]. However, the discovery of functional extrinsic death receptor-mediated eryptosis and necroptosis of erythrocytes associated with the necrosome formation opens new horizons for our understanding of the possible role of caspases in mature erythrocytes, since caspase-8 might be involved in coordinating the cell fate of mature erythrocytes.

### Crosstalk between eryptosis and necroptosis in erythrocytes: is caspase-8 involved?

More and more studies have emphasized the existence of an intricate and extensive cell death signaling network in nucleated cells. The crosstalk between distinct RCDs is well documented and can be interpreted through the lens of the cell death-governing intracellular system with multiple redundant backup pathways [20, 142]. Along with the fundamental dichotomous choice between survival and death, the second-level decision concerning a particular cell death program to follow is probably no less important [143]. To uncover the mechanisms orchestrating the network of cell death pathways, research focuses on the identification of possible switches between distinct lethal subroutines, critically influencing the selection of a particular cell death modality acting as a connecting bridge. In nucleated cells, caspase-8, which is an initiator caspase of the extrinsic apoptotic pathway [144], has been identified as a molecular switch between apoptosis, necroptosis, and pyroptosis [145–147]. Activation of caspase-8 facilitates the execution of apoptosis, simultaneously inhibiting necroptosis by preventing RIPK1 recruitment, a key regulator of necroptosis. Furthermore, when apoptosis and necroptosis fail to occur, caspase-8 can be involved in the activation of caspase-1 and inflammasome to trigger pyroptosis, a strongly proinflammatory cell death modality associated with the release of IL-1β and IL-18 [145, 146].

Caspase-8 expression in mature erythrocytes was confirmed in 2001 [22]. The contribution of caspase-8 and caspase-3, activated downstream of the Fas/Fas-associated death domain (FADD) signaling, to the promotion of PS externalization was shown in 2005 [136]. Currently, compelling evidence indicates that caspase-8 is required to mediate extrinsic Fas/FasL-driven eryptosis in mature erythrocytes [130, 148]. In 2014, LaRocca et al. first demonstrated that mature erythrocytes were capable of dying via RIPK1-dependent necroptosis like nucleated cells [25]. Of note, necroptosis of erythrocytes was associated with the formation of the RIPK1/ FADD/caspase-8 complex, and caspase-8 activation prevented the occurrence of necroptosis. Moreover, it has been recently summarized that, like apoptosis and necroptosis in nucleated cells [26], eryptosis and erythronecroptosis are mutually exclusive [28]. Thus, caspase-8 is suggested to play a key role in the selection of RCD modalities in erythrocytes.

Identification of caspase-8 as a cell fate-regulating enzyme in mature erythrocytes might have far-reaching implications for RBC biology, since the ability to regulate and select cell death modalities questions a conceptual view on erythrocytes as entities existing in a state between life and death.

### Eryptosis-associated kinases

Intracellular signaling of eryptosis has been extensively studied, and many important protein kinases implicated in the regulation of various functions of nucleated cells have been demonstrated to control eryptosis. Based on studies involving gene-targeted mice and cell culture experiments, the significance of AMP-activated protein kinase (AMPK), p21-activated kinase 2 (PAK2), cGMP-dependent protein kinase type I (cGKI), Janus kinase 3 (JAK3), mitogen and stress-activated protein kinase (MSK1/2), and phosphoinositide-dependent kinase 1 (PDK1) for eryptosis has been studied. AMPK, a serine/threonine kinase, consists of three subunits α, β, and γ and is a cellular energy sensor activated in cellular states of energy deficiency, and turns off energy-consuming processes while inducing pathways providing energy [149]. Moreover, it is implicated in the pathophysiology of metabolic, cardiovascular, and malignant diseases [149]. AMPKα-deficient mice exhibit splenomegaly due to accumulation of eryptotic RBCs and severe anemia, and their erythrocytes are prone to enhanced eryptosis [149]. AMPK inhibition augments energy deficiency-induced eryptosis [149]. The AMPK effect on eryptosis may, at least in part, be dependent on PAK2 [150]. Serine/threonine kinase cGMP-dependent kinase cGKI is another important inhibitor of eryptosis. JAK3 is a tyrosine kinase mainly mediating cytokine signaling in immune cells. Erythrocytes from JAK3-deficient mice are characterized by lower PS exposure upon glucose depletion, as are wild-type erythrocytes following pharmacological JAK3 inhibition [151]. Spleens from MSK1/2 (serine/threonine kinase)-deficient mice accumulate eryptotic erythrocytes, and their erythrocytes are characterized by enhanced eryptosis following glucose depletion or hyperosmotic shock [152]. PDK1-deficient erythrocytes exhibit a reduced cytosolic Ca^2+^ concentration and eryptosis upon exposure to cellular stressors [153].

Studies on the role of further protein kinases in eryptosis are solely based on cell culture experiments and pharmacological evidence: Activation of serine/threonine-protein kinase C (PKC) induces eryptosis (Fig. 2) with and without further stress stimuli, an effect attenuated by PKC inhibitors [154, 155]. Casein kinase 1α (CK1α) is a serine/threonine kinase that has broad functions in different tissues and organs, participating in WNT signaling or circadian rhythm. CK1 inhibitors attenuate, while CK1α activation aggravates eryptosis following glucose depletion, effects attributed to altered intracellular Ca^2+^ concentration [156]. p38 MAPK is a serine/threonine kinase that is activated by external cellular stressors, controlling cell death and differentiation. p38 MAPK inhibitors reduce hyperosmotic shock-induced eryptosis [157]. Inhibitors of cyclin-dependent kinase 4 (CDK4), a serine-/threonine kinase controlling cell survival and death, reduce stress-induced eryptosis [158].

### Extrinsic Fas-mediated eryptosis

A crucial step in the apoptosis mechanism in eukaryotic cells is the activation of caspases, a family of highly conserved cysteine proteases. These proteases exist as inactive zymogens and require proteolytic cleavage to become enzymatically active. Caspases can serve different roles in the apoptotic cascade: some act as initiators (e.g., caspase-8, caspase-9), while others function as effectors (e.g., caspase-3, -6, -7), ultimately executing apoptosis by cleaving essential cellular proteins [159]. One of the primary pathways leading to caspase activation involves the death receptor pathway, where receptors such as Fas/CD95, following stimulation by FasL, recruit adapter proteins like FADD to initiate the activation of procaspase-8 [160].

While the mechanisms of caspase-dependent apoptosis are well-characterized in nucleated cells, their role in enucleated cells like erythrocytes remains less well understood. Numerous studies have suggested that the development and differentiation of erythroid progenitor cells might be regulated through caspase-dependent pathways [161–163]. In 2001, Berg et al. demonstrated the presence and potential function of caspases in RBCs [22]. Notably, they discovered significant levels of caspase-3 and caspase-8 in mature RBCs. However, while these caspases were found to be functionally active in in vitro experiments, they were not activated by a variety of proapoptotic stimuli, indicating a unique regulation of apoptosis in RBCs.

The study that shed light on the protein machinery involved in the extrinsic cell death pathway in RBCs is that of Mandal et al. in 2005 [136]. The authors revealed that mature RBCs express key components of the extrinsic apoptosis pathway, including Fas, FasL, FADD, and caspase-8 and caspase-3 (Fig. 2). These proteins are typically involved in RCD in nucleated cells, highlighting a previously underexplored aspect of their activity in enucleated RBCs [164]. In aged RBCs, Fas was found to colocalize with specific lipid raft marker proteins such as Gα_s_ [165] and CD59 [166]. Lipid rafts are dynamic microdomains within the plasma membrane that serve as platforms for the clustering and activation of signaling molecules, facilitating the assembly of apoptotic complexes [167].

The association of Fas with these raft markers in aged RBCs suggests that lipid rafts play a crucial role in initiating and regulating the extrinsic death pathway in erythrocytes. In addition to the colocalization of Fas with raft markers, the researchers also detected the presence of FasL, FADD, and activated caspase-8, further supporting the formation of a functional DISC in aged RBCs. This complex initiates a cascade that leads to the activation of downstream executioner caspases, such as caspase-3, which ultimately drive the apoptotic process [136]. The study also documented significant functional differences between young and aged RBCs. Aged RBCs exhibited a marked decrease in aminophospholipid translocase activity, an enzyme responsible for maintaining the asymmetric distribution of phospholipids across the cell membrane. This loss of translocase function was accompanied by increased externalization of PS, a key “eat-me” signal that flags cells for recognition and phagocytosis by macrophages. Supporting the hypothesis that caspases play an essential role in RBC apoptosis, the study showed that oxidatively stressed RBCs mimicked key apoptotic events. Oxidative stress, induced by ROS, triggered the translocation of Fas into lipid rafts, the formation of a Fas-associated signaling complex, and the subsequent activation of caspases-8 and -3. Interestingly, these events were shown to be independent of calpain activity, a calcium-dependent protease also implicated in cell death processes. Instead, they were ROS-dependent, as demonstrated by the protective effects of the ROS scavenger NAC, which inhibited caspase activation. The activation of caspases in response to oxidative stress was closely linked to the observed reduction in aminophospholipid translocase activity and the increased PS externalization in aged RBCs. This suggests that the loss of membrane integrity and the externalization of PS, which are key features of apoptotic cells, are at least partially driven by caspase activity in mature RBCs. Overall, the study underscores the functional relevance of the extrinsic apoptotic pathway in RBC senescence and clearance.

In a later study, Biswas et al. [168], while investigating the mechanism leading to the development of anemia due to arsenic intoxication, found accelerated Fas-mediated eryptosis. Confocal microscopy analysis of RBCs from chronic arsenic-exposed rats gave evidence of Fas aggregation and DISC assembly in the cell membrane that culminated in caspase-8 and caspase-3 activation. Interestingly, the authors have shown an effective recovery from arsenic-induced death signaling in erythrocytes in response to treatment of the rats with atorvastatin and NAC in rats.

Similarly, in RBCs from chronic lead-exposed rats, oxidative stress and K^+^ loss were found to accelerate Fas translocation into lipid raft microdomains, inducing Fas-mediated death signaling [169]. Ceramide generation was a critical component of Fas receptor-induced eryptosis, whereas therapy with natural organosulfur compounds inhibited the apoptotic death of erythrocytes by antagonizing oxidative stress and the Gardos channel that led to suppression of ceramide-initiated Fas aggregation in lipid rafts of these erythrocytes.

Restivo et al. have more recently demonstrated Fas activation-induced eryptosis via ceramide formation in human erythrocytes exposed to CSE, as reported in the relevant paragraph [130].

### Nitric oxide signaling

NO is a potent eryptosis suppressor (Fig. 2) independent of Ca^2+^, as even ionomycin-induced eryptosis (ionomycin is a Ca^2+^ ionophore) is attenuated by NO donor nitroprusside [170]. Mice deficient in endothelial NO synthase (eNOS), the key enzyme for NO production in vessels, are characterized by faster erythrocyte clearance, possibly due to enhanced erythrocyte PS exposure and subsequent phagocytosis [170]. However, eNOS is also expressed in erythrocytes, and erythrocyte-derived NO may impact their deformability and suppress platelet activation [171, 172]. In erythrocytes, NO may react with hemoglobin, yielding S-nitrosohemoglobin [173]. Depending on the oxygenation state, NO may be released from hemoglobin [173]. NO activates soluble guanylate cyclase [174], resulting in the generation of cGMP, which in turn activates cGKI [175]. Dibutyryl-cGMP, a synthetic cGMP derivative, reduces ionomycin-stimulated eryptosis [170]. cGKI-deficient mice are characterized by enhanced eryptosis, anemia, and splenomegaly with accumulation of PS-exposing erythrocytes in the spleen [175].

### GTPase signaling

Guanosine triphosphatases (GTPases) are a group of enzymes that hydrolyze guanosine triphosphate (GTP) to guanosine diphosphate (GDP) and inorganic phosphate. GTPases can be divided into two main classes: (i) small monomeric GTPases and (ii) trimeric, receptor-coupled GTPases. GTPases, irrespective of their molecular structure, regulate a multitude of cellular functions and mediate the response to external stimuli. Aberrant GTPase activity has also been implicated in various pathological conditions [176]. Rac, a small protein that belongs to the Rho family of GTPases, acts as an activator of NADPH oxidase that generates ROS for various purposes [177]. Although ROS production is recognized as an important mechanism underlying hemolysis and eryptosis, very little is known about Rac function in RBCs. George et al. reported that Rac1 and Rac2 are essential for the structural integrity of the murine RBC cytoskeleton and are implicated in sickle cell formation [178]. In fact, the profound ROS accumulation characteristic of sickle RBCs has been demonstrated to be mainly caused by NADPH oxidase, which is regulated by Rac GTPase [179]. The first reported observation that GTPases influence eryptosis was by Attanzio et al., who showed that NSC23766, a specific inhibitor of Rac1 GTPase, was able to ameliorate oxysterol-induced eryptosis [180]. Moreover, Paone et al. also showed that Rac1 GTPase facilitates the invasion of RBCs by *Plasmodium* parasites and their subsequent development [181].

Since the participation of GTPases in RBC health has only recently gained momentum, much remains to be studied about the roles and interactions of GTPases in response to stress stimuli and in various clinical conditions.

### Cellular energy metabolism and eryptosis

In principle, erythrocyte metabolism takes place in a single reaction chamber that exchanges the different metabolites, i.e., the reaction educts and products of the metabolic pathways, with its surroundings in a strictly regulated manner. As erythrocytes lack intracellular organelles, their metabolism is restricted to well-known cytosolic biochemistry: glycolysis, the pentose phosphate pathway (PPP), redox metabolism, oxygen metabolism, purine/nucleoside metabolism, and membrane transport. In addition, the methionine salvage pathway, the glyoxalase system, carnitine metabolism, and remnants of the carboxylic acid metabolism were described in erythrocytes, and a recent review compiled a map of erythrocyte metabolism that listed 83 enzymes and membrane transport systems as well as 129 metabolites and ions [182]. Glucose, the most important metabolite and the unique energy source, is transported into human erythrocytes by glucose transporter 1 (GLUT1). Interestingly, this protein also accounts for dehydroascorbic acid transport through the erythrocyte membrane [183]. After entering the RBC, glucose is immediately converted to glucose-6-phosphate by hexokinase (HK) and then fueled into the glycolytic pathway or into the PPP [182]. Thus, it is not astonishing that one trigger of eryptosis is glucose depletion from the culture medium [80]. The impact of energy deprivation on the signaling pathways of erythrocytes was later disclosed in a mechanistic study: here, the authors demonstrated that removal of extracellular glucose and blockade of glycolysis by 2-deoxyglucose leads to fast depletion of cellular ATP. ATP depletion stimulates PKC-alpha activity and leads to translocation of the enzyme to the plasma membrane, thereby enhancing serine phosphorylation of membrane proteins, such as the Ca^2+^-permeable cation channel (Fig. 2). The increase of intracellular Ca^2+^ then leads to PS exposure and erythrocyte shrinkage [155]. These results point to the interconnection between glycolysis (the central metabolic pathway in erythrocytes), energy metabolism, and cation channel-driven eryptosis. Another study by Föller et al. confirmed the tight regulation of eryptosis by sensing the energy balance of the erythrocytes. Erythrocytes from knock-out mice that do not express the energy-sensing enzyme AMPK are significantly more susceptible to the eryptotic effect of energy depletion than erythrocytes from wild-type littermates expressing normal levels of AMPK [149]. Other inducers of erythrocyte death and concomitant ATP depletion include metals such as chromium [184], metalloids such as arsenic [185], riboflavin and UV light during RBC storage [186], and the phospholipid signaling molecule lysophosphatidic acid [187]. However, the energy balance is not the only juncture between metabolism and eryptosis. Interference with the pentose phosphate pathway by directly targeting G6PD using the antiinflammatory compounds dimethyl fumarate or parthenolide leads to complete depletion of GSH and concentration-dependent eryptosis [113]. It is therefore suggested that directly inhibiting enzymes of the PPP induces oxidative stress and thereby ROS-mediated eryptosis (Fig. 2). Last but not least, it was shown recently that d-ribose drives non-enzymatic glycation of hemoglobin [188], thereby interfering with oxygen loading and unloading [182] and inducing erythrocyte damage and eryptosis [188]. Thus, hemoglobin metabolism might play a role in iron-overload-driven death of erythrocytes.

### Eryptosis vs apoptosis: differences in signaling pathways

Non-nucleated organelle-free erythrocytes differ from the nucleated cells in unique characteristics, including those related to the aspects of the cell death networking. Lack of organelles and therefore the mitochondria-associated apoptotic machinery has raised concerns that highly specialized terminally differentiated RBCs can undergo apoptosis. However, in 2001, a handful of groundbreaking research discoveries confirmed that the Ca^2+^ ionophore ionomycin could trigger an apoptosis-like cell death in erythrocytes associated with the appearance of apoptotic signs, including cell shrinkage, membrane vesiculation, or PS exposure [22, 23]. This cell death of erythrocytes was shown to be induced by common triggers of apoptosis in nucleated cells, such as oxidative stress and hyperosmolarity [79]. Despite the common morphological hallmarks and shared triggers, it has become clear that the signaling pathways might differ. Initial studies demonstrated that although mature erythrocytes contain procaspase-3 and procaspase-8, they are devoid of the mitochondrial apoptotic machinery, e.g., APAF-1, cytochrome c, and caspases-2, -6, -7, and -9 [22, 23]. Moreover, caspases were found not to be recruited in response to multiple proapoptotic stimuli [189]. Indeed, further studies have shown that, unlike apoptosis, caspase-3 is not crucial for eryptotic cell death [84]. Caspase-3 is known to be activated by ROS [54, 78] and promote PS externalization in eryptosis [135, 136]. Nevertheless, PS translocation to the outer leaflet of the phospholipid bilayer, a hallmark of eryptosis, can frequently occur without caspase-3 recruitment, primarily in response to Ca^2+^ overload [84]. Intriguingly, in 2005, Mandal et al. demonstrated that erythrocytes possess Fas, FasL, FADD, showing that erythrocytes could form the DISC downstream of which caspase-8 and caspase-3 were activated [136]. This study paved the way for further research on the extrinsic apoptotic pathway. Of note, except for the Fas/FasL system, mature erythrocytes lack death receptors, including TNR-R1 (CD120a), TNR-R2 (CD120b), TRAIL-R1 (DR4), TRAIL-R2 (DR5), etc. [84], which play an important role in initiation of the extrinsic apoptosis pathway in nucleated cells [190].

Since apoptosis is defined by the NCCD as a caspase-dependent RCD [4], and considering the fact that eryptotic pathways don’t culminate in activation of caspases [84], it has become clear that eryptosis is guided and executed alternatively. In particular, morphological hallmarks of eryptosis, like PS externalization, cell shrinkage, and membrane blebbing, are orchestrated by Ca^2+^ influx [191]. Elevation of intracellular Ca^2+^ activates scramblase to ensure PS externalization, opens the Gardos channels, resulting in K^+^ efflux and subsequent cell shrinkage, and mobilizes calpain, leading to cytoskeleton degradation and membrane microvesiculation [78]. Undeniably, Ca^2+^ fluxes are crucial for almost all reported RCD pathways in erythrocytes, whereas in apoptosis, Ca^2+^ elevation mediates primarily the mitochondrial outer membrane permeabilization (MOMP), triggering the intrinsic apoptosis [192]. It is worth noting that the evolution of Ca^2+^ signaling dates back to prokaryotes, making it an important intracellular messenger regulating multiple cellular functions in all domains of life [193–195]. Given the ancient origin of the Ca^2+^-based signaling, which evolutionary developed earlier than the mitochondrial apoptotic toolkit whose emergence cannot be dated back to the times earlier than eukaryogenesis and acquisition of an endosymbiotic mitochondrion, it can be assumed that the Ca^2+^ toolkit takes over a key role in cell death signaling in terminally differentiated mitochondria-free erythrocytes. Thus, it can be hypothesized that the critical importance of Ca^2+^ signaling in eryptosis is linked to the expulsion of the nucleus and the organelles (primarily mitochondria) in precursors of erythrocytes during erythropoiesis.

Enucleated RBCs lack transcriptomes but contain long DNA fragments of both nuclear and mitochondrial origin, some retained transcripts, or microRNA molecules, and can acquire exogenous nucleic acids [196, 197]. However, unlike apoptosis [198–200], eryptosis probably cannot be regulated at the epigenetic, transcriptional, and translational levels.

Oxidative stress has been highlighted as an important contributor to both apoptosis [201] and eryptosis [202]. However, it is important to note that erythrocytes generate ROS primarily as a result of (i) hemoglobin oxidation, which can be considered as an iron-mediated Fenton reaction, (ii) the activity of NAPDH oxidase and (iii) xanthine oxidoreductase, while in nucleated cells ROS are mainly generated in the mitochondrial electron transport chain. All the above-mentioned sources of ROS in erythrocytes have been shown to contribute to eryptosis [54, 180]. Unlike nucleated cells, RBCs cannot cope with oxidative stress by upregulating the antioxidant enzymes. In general, ROS signaling in eryptosis is less diverse compared to nucleated cells, since in erythrocytes, ROS mainly mediate PS externalization by modulating Ca^2+^ influx, while in nucleated cells, there are multiple signaling pathways downstream of ROS involved in the execution of apoptosis.

Eryptosis can be regulated by several kinases, which can also be involved in apoptosis in nucleated cells. Surprisingly, there is abundant experimental evidence that CK1α, PKC, and JAK3 promote eryptosis, while in non-erythrocytic cells, these kinases are mostly antiapoptotic. In theory, the difference in the effects might be attributed to a limited set of upstream and downstream effector proteins in erythrocytes compared with nucleated cells [84].

Pyrshev et al. reported that differences between apoptosis and eryptosis could be extended to the level of plasma membranes [203]. Due to the absence of the network of intracellular membranes in erythrocytes compared to nucleated cells, the latter are characterized by a sharp decrease in lipid order in apoptosis, whereas eryptotic erythrocytes can preserve a relatively high lipid order. More dramatic changes in lipid order in apoptosis are explained by the exchange of cholesterol and phospholipids between membranes in nucleated cells, which is impossible in erythrocytes.

Thus, despite the similarity in functions and morphological signs, eryptosis and apoptosis differ in signaling pathways. The less diverse cell death signaling network in erythrocytes is associated primarily with expulsion of their organelles during maturation and makes Ca^2+^ signaling a master regulator of eryptosis, while the caspase-dependent apoptotic machinery is more sophisticated and redundant.

## Physiological functions of eryptosis

### Eryptosis: physiological role

**Fig. 3:**
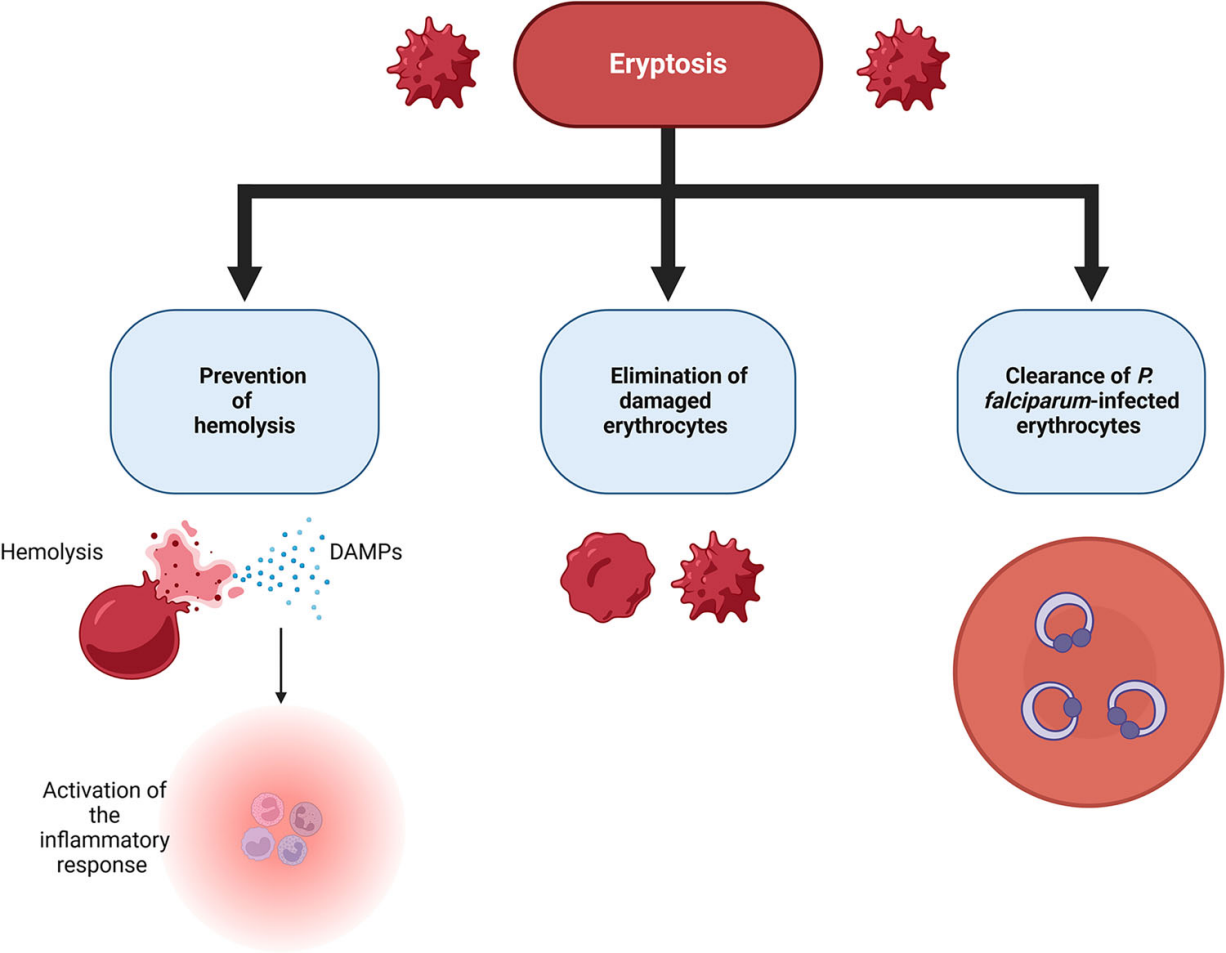
Eryptosis prevents hemolysis and ensures the clearance of damaged red blood cells. Hemolysis is associated with the release of DAMPs, which in turn can promote the innate immune response. Furthermore, eryptosis facilitates the elimination of *P. falciparum*-infected erythrocytes. DAMPs damage-associated molecular patterns. Created with Biorender.com.

Eryptosis is a part of the body defense system that leads to the shortening of the lifespan of damaged erythrocytes, subsequently removing them from circulation (Fig. 3). Different stress conditions, including energy depletion, oxidative stress, and hyperosmolarity which emerge in many diseases and some genetic disorders, lead to shortening the erythrocyte lifespan thus facilitating the removal of the damaged and defective erythrocytes [134]. In aged erythrocytes, on the other hand, a high intracellular Ca^2+^ level likewise plays a particular role in the initiation of different apoptotic pathways. The process of the removal of old erythrocytes from the circulation is also known as senescence, and similar to eryptosis, this process may be preferentially executed due to oxidative stress, hyperosmolarity, and energy depletion [24, 134]. Physiologically, and similar to apoptosis of nucleated cells, eryptosis occurs to ultimately eliminate damaged senescent, and damaged or dysfunctional mature erythrocytes from the circulation [204].

Hemolysis is the unwanted death of erythrocytes, as during hemolysis hemoglobin and other intraerythrocytic molecules are released into the bloodstream, which can impede renal filtration and result in renal failure [205]. In addition, the accumulated hemoglobin and its derivatives rapidly reduce NO availability in the circulation. The reduction in NO may result in different pathophysiological events like vasoconstriction, high adhesion, and endothelial activation [206]. The adhesion molecules may initiate the inflammatory process by activating the release of proinflammatory cytokines and chemokines [207]. Alternatively, eryptosis removes the defective erythrocytes prior to hemolysis, and thus, the pathological consequences associated with hemolytic activity can be avoided. Eryptosis is a physiological process, but uncompensated eryptosis may lead to anemia; thus, a normal erythrocyte count requires a balance between antieryptotic and proeryptotic mechanisms [208, 209].

Eradication of malaria is challenging due to the development of resistance against the drugs. Enhanced eryptosis has been suggested as a novel solution to control malarial parasite growth in the erythrocytes. Infected erythrocytes undergo oxidative stress, which in turn causes activation of calcium-dependent cation channels and facilitates Ca^2+^ entry into the erythrocyte. Triggered eryptosis eventually clears the infected cells from the bloodstream [210]. Similarly, maintenance of tissue oxygenation is highly dependent upon the balance between eryptosis and hematopoiesis [211]. Any deregulation of these two regulatory processes may affect proper oxygen transport to the tissues.

Fetal hemoglobin has a high oxygen affinity that facilitates increased oxygen supply in the intrauterine environment [89]. However, erythrocytes of newborns are resistant to various triggers of eryptosis, but they are highly susceptible to oxidative stress and eryptosis, so after birth, replacement of fetal hemoglobin with adult hemoglobin is functionally essential [212].

### Clearance of eryptotic cells

Multicellular organisms lose cells by different genetically controlled processes, leading to cell death, such as apoptosis and necroptosis. In the end, the dying or dead cells have to be removed from the body by clearance mechanisms, and the biomolecules of the cellular scaffold need to be recycled or excreted. Current concepts for the clearing of dead cells, especially RBCs, put the phagocytic macrophages right in the center [213, 214]. To ensure the regulated clearing of dead cells, it was suggested that dead cells express “eat-me” signals and lose “don´t-eat-me” signals on their surface [213]. Early “eat-me” signals described in the literature are immature sugar structures that lack sialic acid, and the asialoglycoprotein receptor was found to be responsible for the targeted deposition of apoptotic hepatocytes [215]. It was shown recently that treatment of erythrocytes with bacterial neuraminidase leads to desialylation of the glycocalyx and provokes cell surface PS exposure [216]. PS exposure, on the other hand, represents another “eat-me” signal that facilitates removal of eryptotic cells from the circulation by macrophages [77, 217]. This clearance mechanism has also been described in the context of different cellular stressors leading to PS exposure, e.g., hyperosmotic stress [217], plasmodium infection [77], oxidative stress [72], and, to some extent, also ageing of erythrocytes [218]. Thus, PS-dependent removal of erythrocytes has been described as efferocytosis. In addition, organisms also developed an immune system-based clearance mechanism, which is mainly operable in the case of erythrocyte senescence. This concept is based on the generation of neo-antigens on the surface of old erythrocytes. Germline-encoded naturally occurring autoantibodies (NOAbs) then bivalently bind to these structures on the cell membrane, thereby becoming necessary to selectively clear aged RBCs [219]. Importantly, IgG NOAbs to senescent erythrocytes are often directed against their most abundant integral membrane protein, the anion-transport protein, also known as AE1 [220]. Besides eryptotic PS exposure- or immune-mediated clearance, there are also lysis-dependent clearance mechanisms. For example, RBC degradation may be driven by extracellular histones that promote their aggregation and lysis in a concentration-dependent manner. Furthermore, these extracellular, normally nuclear proteins impair erythrocyte deformability and increase the adhesive properties of the cells. This, in turn, mediates their retention in the spleen and finally leads to hemolysis and removal of the cellular ghosts by macrophages [213, 221]. Taken together, tuned erythrocyte clearance is a prerequisite for balanced blood formation. It thus involves well-regulated mechanisms mainly executed by macrophages in the whole body [222].

### Does eryptosis occur prior to hemolysis to prevent it?

Eryptosis is clearly distinct from hemolysis, erythronecroptosis, a regulated counterpart of hemolysis, and erythrocyte senescence [28, 78]. Hemolysis, as an ACD of mature erythrocytes equivalent to necrosis, is an uncontrollable event developing in response to cellular injury and damage to cell membranes. Hemolytic conditions are associated with rupture of cell membranes and liberation of hemoglobin, its derivatives like methemoglobin or ferrylhemoglobin, heme, and other erythrocyte-derived molecules like ATP, IL-33, or HSP70 [62]. Haptoglobin and hemopexin molecules circulating in blood scavenge Hb and heme, respectively, to reduce their toxicity [223]. However, when the adaptive capacity is overcome, hemolytic erythrocyte-derived products act as signaling molecules, namely DAMPs, which promote inflammation via triggering TLR4-dependent activation of endothelial cells associated with NF-κB-mediated synthesis of proinflammatory cytokines, TLR4-independent, ROS-, NADPH oxidase- and heme iron-mediated promotion of neutrophil extracellular traps (NETs) formation, TLR4-dependent activation of macrophages accompanied by secretion of tumor necrosis factor α (TNF-α), and MyD88/TRIF pathway-mediated TLR4-dependent microglia activation that triggers neuroinflammation [224]. Furthermore, heme can drive TNF-α- and ROS-mediated necroptosis [225], heme oxygenase 1-dependent ferroptosis [226], and NLRP3 inflammasome-associated pyroptosis [227], which are highly proinflammatory RCDs. Moreover, liberation of erythrocyte-derived DAMPs has been linked with recruitment of the complement system [228]. Additionally, intravascular hemolysis results in renal injury, which develops as a consequence of filtering hemoproteins released from hemolytic red cells by the kidneys [229]. Thus, it seems logical that stressed erythrocytes should be cleared from circulation before hemolysis occurs to avert proinflammatory and highly detrimental lytic destruction.

Indeed, non-lytic eryptosis is considered one mechanism that ensures the elimination of erythrocytes prior to the occurrence of premature hemolysis [89, 230–232]. Robust evidence suggests that eryptosis does not lead to loss of membrane integrity [78]. Moreover, PS externalization, which is one culmination point of eryptosis signaling, is known to be immunosuppressive and antiinflammatory [233]. It should be noted that eryptotic RBCs are cleared from the circulation in an efferocytosis-dependent fashion within minutes, in contrast to senescent erythrocytes [77], which require days to be engulfed by macrophages and cleared [72]. This supports the hypothesis about the hemolysis-preventing role of eryptosis, since detrimental internal or external perturbations triggering eryptosis might continue to act, overcoming the adaptive capacity of cells, causing lysis. To prevent this, apoptotic cells are cleared as soon as possible. Recognition of externalized PS “eat-me” signals of eryptotic RBCs ensures a rapid and efficient cell removal.

### Immunogenic consequences of eryptosis: are DAMPs involved?

The abundance of erythrocytes in circulation suggests that immunogenic consequences of their cell death might dictate the immune response in disease, which is an underappreciated but important feature of erythrocyte biology. Hemolysis has been clearly associated with emitting proinflammatory signals through releasing DAMPs [224]. Moreover, necroptosis of erythrocytes is morphologically close to hemolysis, and the plasma membrane integrity is not preserved when erythrocytes undergo this lethal subroutine [28]. At the moment, there is no evidence that necroptosis of erythrocytes is associated with the release of DAMPs. However, the lytic nature of this cell death suggests that the liberation of the intracellular content is inevitable. To our knowledge, there is no evidence that DAMPs might be released in eryptosis, and this lethal subroutine of erythrocytes elicits DAMP-associated immunogenic consequences. On the contrary, like in apoptosis, the cell membrane remains intact in eryptotic cells, so that heme, hemoglobin, and other potential erythrocyte-derived DAMPs are preserved from passive cell membrane destruction-associated liberation [78, 91]. However, it is important to note that apoptotic cells can release DAMPs even in the absence of critical plasma membrane perturbations, in particular, via secretory lysosomes or exosomes [234, 235]. Mature erythrocytes are incapable of generating secretory lysosomes, but they produce extracellular vesicles (EVs) such as endosome-derived exosomes and plasma-membrane-derived microvesicles [236]. It has been reported that the generation of EVs in erythrocytes occurs in response to oxidative stress, Ca^2+^ overload, and the PS externalization-associated phospholipid membrane asymmetry [237]. Thus, eryptosis promotes the formation of EVs. Notably, EV-associated DAMPs have been found in stored red blood concentrates [238], suggesting that eryptosis-mediated generation of EVs might exert immunomodulatory DAMP-triggered effects. However, the issue is currently poorly studied to provide reliable evidence.

Since PS-expressing eryptotic erythrocytes are cleared by efferocytosis, this mechanism might elicit immunogenic effects as well. Efferocytosis of apoptotic cells is well documented to create an immunosuppressive and inflammation-resolving microenvironment, reprogramming macrophages [239, 240]. Uptake of both apoptotic and eryptotic cells via recognizing externalized PS molecules suggests that the effects on macrophage reprogramming might be similar. Indeed, PS-mediated uptake of erythrocytes promoted phenotype changes in monocyte-derived macrophages, contributing to recovery from experimental intracerebral hemorrhage. Notably, efferocytosis of eryptotic cells was associated with downregulation of proinflammatory cytokines and upregulation of antiinflammatory and antioxidant factors, including heme oxygenase 1 [241]. A growing body of evidence suggests that accelerated eryptosis might be an important player in hemorrhagic stroke and other intracranial hemorrhages [242, 243]. Thus, interactions between eryptotic and phagocytic cells like macrophages or microglia might modulate neuroinflammation in hemorrhagic conditions. To sum up, the immunogenic consequences of eryptosis are poorly understood. However, it should be emphasized that eryptosis is definitely involved in the regulation of the immune response, directly via modifying the macrophage phenotype or indirectly by preventing proinflammatory hemolysis. Elucidation of eryptosis-induced immunogenic effects might shed light on the pathogenesis of multiple diseases and identify novel therapeutic strategies.

## Eryptosis in disease

**Fig. 4:**
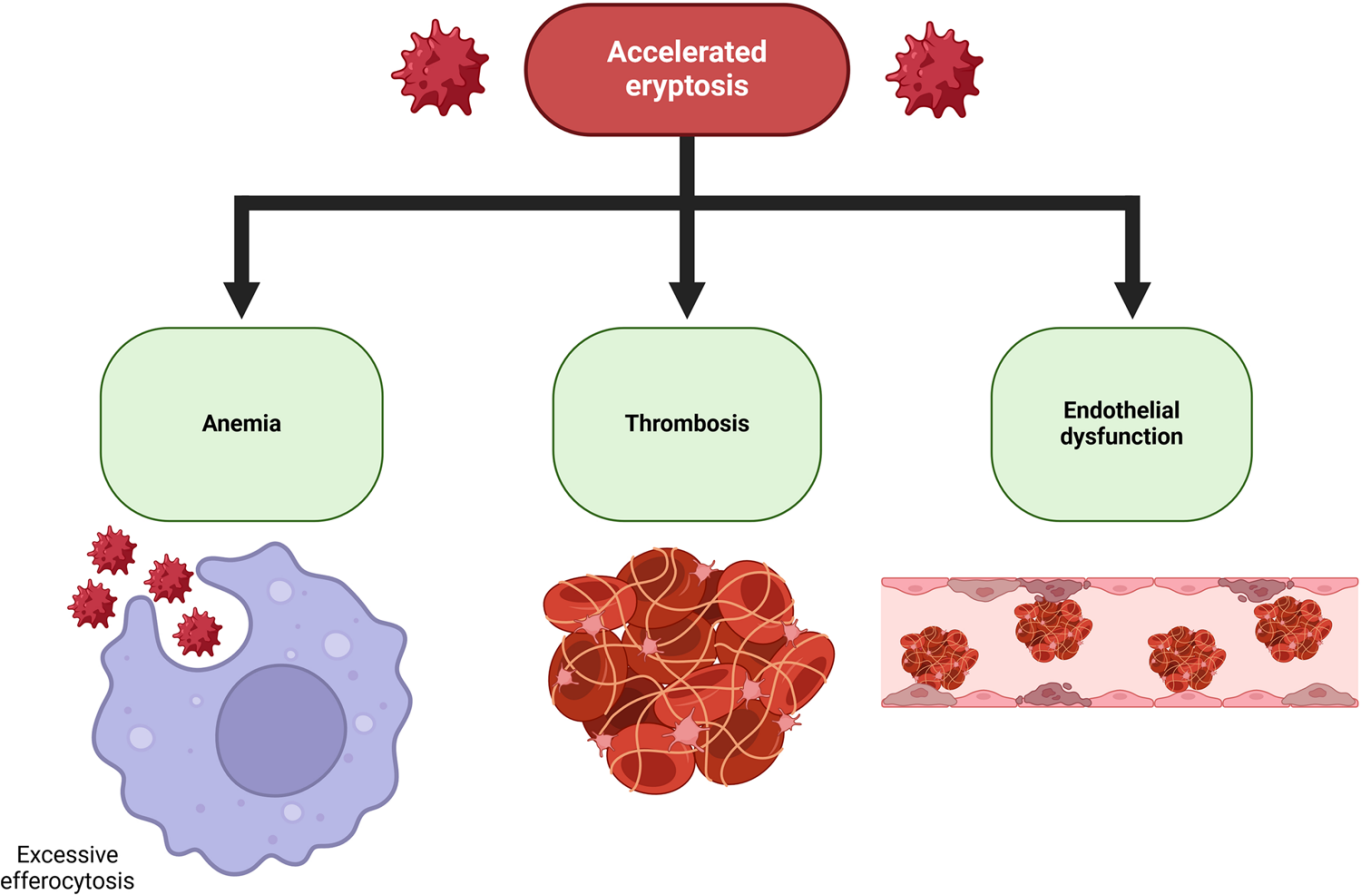
Enhanced eryptosis may lead to excessive clearance of eryptotic erythrocytes, resulting in anemia, promotion of blood clotting via phosphatidylserine-mediated mechanisms, and endothelial dysfunction associated with injury of endothelial cells. Created with Biorender.com.

A growing body of evidence indicates that multiple diseases are accompanied by enhanced eryptosis. Accelerated eryptosis might be associated with anemia due to rapid clearance of eryptotic cells via efferocytosis, activation of blood clotting, and damage to endothelial cells caused by adherence of eryptotic cells to them (Fig. 4).

### Renal diseases

As previously reported, enhanced eryptosis contributes to the pathophysiology of several clinical disorders. In this section, we focused on eryptosis in chronic kidney disease (CKD) and end-stage kidney disease patients (ESRD) treated by peritoneal dialysis (PD) or hemodialysis (HD).

In CKD patients, even though anemia develops primarily as a result of the decreased lifespan of RBCs caused by the low production of erythropoietin and iron deficiency, eryptosis seems to be a fundamental contributing factor [244–246]. In this population, different features, such as oxidative stress, inflammation, energy depletion, and uremic toxins, may exacerbate eryptosis [208, 231, 247]. All of these conditions get worse with the progression of kidney disease and the impairment of kidney function, thus stimulating RBC death and creating a vicious circle.

In recent years, different studies on CKD patients and animal models, such as mice, demonstrated that eryptosis, triggered by factors such as uremic toxins, inflammation, oxidative stress, and imbalances in calcium homeostasis, plays a fundamental role in the development and progression of renal anemia. In particular, dysregulation of eryptosis results in a premature RBC destruction and in a strong disequilibrium, worsening the hypo-proliferative character of anemia in kidney disease [108, 248–251]. Recently, Bonan et al. proposed a novel pathway for renal anemia based on an in *vitro* test evaluating the cytotoxic effect of uremic plasma on healthy RBCs and CD14^++^/CD16^+^ monocytes. They observed that serum collected from CKD patients induced eryptosis in healthy RBCs and promoted a proinflammatory monocyte phenotype [252].

Regarding the specific topic of uremic toxins, different studies have demonstrated that uremic toxins, such as indoxyl sulfate [253], acrolein [254], indole-3-acetic acid [255], urea, and *p*-cresol [256], may directly prompt eryptosis. Specifically, these molecules have been associated with a high rate of eryptosis in CKD. Indoxyl sulfate has been described to raise the cytosolic Ca^2+^ concentration and to stimulate erythrocyte cell membrane scrambling, thus amplifying the PS exposure on the surface of RBCs [253]. In the same study, these authors reported that indoxyl sulfate provokes the enhancement of ceramide levels, which is another well-recognized eryptosis-contributing factor [253]. In support of this data, Dias et al. postulated the role of indoxyl sulfate in the pathogenesis of renal anemia: indoxyl sulfate could stimulate oxidative stress and induce eryptosis through Organic Anion Transporter 2 (OAT2)- and NADPH oxidase-dependent, as well as GSH-independent mechanisms [257].

In a similar way, acrolein appears to stimulate oxidative stress damage, producing high levels of ceramide, which is accountable for the incrementation of cytosolic Ca^2+^ concentration and, consequently, the eryptosis levels [254]. In addition, in 2024, Kopera et al. hypothesized the central role of acrolein in the disruption of the structure of cell membranes in RBCs (changes in the cell membrane, cytosolic proteins, and osmotic sensitivity). They reported that the proportions of these variations on RBCs are dose-dependent [258].

Moreover, in patients with CKD, vanadate has also been observed to prompt eryptosis by inhibiting ATP production, thus resulting in energy deficit [259]. In addition, vanadate is also known to be able to inhibit glycolysis within RBCs [259]. All these observations on different uremic toxins were confirmed by in vitro studies: RBCs from healthy subjects were treated with different concentrations of the uremic toxins and at different time points. After exposition at different times and at different doses, eryptosis was evaluated. For example, Virzì et al. investigated the eryptosis rate in healthy RBCs treated with different concentrations of IL-6, IL-1β, urea, and *p*-cresol, comparable to their plasma levels in CKD patients, at different time points, and evaluated the cytotoxic effect on RBCs by an in vitro setting. These in vitro experiments showed that cytokines and uremic toxins reduce RBC viability and trigger eryptosis in a time- and dose-dependent manner [256].

These in vitro findings were confirmed by a recent study focused on 25 CKD patients. In this study, Clementi et al observed higher eryptosis levels in patients with advanced CKD (stage G4 and stage G5), compared with early stages of chronic renal damage (stage G1, G2, and G3) [260]. Furthermore, these authors reported a strong link between oxidative stress, inflammation, uremic toxins, and eryptosis [260]. In CKD patients, uremic toxins and ROS probably promote inflammation and oxidative stress through the stimulation of polymorphonuclear lymphocytes, leading to the release of inflammatory cytokines. All these factors are responsible for the impairment of RBC membrane structure in patients with CKD [260].

The impact of ESRD on eryptosis and underlying mechanisms was investigated over a decade ago. The data confirmed that in patients undergoing HD, eryptosis levels are higher as compared with healthy individuals [261]. Different studies investigated levels of eryptosis pre- and post-HD sessions [261–263]. Unfortunately, there were no consistent results from those studies: this may be due to the small patient cohort size in the different studies and due to the analysis of only a single or a small number of HD sessions. Interestingly, in the context of HD, Hefny et al. investigated the relationship between parathyroid hormone (PTH) and phosphorus, which are considered as uremic toxins, and eryptosis in 85 patients with CKD5d treated by HD [264]. They demonstrated by linear regression analysis that PTH was independently associated with the degree of eryptosis evaluated by flow cytometry and hypothesized that PTH could represent a new potential pathogenic mechanism linking hyperparathyroidism with renal anemia in HD patients [264].

In addition, Bissinger et al. confirmed the enhanced percentage of eryptosis in HD patients and reported a positive correlation between the percentage of circulating eryptotic cells and ROS and ceramide concentrations, and between the percentage of PS-exposing erythrocytes and both erythropoietin dosage and the percentage of reticulocytes [265].

These authors also compared eryptosis in PD and HD patients and reported its higher levels in CKD patients undergoing PD. In particular, in this subpopulation, eryptotic levels correlated with the dialysate volume. The authors hypothesized that glucose-based components of the dialysate could contribute to the stimulation of eryptosis [265]. On the contrary, Vos et al reported similar RBC survival in HD (*n* = 14) and PD (*n* = 5) patients using different laboratory techniques (label loss from RBCs associated with the chromium 51 labeling technique) [266]. Generally, the sample size of PD patients is small, and little can be concluded about the pathogenesis of eryptosis in PD patients [261, 265, 267].

The field has been facing a growing interest in the past decade. In particular, Virzì et al. confirmed the elevated eryptotic rate in stable PD patients compared to healthy control subjects and characterized PD patients (*n* = 46): important PD comorbidity such as diabetes mellitus (DM), arterial hypertension (AH), cardiovascular disease and main PD parameters (continuous ambulatory or automated PD), Kt/Vurea value ≤ 1.7 and >1.7, negative or positive history of peritonitis, do not have any influence. On the contrary, eryptosis showed significantly lower levels in PD patients with weekly creatinine clearance ≥45 L/week/1.73 m^2^ and in PD patients with residual diuresis (*n* = 23). In these 23 PD patients with residual diuresis, significant negative correlations between the percentage of eryptostic cells and residual glomerular filtration rate and diuresis volume were observed. These authors concluded that there is an increase in eryptosis levels with progressive residual diuresis and rGFR loss, probably due to decreased uremic toxins [268].

To better understand the association between eryptosis and inflammation in PD patients, the same group investigated eryptosis in PD-related peritonitis, the most common complication in patients treated by PD. As the first step, the eryptotic rate, as well as conventional and unconventional systemic inflammatory indices (C-reactive protein (CRP), IL-6, and IL-1β), was compared in 31 PD patients with diagnosis of acute peritonitis and 34 PD patients without any history of systemic inflammation and peritonitis in the last 3 months, as control group. On day 1 of peritonitis, the percentage of eryptotic cells was 3-fold higher in PD patients with peritonitis, and positive strong correlations were observed between all inflammatory indices and eryptosis degree. Consistently, Bester et al. studied the impact of IL-1β, IL-6, and IL-8 on the structure of erythrocytes and platelets and reported that all three ILs were found to be responsible for increased hypercoagulability of the whole blood. In particular, erythrocyte structure (visible structural membrane changes and eryptosis initiation) was notably affected by IL-8 [269]. In addition, CRP was classified as a trigger for eryptosis, and a strong association between CRP and eryptosis was confirmed in acute inflammatory conditions, such as peritonitis and acute appendicitis [270, 271]. All these observations were confirmed and supported by in vitro experimental data, including evaluation of cytotoxic effects on healthy RBCs [269–271]. Finally, Virzì et al., based on these previously obtained results, investigated the relationship between systemic eryptosis in PD-related peritonitis and specific peritonitis biomarkers in PD effluent (PDE), such as pWBC, pNGAL (Neutrophil Gelatinase-Associated Lipocalin), IL-6, and IL-1β. Significant positive correlations were observed between eryptosis level and all analyzed peritoneal biomarkers of peritonitis, highlighting a potential link between systemic effect (eryptosis) and local inflammation (peritoneum in PD-related peritonitis patients), and definitely confirmed that eryptosis is mostly influenced by blood composition [271]. Remarkably, the grade of eryptosis on day 1 of peritonitis does not reveal a direct correlation with the patient’s prognosis [271]. Furthermore, the confirmation of the induction of eryptosis by peritonitis was highlighted by an in vitro model. Intact RBCs collected from healthy donors were exposed to the plasma of PD patients with peritonitis, resulting in significantly accelerated eryptosis [271].

### Hepatic diseases

The liver is the major RBC bank and is fundamental for the RBCs' clearance and iron recycling [272]. In the steady state, RBC production is in equilibrium with RBC removal from circulation, which can be accomplished by two different mechanisms: hemolysis and phagocytosis by macrophages. Recent scientific reports highlighted that, in pathological conditions, the liver, but not the spleen, is the primary site of erythrocyte clearance [273, 274]. It was shown that aged or/and damaged RBCs are sequestrated in the hepatic sinusoid in a PS-dependent way, and the human sinonasal epithelial cells (HSECs) mediate the recognition and the binding of damaged RBCs in a PS-dependent manner via stabilin-1 and stabilin-2 (pro-phagocytic signals and ligand–ligand interactions) [275–277]. Based on these results, Sung-Jin et al hypothesized that stabilin-1 and stabilin-2 could play an important role in hepatic sequestration of PS-exposed RBCs, suggesting the potential mechanism for the clearance of damaged RBCs by Kupffer cells [273]. On the contrary, CD47 is demonstrated to be a “don’t-eat-me” signal. It has an important function in the maintenance of RBC homeostasis via the interaction with signal regulatory protein alpha (SIRPα) [278]. In addition, liver Kupffer cells rapidly removed 80% of RBC-derived vesicles from the circulation within 5 min, mainly via scavenger receptors [279]. Finally, eryptotic RBCs are engulfed and processed by Kupffer cells, the phagosome containing eryptotic RBCs merges with lysosomal vesicles, obtaining a complex called the erythrophagolysosome. The exposure of PS to the RBC membrane promotes phagocytosis and also affects coagulation: PS exposure of RBCs causes a diminution of coagulation times [280–282]. In particular, Xiaoming et al. studied the mechanism of thrombogenicity in cirrhosis patients, evaluating the relationship between PS on blood cells and endothelial cells and the hypercoagulable state. They indicated a shorter coagulation time and an increase in thrombin and fibrin formation [280].

During liver disease, anemia can occur after blood loss, infection, cancer, or nutritional imbalances. Recently, eryptosis and the consequent accelerated clearance of circulating RBCs were mentioned as the mechanisms involved in the development of anemia in patients with hepatic disease. Recent animal- and human-based studies described the enhanced percentage of eryptotic erythrocytes in patients with hepatic failure and hyperbilirubinemia [210, 250, 280, 283, 284]. In fact, many studies have reported the eryptotic effect of bile acids and bilirubin on RBCs, whereas albumin reduces the toxic effect of bilirubin on erythrocytes [210, 283]. Specifically, the increased loss of RBCs leads to the increased formation of bilirubin, which further triggers eryptosis by enhancing both Ca^2+^ influx, SMase activation, and ceramide production, thus creating a vicious cycle. Furthermore, incubation of erythrocytes with serum obtained from patients with liver disease induced eryptosis in relation to bilirubin levels. Patients with hyperbilirubinemia presented significantly lower erythrocyte counts and significantly higher reticulocyte counts compared to patients with low bilirubin levels [285]. Recently, Cheng et al. described that enhanced eryptosis is involved in anemia in hepatitis B-related acute-on-chronic liver failure (HB-ACLF). In particular, they observed that the percentage of eryptotic cells was significantly higher in patients with HB-ACLF in comparison with healthy donors, patients with chronic hepatitis B, and patients with cirrhosis. It is hypothesized that eryptosis in patients with HB-ACLF is, at least in part, stimulated by specific components in the plasma, causing accelerated eryptosis and contributing to the development of anemia [250].

Consistent with all these in vitro and in vivo findings, the liver could be identified as the major organ involved in the rapid erythrocyte removal and iron recycling.

### Neurological disorders

In conditions like Parkinson’s and Alzheimer’s diseases, disruptions in cellular Ca^2+^ regulating systems in the plasma membrane, ER, and mitochondria can manifest in synaptic dysfunction, decreased plasticity, and neuronal degeneration [286–288]. In particular, the production of calpain and ceramide is altered, which can trigger eryptosis in Parkinson’s disease [286, 287, 289]. Alzheimer’s disease is another neurodegenerative disease characterized by senile plaques in regions of the central nervous system. Amyloid-β peptide is the main component of Alzheimer's plaques that can cause neuronal cell death, and it can cause oxidative damage to RBCs. Amyloid-β peptide disrupts erythrocyte membrane phospholipids and reduces erythrocyte cell volume, as a result of ceramide formation [289].

### Cardiovascular and metabolic diseases

#### Eryptosis and oxidative damage in hypertensive patients

Some studies have linked oxidative stress with AH. Hypertensive patients have been observed to produce high levels of hydrogen peroxide, superoxide anion, and NO in leukocytes [290–292], exhibit elevated lipid peroxidation in erythrocytes, and display elevated concentrations of 8-isoprostanes in plasma [293]. Patients with moderate, untreated AH exhibited elevated levels of lipid peroxidation in erythrocytes when compared to normotensive individuals and treated hypertensive patients [294]. Additionally, they demonstrated increased concentrations of 8-isoprostanes in urine when compared to patients with treated AH [295]. In contrast, treated hypertensive patients with a disease duration of five to ten years exhibited a reduced level of lipid peroxidation in the blood [296].

Regarding the antioxidant response, untreated hypertensive patients exhibited a diminished concentration of GSH in erythrocytes [297, 298]. Furthermore, there was a reduction in the activity of CAT, SOD, and GPx in erythrocytes [293] and blood [296] as compared with normotensive patients. In contrast, treated hypertensive patients exhibited a higher concentration of GSH in the bloodstream relative to normotensive patients [299].

Oxidative damage has been linked to high levels of eryptosis through an increase in the intracellular free Ca^2+^ concentration, as evidenced by in vitro studies [24, 134, 300, 301] in patients intoxicated with lead [302], and in diabetic patients with chronic renal damage [303]. Patients with uncontrolled AH also exhibit elevated intracellular Ca^2+^ levels [304], although these authors did not investigate the potential role of oxidative damage or eryptosis in this context.

The elevation of intracellular Ca^2+^ is not the sole known pathway to produce higher rates of eryptosis [305]. In addition to oxidative stress, eryptosis can be induced by energetic, mechanical, and osmotic stresses.

#### Eryptosis and oxidative damage in diabetes mellitus and its complications

Type 2 diabetes mellitus (T2DM) is associated with several long-term complications, including tissue damage and several complex syndromes such as cataracts, renal dysfunction, nerve damage, atherosclerosis, and the shortened lifespan of erythrocytes [306]. Some studies have identified a correlation between T2DM and oxidative damage or antioxidant status [307–309]. In individuals with DM, chronic hyperglycemia may contribute to an increase in ROS/RNS. This oxidative insult has been demonstrated to impair pancreatic β-cell function and exacerbate insulin resistance, thereby contributing to the progression of T2DM [310]. In DM, an imbalance in the prooxidant/antioxidant equilibrium can result in damage to cellular macromolecules, leading to modifications in DNA and protein, as well as lipid peroxidation [307–309]. The mechanisms that may contribute to the induction of oxidative damage in diabetic patients include hyperglycemia-induced glucose auto-oxidation, non-enzymatic glycation of proteins and lipids, increased sorbitol pathway activity, oxidation of AGEs, and formation of prostaglandin H2 (PGH2) [311].

A decline in the body’s antioxidant defenses may be linked to an increased risk of complications associated with DM [311–313]. Furthermore, lipid peroxidation and antioxidant enzymes in the blood have been identified as markers for vascular injury and microangiopathy in DM [314]. An increase in oxidative damage in the diabetic kidney may induce apoptosis and contribute to the development of diabetic nephropathy [312, 313, 315]. The administration of antioxidants at the onset of experimental diabetes has been demonstrated to mitigate renal injury in diabetic subjects [316, 317]. Conversely, CKD may also induce oxidative damage in other pathological conditions [308, 318, 319].

It is postulated that DM-induced oxidative damage may be more significant in erythrocytes than in other cells, due to their high content of iron and polyunsaturated fatty acids, as well as their role as oxygen transporters, with a high degree of exposure to free radicals [320].

A recent study demonstrated that hyperglycemia in erythrocytes of patients with type 1 diabetes mellitus (T1DM) is associated with an increased percentage of circulating erythrocytes that expose PS at the cell surface. PS-exposing erythrocytes are recognized, bound, engulfed, and degraded by macrophages. Therefore, PS exposure, a characteristic of suicidal erythrocyte death or eryptosis, accelerates the clearance of affected erythrocytes from the circulating blood. Similarly, exposure to methylglyoxal, a reactive dicarbonyl compound formed as a metabolic byproduct of glycolysis, has been shown to inhibit glycolysis, leading to a reduction in ATP and GSH concentrations. Methylglyoxal impairs energy production and the anti-oxidative protective effects, thereby contributing to the enhanced PS exposure of circulating erythrocytes and ultimately resulting in anemia and microcirculatory disequilibrium [321]. Furthermore, elevated concentrations of beta2-microglobulin were associated with a slight but significant increase in PS externalization in human erythrocytes [322].

Other authors have reported that an increased exposure of PS on the surface of erythrocytes is present in both patients with chronic renal failure (CRF) who are not undergoing dialysis and in patients who are undergoing continuous ambulatory PD or chronic maintenance HD. Given that PS on the cell surface may trigger several reactions, this abnormality may contribute to the erythrocyte pathology commonly encountered in chronic uremia, including ionic transport defects, osmotic alterations, deformations, fragility, and coagulation abnormalities. Similarly, other studies indicate that enhanced PS exposure may result in a pathological erythrocyte procoagulant phenotype, which may contribute to the development of a hypercoagulable state. Furthermore, an inverse correlation was identified between the percentage of PS-positive erythrocytes and hemoglobin concentration. A logistic regression analysis identified the percentage of PS-positive erythrocytes as a risk factor for anemia. The present study has identified enhanced PS exposure at the surface of erythrocytes as a potential risk factor for anemia and a contributor to its development in patients with CRF undergoing PD. Furthermore, increased PS exposure is not exclusive to erythrocytes; it can also be acquired by other cells [267]. The externalization of PS in erythrocytes of patients with T2DM has yet to be studied. An increase in the eryptotic process may be associated with the early stages of CKD and the adverse outcomes observed in many diabetic patients.

### Autoimmune conditions

In certain autoimmune diseases, an accelerated rate of eryptosis can lead to a reduced count of circulating RBCs, consequently resulting in anemia. In systemic lupus erythematosus (SLE), antibodies such as anti-Sm and anti-RNP are associated with various hematological disorders, including hemolytic anemia [323, 324]. Anemia is commonly observed in patients with SLE, especially in the advanced stages of the disease. This condition is likely attributable to iron deficiency or autoimmune hemolytic anemia (AIHA), which results from increased eryptosis [325, 326]. SLE patients also have lower hematocrit levels compared to healthy individuals and exhibit a higher percentage of PS-exposing erythrocytes, elevated cytosolic Ca^2+^ levels, abundance of ROS, and significant erythrocyte shrinkage [327, 328]. Furthermore, when serum from SLE patients was incubated with RBCs from healthy donors, significant PS exposure and increased intracellular calcium influx were observed, although the cell size remained unchanged [328].

AIHA is another autoimmune disease that includes a group of disorders characterized by the production of autoantibodies that attack RBCs. In 2018, Bartolmas et al. investigated the warm and cold types of AIHA (wAIHA and cAIHA, respectively) and demonstrated that eryptosis is predominantly driven by cold IgM and/or IgA antibodies rather than warm IgG autoantibodies, as shown by increased PS exposure in response to autoantibodies [329]. A year later, the same group demonstrated that IgM-mediated eryptosis was dependent on C5 complement activation. Further studies demonstrated that C8 might support the formation of eryptotic cells, whereas C9 may not be involved. Additionally, heat inactivation of AIHA serum hampered PS exposure and inhibited eryptosis [330]. IgA autoantibodies in wAIHA did not seem to have the same effect described for the autoantibodies in cAIHA and did not induce RBC eryptosis unless the RBCs were opsonized [330]. The process of eryptosis in AIHA can be modulated by erythropoietin, which not only stimulates erythropoiesis but might also inhibit eryptosis, thus improving anemia [211, 330]. However, a sustained increase in erythropoietin leads to the generation of erythrocytes with enhanced sensitivity to some triggers of eryptosis [331].

Antiphospholipid syndrome (APS) is an autoimmune disorder characterized by the presence of antiphospholipid antibodies and a hypercoagulable state. Research has demonstrated that autoantibodies from APS patients, unlike those from asymptomatic antiphospholipid carriers, can induce eryptosis in RBCs from healthy donors in vitro. This suggests a significant role for these autoantibodies in the clinical manifestations and prothrombotic state observed in APS patients [332].

### Eryptosis in acute COVID-19 and long COVID

Acute COVID-19 is not simply a respiratory condition, but rather one that affects the vasculature of the patients. Acute COVID-19 is characterized by a significant dysregulation of molecules that may lead to endothelial disruption, platelet hyperactivation, microthrombosis, and compromised microcirculation [333–335]. The pathology prevalent in the acute phase of the disease can continue, evolving into persistent and long-term symptoms. During the aftermath of the acute COVID-19 pandemic, we are faced with a newly identified infection-associated chronic condition (IACC): Long COVID [336]. Long COVID, also known as post-acute sequelae of SARS-CoV-2 infection (PASC), is a chronic condition characterized by a range of persistent symptoms that last for weeks or months following the initial COVID-19 infection, affecting multiple organ systems and significantly impairing daily functioning and quality of life. Long COVID has emerged as a defining crisis of our time [336]. Affecting 400 million people globally and carrying an economic burden of $1 trillion per year, Long COVID has exposed the urgent need for a coordinated response strategy - one that is currently lacking worldwide [337]. Excess deaths are on the rise, with clotting pathology playing a potentially deadly role, not only in the persistence of Long COVID symptoms but also in contributing to silent dangers such as the onset of new immune and autoimmune diseases, heart attacks, strokes, and sudden, unexpected deaths, even in individuals as young as their twenties.

Endotheliopathies and significant clotting pathologies are important clinical features of acute COVID-19 [338–340]. During acute infection, SARS-CoV-2 can bind to endothelial cells, altering their function and initiating clot formation through platelet activation and fibrinogen binding. This contributes to tissue damage, inflammation, and the formation of NETs, exacerbating thrombosis and significantly interfering with blood rheology. There have also been reports of complement deposition on circulating erythrocytes from hospitalized COVID-19 patients using flow cytometry [341]. In addition, it has been debated whether erythrocyte membrane injury could promote the thrombotic complications frequently observed in COVID-19 patients [342], and that reduced deformability of erythrocytes may contribute to inflammation and hypoxia in COVID-19 patients [343]. Additionally, changes in red cell distribution width (RDW) have been observed, with substantial increases during acute COVID [344–348]. Increased levels of inflammatory secretory phospholipase A_2_ IIA (sPLA_2_-IIA) have also been noted in acute COVID-19 [349]. sPLA_2_ could metabolize phospholipids in platelets, erythrocytes, and endothelial cells to produce arachidonic acid and lysophospholipids [349].

**Fig. 5:**
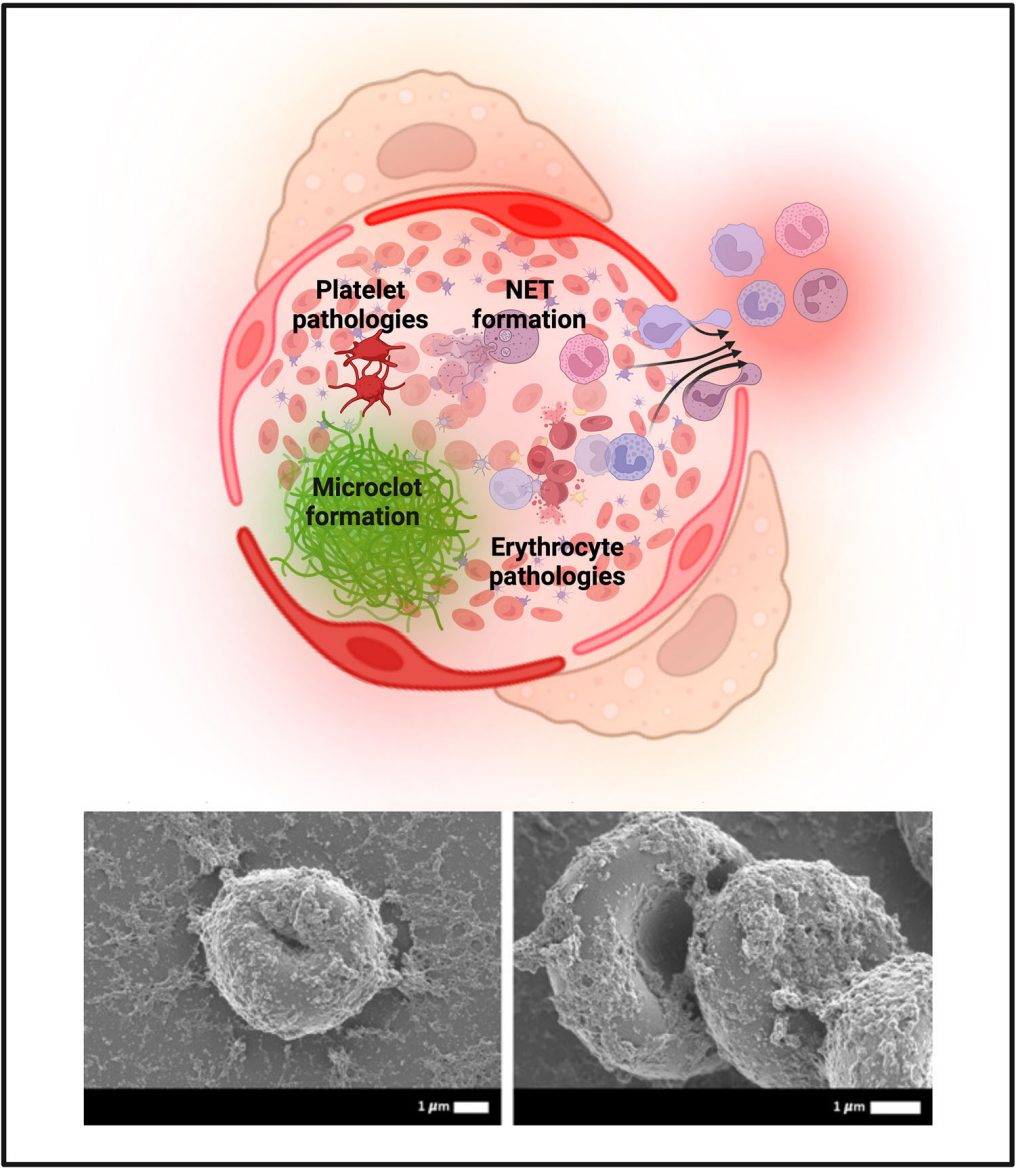
Long COVID is associated with circulating inflammatory molecules that contribute to endothelialitis, microclot formation, platelet hyperactivity, and damage to erythrocytes, which may impair blood rheology and result in inadequate microcirculation, leading to ischemia-reperfusion injury (above). Scanning electron microscopy shows erythrocytes from Long COVID patients are covered by fibrin amyloid microclots (below). Created with Biorender.com.

As Long COVID is a continuation of pathologies found in the acute phase of the disease, it can be suggested that erythrocyte pathologies noted in acute COVID-19 may continue or even be exacerbated in Long COVID. Endothelial dysfunction and microclot formation have also been proposed as central components of Long COVID and the microvascular nature of the disease [336, 350]. Plasma of Long COVID patients carries a significant load of amyloid microclots [350–356], and these microclots may cause damage to erythrocytes (Fig. 5). The intravascular formation of NETs is also central in Long COVID [357, 358]; and NETs, in turn, is a known trigger for coagulation and blood vessel occlusion [359], and also involve significant erythrocyte pathologies, specifically related to rheology and oxygen carrying capacity and ultimately resulting in ischemia-reperfusion injury [352]. The spike protein may also interfere with blood flow, as the spike protein can directly bind to fibrinogen [360]. It can thus affect blood rheology [361].

Studies show persistent elevation of vascular injury markers such as vWF and cytokines, suggesting ongoing thromboinflammation and hypercoagulable states in Long COVID [362, 363]. Additionally, disruptions in the blood-brain barrier in those with neurocognitive symptoms have been observed, as well as hypoperfusion in various organs, contributing to end-organ dysfunction [364, 365]. Exercise studies have revealed abnormal amyloid deposits in muscle biopsies from Long COVID patients, indicating the potential role of microvascular disease in persistent symptoms [366].

The size, shape, and deformability of erythrocytes are crucial to their function, and abnormalities in these traits are seen in conditions like rheumatoid arthritis and T2DM. Since these diseases are common comorbidities of Long COVID, similar erythrocyte changes are likely to occur in individuals with Long COVID who also suffer from these conditions. While the exact causes of these erythrocyte changes are due to fibrin amyloid microclots or other factors, such as ROS (which can cause RBC changes as well [367, 368]), remain unclear, they likely contribute to the oxidative stress observed in Long COVID [369, 370]. Notably, erythrocytes from other post-viral conditions like ME/CFS have been found to be significantly more non-discocytic [371] and stiffer [372] as compared with erythrocytes from healthy individuals. Similar abnormalities are seen in erythrocyte sedimentation rates (ESR) [372, 373], as well as in other conditions such as delayed cerebral ischemia following subarachnoid hemorrhage [374]. While RDW is easily measurable, studies examining RDW in Long COVID are still lacking, suggesting this marker could be more widely utilized [352]. One severe consequence of oxidative stress, leading to erythrocyte stiffness and morphological changes, is eryptosis [208, 230, 232, 375]. Erythrocyte pathology clearly indicates microcirculatory problems [208, 230, 232, 375], supporting the hypothesis that inadequate microcirculation in Long COVID, leading to ischemia-reperfusion injury, could play a critical role [352].

### Eryptosis in sepsis

Sepsis is described as the clinical syndrome of multi-organ dysfunction and immune dysregulation originating from a dysregulated host response to infection. It is characterized by high hospital costs, morbidity, and mortality, and it provokes a high hospitalization rate and inpatient mortality [376–378]. The pathogenesis and the pathophysiology of sepsis is very complicated and multifaceted: numerous biological mechanisms, such as infection, inflammation, oxidative stress, immune system dysregulation, blood coagulation, endothelium dysfunction, tissue damage, elevated cellular death and/or apoptosis are involved during sepsis at tissue, cellular and molecular levels causing a damaging systemic reaction [379].

A strong, uncontrolled, and dysregulated inflammation is reported in septic conditions, and it is highly correlated with sepsis-induced anemia. In the course of the infection, pathogens need iron to sustain their survival; therefore, they may attack RBCs, inducing membrane structure changes (erythrocyte shrinkage, membrane blebbing, and PS exposure) and, finally, eryptosis. It results in a higher percentage of degraded RBCs, thereby reducing the RBC count and causing anemia during sepsis (eryptotic RBCs were degraded and cleared from circulation) [202, 230, 380].

Recently, different in vitro and in vivo studies reported sepsis-induced changes in the shape of human erythrocytes, which have also been confirmed in animal models and humans [126, 381–389]. For example, Piagnerelli et al. observed that patients with sepsis show higher activity of neuraminidase, which affects erythrocyte rheology [381]. In addition, extensive modifications in RBC morphology and shape triggered by oxidative stress were noted in Brazilian septic patients [385].

Studies on animal models confirmed these observations. In mouse models of cecal ligation and puncture (CLP)-induced sepsis, plasma-derived EVs affected RBC deformability, increasing RBC rigidity [387]. Similarly, in rat models of CLP‑induced sepsis, oxidative stress influenced the rheology of blood‑influenced RBC deformability and the relative resistance [384]. Kempe et al. performed an experimental study and determined PS exposure, RBC volume, cytosolic Ca^2+^ activity, and ceramide formation (flow cytometer evaluation for all parameters). In particular, in vitro eryptosis was induced in RBCs from healthy volunteers after exposure to plasma from septic patients [126]. These results were corroborated and deepened by Marcello et al. in a case–control in vivo and in vitro study. They specifically investigated the in vitro induction of eryptosis on healthy RBCs exposed to septic plasma at different time points and evaluated the relationship between eryptosis percentage and Endotoxin Activity Assay (EAA) levels, mortality, and other biological markers of inflammation and oxidative stress [383]. The Endotoxin Activity Assay is a chemiluminescent methodology providing a prompt tool to determine a patient’s endotoxin level. An elevated level of EAA is a significant risk factor for severe sepsis and is associated with increased organ dysfunction and mortality [390]. In vitro results highlighted dramatic alteration of morphology and increased levels of eryptosis in healthy RBCs incubated with septic plasma: the sepsis-induced cytotoxic effect reached its maximum value at 15 min. A follow-up in vivo study confirmed these data: septic patients showed a higher percentage of annexin V binding RBCs compared to healthy subjects. The authors also reported a significant correlation between eryptosis and other biological markers of inflammation and oxidative stress [390]. In summary, there is growing evidence suggesting a relationship between eryptosis and septic prognosis or severity.

### Eryptosis and cancer

Bissinger et al. in 2016 conducted the only in *vivo* study that establishes a link between eryptosis and a specific type of cancer [391]. The study reports a significant association between lung cancer (LC) and suggests that the anemia observed in LC patients is not primarily due to diminished erythropoiesis, but rather to an increased turnover of erythrocytes. Although erythropoiesis is enhanced, anemia likely continues due to elevated erythropoietin levels, which paradoxically heighten the vulnerability of RBCs to eryptosis. Eryptosis is characterized by the exposure of PS on the membranes of RBCs, facilitating their clearance from circulation and contributing to anemia in both treated and untreated LC patients. In patients receiving cytostatic therapy, the increase in PS exposure coincided with a rise in RBC volume, which is atypical for eryptosis, typically associated with cell shrinkage. Additionally, the cells showed elevated levels of ceramide at the plasma membrane surface. The study also suggested that oxidative stress, a known trigger of eryptosis, may contribute to the membrane alterations observed in RBCs of LC patients.

Cytostatic agents, particularly platinum-based drugs such as topotecan and cisplatin, have been linked to the induction of eryptosis, thereby worsening anemia [392, 393]. This increased eryptosis may also disrupt microcirculation by facilitating coagulation, as PS-exposing erythrocytes can adhere to endothelial cells and activate clotting processes [208]. Given that LC patients are already at an enhanced risk for thrombosis, these mechanisms may lead to poorer clinical outcomes. In summary, this study underscores the pivotal role of eryptosis in the anemia associated with LC, particularly in patients undergoing cytostatic treatment. The findings suggest that targeting eryptosis could offer a therapeutic strategy to mitigate anemia in LC patients. However, caution is warranted as inhibiting eryptosis could potentially interfere with the apoptosis of tumor cells, which might diminish the effectiveness of cytostatic therapies. Conversely, addressing anemia could reduce erythropoietin levels, a factor known to promote tumor progression and angiogenesis.

As discussed in the section on inducers of eryptosis, numerous antitumor agents have demonstrated proeryptotic activity in vitro. Among these, one of the most discussed is Tamoxifen. The proapoptotic effects of Tamoxifen are supported by studies indicating that increased intracellular Ca²^+^ and ROS levels can activate various signaling pathways that facilitate the process of eryptosis [394–396]. This is particularly relevant in the cancer context, where the tumor microenvironment often promotes oxidative stress and altered cellular signaling pathways, contributing to the rapid turnover of RBCs.

### Eryptosis and malaria

Malaria is an ancient vector-borne zoonotic infection caused by *Plasmodium* species. Every year, approximately 300-500 million people suffer from malaria globally, and 94% of those cases are reported in the African region [210, 397–399]. *Plasmodium falciparum* is considered one of the most virulent types among the different malaria species. *Plasmodium falciparum* is primarily responsible for a significant part of the morbidity and mortality that comes from malarial infection [400]. Infection with *P. falciparum* may be asymptomatic. However, it can be manifested with the classical form of malarial fever, cerebral malaria, or malaria-induced anemia (MIA) and is responsible for one-third of malaria-associated deaths [401]. The blood hemoglobin levels after *P. falciparum* infection drastically come down to 5 g/dL [400, 402]. Several factors can trigger anemia in malaria patients, including reduced erythrocyte generation in bone marrow, extravascular destruction or hemolysis of erythrocytes, or suicidal death of erythrocytes familiar as eryptosis [210, 403, 404].

Blood or intraerythrocytic parasites like *Plasmodium* induce oxidative stress by generating ROS in the patient’s erythrocytes. Furthermore, it has been reported that in comparison to normal erythrocytes, *Plasmodium*-infected erythrocytes produce twice as many free radicals like hydrogen peroxide (H_2_O_2_) or hydroxyl (OH^-^) [405]. Similarly, neutrophils and macrophages become activated as part of the host defense system as well as the inflammatory response, which produces a large amount of RNS and ROS, followed by the entry of these ROS into the erythrocytes [406]. Increased erythrocyte oxidative stress results in the opening of multiple ion channels, comprising the nonselective cation channels (NSCC), like sodium, potassium, and calcium channels [202, 404]. Cellular entry of Na^+^ and Ca^2+^ through their respective channels is essential for the parasite’s optimal growth and reproduction [404]. Again, increased cytosolic calcium is one of the most crucial stimulators of eryptosis [407]. Eryptotic erythrocytes are identified by the externalization of the membrane PS, which attracts the phagocytic cell-like macrophages [230, 404], followed by engulfment and destruction of both the host cell and intraerythrocytic pathogen [404]. Thus, malaria-infected erythrocytes experience eryptosis, which has also been reported in several previous investigations, confirming that eryptosis may, therefore, be a critical defense mechanism against malarial infection or parasitemia. In this sense, several diseases or conditions or genetic disorders that are predisposing to eryptosis, including deficiency in glucose-6-phosphate dehydrogenase, sickle cell anemia, beta-thalassemia trait, iron insufficiency or deficiency of carriers like GLUT1, AE1 are known to provide a certain degree of defense against a severe malarial course [230, 404]. Furthermore, several xenobiotics that have been shown to promote eryptosis, like lead, paclitaxel, cyclosporine, prostaglandin E_2_ (PGE_2_), curcumin, amphotericin B, dimethylfumerate, and chlorpromazine, can slow down the progression of malarial infection in mice or humans [230, 375, 404, 408–411].

### Eryptosis and inherited blood disorders

Eryptosis is observed in conditions like sickle cell anemia [80], thalassemia, glucose-6-phosphate dehydrogenase deficiency [412], among other hemolytic diseases. In sickle cell anemia oxidative stress can trigger eryptosis and the release of red blood cell microparticles (RBC-MPs), which are small phospholipid-containing vesicles implicated in physiological processes such as hemostasis and potentially linked to macrovascular dysfunction. Nader et al. demonstrated that sickle cell anemia correlates with elevated eryptosis markers, such as PS externalization, increased ROS within RBCs, and elevated intracellular calcium levels. Additionally, there is an increase in RBC-MPs [413]. Treating cells with cumene hydroperoxide was shown to enhance eryptosis, whereas the antioxidant NAC mitigated this process by enhancing RBC deformability and reducing levels of ROS, calcium, and PS exposure, and demonstrated that oxidative stress plays a crucial role in eryptosis, with pro-oxidants promoting and antioxidants limiting eryptotic processes [413]. In hereditary spherocytosis (HS), the exposure of PS is more prevalent compared to α- and β-thalassemia, particularly in younger erythrocytes [414]. Aged cells in thalassemia exhibit increased loss of PS asymmetry and are associated with greater loss of sialic acid and GlcNAc-bearing glycoconjugates. These factors are crucial in the premature clearance of erythrocytes from circulation, a mechanism similar to that in HbE/β-thalassemia [414].

### Eryptosis and aging

Multicellular organisms are subject to the biological process of aging, and recently 12 different hallmarks of aging have been defined: genomic instability, telomere attrition, epigenetic alterations, loss of proteostasis, disabled macroautophagy, dysregulated nutrient-sensing, mitochondrial dysfunction, cellular senescence, stem cell exhaustion, altered intercellular communication, chronic inflammation, and dysbiosis [415]. Cellular senescence, i.e., the aging of a particular cell type, is one hallmark of aging and has been categorized as an antagonistic mechanism. Thus, as long as senescent cells are removed, this mechanism mainly promotes rejuvenation of tissues and tissue repair. On the other hand, “resisting cell death” is a well-known hallmark of cancer [416]. As a consequence, eryptosis and erythrocyte senescence may be two distinct mechanisms. The main difference between eryptosis and senescence of RBCs is the time constant of clearance, and it was shown that ionomycin-treated eryptotic mouse cells are cleared within minutes, whereas the clearance process of normal, healthy mouse erythrocytes takes several days [417]. Nevertheless, it is possible that the two processes influence one another. Indeed, Ghashghaeinia et al. showed that old erythrocytes (this means senescent erythrocytes) are more susceptible to oxidation-induced eryptosis [72]. However, there remains one important question: Is there a direct connection between the age of the individual person and the corresponding erythrocyte blood parameters? In a recent paper, it was shown that RBCs can serve as biomarkers of the aging process: important RBC indices, e.g., the erythrocyte hemoglobin concentration, the mean corpuscular volume (MCV), and erythrocyte metabolism change with the age of the individuals [418]. However, future research is needed to unravel the biological mechanisms underlying this clinical observation.

## Induction of eryptosis by environmental pollutants

When assessing the harm caused by environmental pollutants and their potential toxicological risk, a number of parameters should be taken into consideration. One such factor is the evaluation of eryptosis induction, as this can indicate faster removal of erythrocytes from the circulation after exposure to pollutants. Such analyses can be performed in epidemiological studies, such as in workers exposed to lead [302, 419], or in in vitro systems which can determine the molecular basis of apoptotic effects [420–423]. Hence, numerous studies have examined the eryptotic properties of various environmental pollutants and the molecular basis of eryptosis; such data may also explain the mechanism of poisoning caused by specific pollutants. Recent years have seen a number of studies examining the eryptotic properties of a range of environmental pollutants, including heavy metals and organic compounds such as bisphenols, flame retardants, phthalates, and pesticides.

### Eryptosis and occupational exposure to lead

An epidemiological study of male workers exposed to lead (64.8 µg lead/dL blood) and workers not exposed to lead (4.2 µg lead/dL blood) revealed a poisoning, characterized by 88.3% lower δ-aminolevulinic acid dehydratase (δALAD) activity. Exposure also resulted in eryptosis induction, probably due to the prooxidative effect of lead (2.4-fold higher levels of thiobarbituric acid reactive substances (TBARS) and 32.8% lower GSH/GSSG ratio). Exposure to lead resulted in elevated intracellular Ca^2+^ level, activation of μ-calpain, and ultimately externalization of PS in erythrocytes: the exposed workers showed 2.82% PS externalization compared to 0.1% for unexposed controls [302].

Hernández et al. assessed the involvement of PLA_2_ and SMase in the molecular pathways leading to oxidative stress-induced eryptosis in workers exposed to lead. They studied 30 workers exposed to lead and a group of 27 unexposed controls. The results indicate that lead poisoning was characterized by high blood lead concentration and low δ-ALAD activity, induction of oxidative stress with high lipid peroxidation and low total antioxidant capacity (TAC), as well as a higher degree of PS externalization. Additionally, the exposed group demonstrated significantly higher PLA_2_ and SMase enzyme activity, with their median values being 518 and 706 AFU/mg, compared to 267 and 444 AFU/mg in the unexposed workers. Statistical analysis found PLA2 activity to be a more effective mediator between oxidative stress and eryptosis than SMase activity; it is most likely that the PLA_2_/PGE2 pathway has a more significant role in eryptosis [419]. The lead-induced eryptosis observed in epidemiological studies has also been confirmed by in vitro research [424]; treatment of blood cells with lead resulted in ROS-independent eryptosis without any increase in ROS, but with an increase in Ca^2+^ level and caspase-3 activity.

### Eryptosis and its potential use in toxicological assessment of heavy metals

One group of pollutants that pose a particular threat to human health are the heavy metals, due to their toxicity, longevity in the atmosphere, and ability to bioaccumulate in the human body. Although their effects are not immediate, when taken in small doses, they often become apparent after many years or generations of exposure, and remain poorly understood [425]. As such, the mechanism underpinning the induction of eryptosis by heavy metals is of considerable interest, and it is under investigation in various studies.

Heavy metals inducing eryptosis include aluminum. In vivo and in vitro tests have linked the effects of aluminum exposure to anemia. To better understand the mechanisms of action of aluminum on human erythrocytes, an in vitro study examined the morphological and biochemical changes occurring after long-term treatment of erythrocytes. It was shown that aluminum exposure altered cell morphology, which suggested that the metal attached to the cell membrane and interacted with the cell surface. Long-term incubation of human erythrocytes with aluminum induced PS externalization, increased intracellular Ca^2+^ levels, and degradation of AE1 proteins, which are markers of eryptosis. Hence, chronic exposure to aluminum may lead to biochemical and morphological changes characteristic of eryptosis. Interestingly, erythropoietin exerted an antieryptotic effect, which protected against the prooxidant effect of aluminum [426].

Eryptotic effects have also been attributed to nickel. Alfhili et al. showed that exposure to nickel chloride in vitro induced lytic cell death and eryptosis, which is mediated by Ca^2+^ influx and p38 MAPK signaling. Briefly, the cells were incubated for 24 h with NiCl_2_ in the concentration range of 0.5–10 mM. At a concentration of 10 mM, exposure was found to cause profound intracellular Ca^2+^ overload and significant Ca^2+^-dependent lytic cell death. It also changed the structure of the cell membrane (SSC, side scatter) and cell size (FSC, forward scatter), increased the level of ROS and fluorescence of FITC conjugated with annexin V. It was shown that 10 mM NiCl_2_ caused a significant increase in PS externalization (1.51 ± 0.2% vs. 48.0 ± 10.58%, *p* < 0.0001). It was therefore concluded that NiCl_2_ induces p38 MAPK-dependent lytic cell death and stimulates markers of premature eryptosis, which may explain the changes in hematological parameters observed in poisoning victims [420].

Other studies have examined the effect of hexavalent chromium on regulated RBC death. It was shown that 48-h exposure to chromium (VI) (≥10 μM) significantly increased the concentration of cytosolic Ca^2+^ level, decreased the concentration of ATP (20 μM), or increased the binding of annexin V. Chromium (VI) did not significantly modify the formation of ceramides. The effect of 20 μM chromium (VI) on annexin V binding was partially reversed when Ca^2+^ was removed. Ca^2+^ influx and cytosolic ATP depletion in erythrocytes resulted in eryptosis, with cell shrinkage and the mixing of cell membrane components [427].

Previous hexavalent chromium toxicity studies also found exposure to 20 µM chromium (VI) to result in eryptosis, together with Ca^2+^ influx, increased ROS levels, and rapid ATP consumption. It was found that, as in the case of other xenobiotics, Ca^2+^ influx plays an extremely important role in chromium (VI)-dependent toxicity [184].

### Eryptosis may be used to evaluate the substitutes for existing compounds

Bisphenols and retardants are regarded as significant “environmental toxins”, as they are widely present and can enter human bodies. Recent years have seen a number of studies of the eryptotic properties of many compounds from these groups.

Eryptosis evaluations may play a role in the toxicological characterization of new substitutes for existing compounds. For example, due to numerous reports of the potential toxicity of tetrabromobisphenol A (TBBPA), there is growing interest in the use of alternative compounds such as tetrabromobisphenol S (TBBPS). As a result, previous studies have examined the eryptotic properties of bisphenol A (BPA) and its substitute bisphenol S (BPS) [422], brominated flame retardants (BFRs) [421], and organophosphate flame retardants (OPFRs) [428].

Bisphenols are widely used in the production of polycarbonates, epoxy resins, and thermal paper, and their presence is commonly recorded in both the environment and living organisms [429]. Maćczak et al. assessed eryptotic changes in human erythrocytes exposed for 24 h in vitro to BPA and its selected analogs, i.e., bisphenol F (BPF), BPS, and bisphenol AF (BPAF). They found that all analyzed bisphenols, particularly BPAF and BPF, increased PS externalization. These compounds also caused a significant increase in the activity of calpain and caspase-3, with the strongest effect observed in the case of BPAF. Eryptosis is stimulated by concentrations observed in the human body during occupational exposure or subacute poisoning. No changes were observed at low concentrations that would be related to environmental exposure. Importantly, BPS, which is the main substitute for BPA in polymers, demonstrated similar eryptotic potential to BPA, indicating in this area of research that it is not a suitable replacement for BPA [422].

BFRs are also of concern due to their widespread occurrence in the environment and adverse effects on living organisms, including humans. BFRs are synthetic flame retardants used in everyday products such as electrical and electronic devices, textiles, and furniture. Studies have documented the presence of BFRs in the environment, food, drinking water, inhaled dust, and the human body [429, 430]. Jarosiewicz et al. assessed the eryptotic properties of selected BFRs in human erythrocytes: TBBPA and TBBPS, and the bromophenols 2,4-dibromophenol (2,4-DBP), 2,4,6-tribromophenol (2,4,6-TBP), and pentabromophenol (PBP). All tested compounds were found to cause eryptosis by externalizing PS and activating caspase-3; in addition, very low concentrations (0.001–0.01 µg/ml) corresponding to environmental exposure also increased ROS production, which induced eryptosis due to oxidative stress. Moreover, it was shown that calcium ions and calpain do not play a significant role in the induction of eryptosis by BFR. The greatest eryptotic and oxidative changes were caused by TBBPA, while the lowest were associated with its substitute, TBBPS, which highlights the validity of replacing TBBPA with TBBPS [421].

Other in vitro studies on human erythrocytes have assessed the induction of eryptosis by OPFRs such as tris(2-chloroethyl) phosphate (TCEP) and tris(1-chloro-2-propyl) phosphate (TCPP). OPFRs are widespread in air, water, and food, and their ingestion results in their presence in human blood and urine [431, 432]. Erythrocytes were treated with OPFR for 24 h at concentrations ranging from 0.001 to 1,000 g/mL. It was found that the OPFRs only increased the level of ROS and methemoglobin at very high concentrations: at 500 µg/mL, they were found to induce morphological changes and ultimately cause lytic cell death and PS externalization. TCPP showed stronger oxidative and eryptotic potential than TCEP. Generally, OPFRs [428] exhibited much lower toxicity than BFRs [421, 422] towards human erythrocytes.

### The use of eryptosis in the assessment of toxic parent compounds and their metabolites

Eryptosis studies can also be used to compare parent compounds with their metabolites. Phthalates are widely used as plasticizers in various fields, including food, cosmetics, and pharmaceuticals. These compounds do not form covalent bonds with the substances to which they are added, so they can easily migrate and penetrate various everyday products. Significant amounts of phthalates and their metabolites are found in urine, breast milk, blood serum, venous blood, and umbilical cord blood. Phthalates have endocrine-disrupting properties and are harmful to human health [433]. Sicińska assessed the level of lytic cell death and eryptosis in vitro caused by the phthalates di-n-butyl phthalate (DBP) and butylbenzyl phthalate (BBP) and their metabolites: mono-n-butyl phthalate (MBP) and monobenzyl phthalate (MBzP). The compounds were incubated with human RBCs for 24 h at concentrations from 0.5 to 500 µg/mL. It was found that DBP and BBP demonstrated stronger hemolytic properties than their metabolites. All compounds induced eryptosis, causing translocation of PS, increased levels of cytosolic calcium ions, and increased activation of caspase-3 and calpain in the human erythrocytes. While BBP caused PS translocation at a lower concentration than DBP, it was generally found that DBP had a greater influence on the other tested parameters at lower concentrations. The metabolites showed much lower toxicity compared to the parent compounds [423].

### Eryptosis and its use in the estimation of pesticide toxicity

Another toxic chemical is rotenone. It is found in a variety of plants, including some edible ones. Rotenone poisoning can be fatal, and there is no antidote. It acts by inhibiting mitochondrial complex I, which reduces ATP production, which results in compensatory upregulation of glycolysis, secondary lactate production, and oxidative stress [434]. Due to its toxicity, the compound is used as an insecticide, which acts by stimulating apoptosis in nucleated cells. Furthermore, rotenone poisoning has been implicated as a cause of Parkinson’s disease. One study examined whether rotenone causes eryptosis. It was found that 48-h exposure to rotenone significantly increased Ca^2+^ content, indicated by Fluo3(i) fluorescence, and increased ceramide content, decreased cell size measured as FSC, and increased annexin V binding. Minor lytic cell death also occurred following exposure. Thus, rotenone was confirmed to possess eryptotic effects [435].

Another widespread pollutant is N,N-diethyl-3-methylbenzamide (DEET), the most widely used insect repellent in the world. Its adverse effects are well documented, and DEET has been shown to have cytotoxic and apoptotic properties in nucleated cells. Six-hour incubation of human RBCs with 1–5 mM DEET resulted in slight lytic cell death at concentrations of 4 and 5 mM and significantly increased Annexin V FITC and Fluo3 fluorescence, with reduced FSC observed at 5 mM [436]. Removal of extracellular Ca^2+^ abolished DEET-induced Fluo3 fluorescence but had no effect on annexin V binding. Thus, DEET induces erythrocyte cell death; an event characterized by loss of cell membrane asymmetry, cell shrinkage, and an increase in intracellular Ca^2+^, primarily due to dysregulated Ca^2+^ influx [436]. Another organophosphate insecticide is bromfenvinphos: (E,Z)-O,O-diethyl-O-[1-(2,4-dichlorophenyl)-2-bromovinyl] phosphate. The compound reduces hematocrit and hemoglobin levels, probably by inducing oxidative stress in erythrocytes. Forty-eight hour exposure of human erythrocytes to bromfenvinphos (≥100 µM) resulted in a significant increase in the percentage of cells binding annexin V, a significant decrease in FSC, a significant increase in Fluo3 fluorescence, and a significant increase in 2′,7′-dichlorofluorescein (DCF) fluorescence; this indicates that bromfenvinfos caused cell shrinkage and phospholipid mixing in the erythrocyte cell membrane, which is partly due to stimulation of ROS formation and Ca^2+^ entry [437].

Hence, it can be seen that many environmental pollutants are capable of inducing eryptosis in human RBCs, and that eryptosis may be an effective indicator for comparing the hematotoxicity of parent compounds and their metabolites [423], to assess the toxicity of their potential replacements [421, 422, 428], and to investigate the mechanisms of action of toxic metals [184, 420, 424, 435] and pesticides [436, 437]. Eryptosis-based assessment may also be used to assess occupational exposure and the potential toxic effects of chemicals occurring in the work environment [302, 419].

### Eryptosis and cigarette smoke

Cigarette smoking is well-established as a significant contributor to oxidative stress and systemic inflammation, both of which are key drivers of various pathological conditions, including cardiovascular and pulmonary diseases [232]. Eryptosis is induced by oxidative stress [136] and has been increasingly linked to chronic vascular inflammatory disorders such as atherosclerosis [438, 439]. While the impact of cigarette smoking on the redox balance of several tissues has been extensively studied, its specific role in triggering oxidative injury in erythrocytes has only recently gained attention. In a 2019 in vivo study, Attanzio et al. have reported in healthy male smokers (*n* = 21) compared to non-smokers (*n* = 21) a significant increase in externalization of PS to the outer leaflet of the RBCs membrane, a hallmark of eryptosis, which was significantly correlated to the plasma level of CRP and GSH concentration in erythrocytes [440]. In 2024, a study by Schmitt et al., involving a significantly larger cohort of 2023 participants, confirmed that smokers (*n* = 418) exhibited higher levels of circulating eryptotic erythrocytes compared to non-smokers (*n* = 1000) and ex-smokers (*n* = 605) [441]. Moreover, the level of eryptotic cells was positively associated with the number of cigarettes smoked daily.

Restivo et al. have recently explored the molecular mechanism through which CSE triggers eryptosis. This study provides evidence that exposure of isolated fresh erythrocytes for 24 h to CSE induces eryptosis by p38 MAPK-initiated DISC formation, followed by activation of caspase-8/caspase-3 via ceramide formation by neutral SMase. This mechanism was confirmed by the evidence that erythrocytes isolated from smokers exhibited higher levels of membrane-recruited caspase-8 and FADD than non-smokers. However, a limitation of that study was that the number of subjects considered was very small [130].

Given the harmful effects of eryptotic RBCs on endothelial cells and platelets, which can cause vascular inflammation and blood coagulation, these studies provide a rationale that links cigarette smoke to vaso-occlusive complications and atherogenesis.

### Eryptosis as an approach to test hemocompatibility of nanomaterials

Nanomedicine is a fast-growing branch of nanotechnology focusing on the application of nanoscale materials for monitoring, diagnosing, preventing, and treating diseases [442]. Owing to their small size (1-100 nm) and high surface-to-volume ratio, nanomaterials possess specific properties determining unique nano-bio interactions. Recent decades have witnessed a tremendous increase in the number of studies investigating the possible application of nanotherapeutics, especially in the field of cancer [443]. Currently, more than 15 anti-cancer nanomedicines, including lipid-based NPs, protein-drug conjugates, and metallic NPs, have been granted approval status by the U.S. Food and Drug Administration (FDA) or the European Medicines Agency (EMA) [444]. Furthermore, there is a clear ascending trend in the number of clinical trials in cancer nanomedicine [445]. However, the successful clinical translation of nanomedicine is challenging primarily due to safety concerns [446]. In this regard, a smaller size and a higher surface area of nanomedicines act as a double-edged sword, determining the high reactivity of nanomaterials, which can contribute both to effectiveness and toxicity.

The field of nanotoxicology lacks standardized guidelines, and the current efforts aim to adapt OECD Test Guidelines for Chemicals to be used for nanotoxicological properties [447]. Novel guidelines for testing nanomaterials have been developed within the Malta Initiative since 2017, and the launch of EU-funded projects, including the Horizon 2020-funded NanoHarmony, RiskGONE (Science-based Risk Governance of NanoTechnology), NANORIGO (NANOtechnology RIsk Governance), or Gov4Nano. Evaluation of hemocompatibility is an important issue in the overall assessment of the safety of nanomedicines, since the administered nanoscale materials enter the bloodstream and interact with blood plasma proteins, erythrocytes, leukocytes, platelets, and endothelial cells. To assess the nanotoxicity in a complex way, all-inclusive hemocompatibility testing, which includes assessment of hematocrit, hemostasis, nano-cell interactions, immunotoxic effects, etc., is recommended [448].

Hemolysis is a parameter of fundamental importance in hemocompatibility testing [448, 449]. However, eryptosis has been suggested to have certain advantages for hemocompatibility testing and can be used to supplement data of hemolysis assays [450]. For instance, since eryptosis physiologically precedes hemolysis, the sensitivity of eryptosis assays is higher than that of hemolysis. Additionally, eryptosis offers a higher reproducibility, easier quality control, better data precision, and the opportunity to identify changes in eryptosis signaling pathways at the suberyptotic concentrations of nanomaterials. Furthermore, hemolysis as an ACD is non-specific and reflects only the mechanistic damage to cells. In turn, eryptosis assays can be used to identify specific signaling pathways triggered by nanomaterials, which might shed light on their possible therapeutic application during in vitro toxicity testing. However, implementation of eryptosis assays in nanotoxicology can be limited by the lack of a generally accepted single eryptosis marker and the requirements to use several markers simultaneously, their lower cost-effectiveness compared to the hemolysis assays, and the availability of the equipment, since eryptosis detection is primarily flow cytometry-based.

**Fig. 6:**
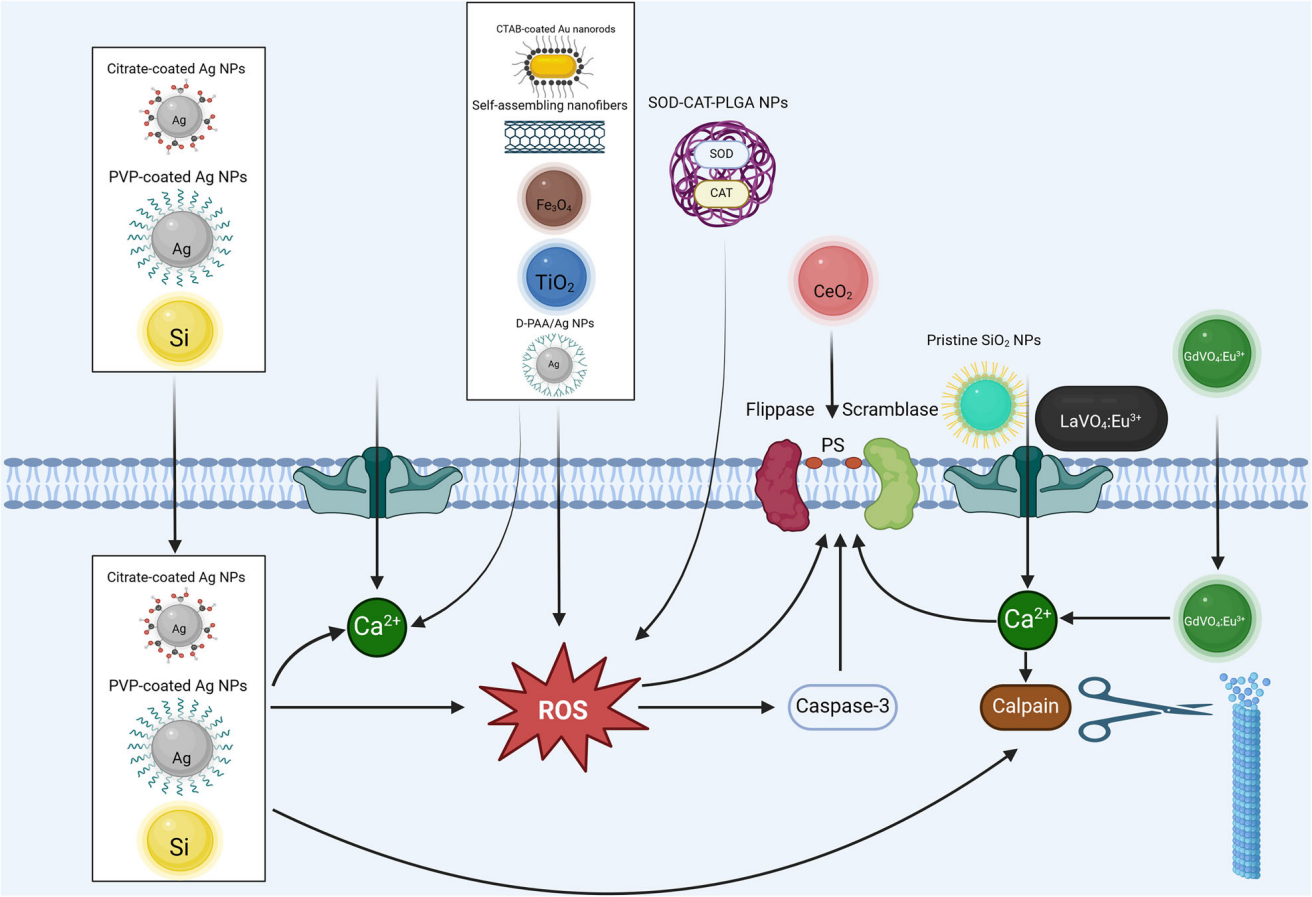
Multiple nanomaterials trigger Ca^2+^-dependent eryptosis primarily via ROS-mediated signaling. Eryptosis can be induced by both internalized and non-internalized nanomaterials. Nanomaterial-induced eryptosis might be mediated by caspase-3 and calpain. CAT catalase, CTAB cetyltrimethylammonium bromide, D-PAA dextran-polyacrylamide, NPs nanoparticles, PLGA poly(lactic-co-glycolic acid), PS phosphatidylserine, PVP polyvinylpyrrolidone, ROS reactive oxygen species, SOD superoxide dismutase. Created with Biorender.com.

A growing body of evidence suggests that nanoscale materials can trigger Ca^2+^-dependent eryptosis in mature erythrocytes obtained from various species in a time- and dose-dependent fashion [450]. Features of nanomaterial-induced eryptosis are summarized in Fig. 6. Importantly, nanomaterial-induced cytotoxicity is frequently mediated by ROS [451]. Indeed, oxidative stress associated with excessive ROS production and depletion of the antioxidant system is commonly observed in nanomaterial-induced eryptosis [452–457]. Additionally, eryptosis triggered by nanoscale materials can be associated with caspase-3 activation [452, 454] and calpain involvement [453, 458]. It is important to note that internalized nanoscale materials, including citrate-coated Ag NPs [453], polyvinylpyrrolidone-coated Ag NPs [453], and Si NPs [458], trigger eryptosis in a Ca^2+^-, ROS-, and calpain-mediated fashion (Fig. 6). Uptake of GdVO_4_:Eu^3+^ NPs triggers Ca^2+^-mediated eryptosis [459]. Ca^2+^ signaling and/or ROS have been shown to contribute to eryptosis induced by cetyltrimethylammonium bromide-coated Au nanorods [454], self-assembling antimicrobial nanofibers [457], Fe_3_O_4_ NPs [456], TiO_2_ NPs [455], SOD-CAT-poly(lactic-co-glycolic acid) NPs [460], and dextran-polyacrylamide-embedded Ag NPs [461]. However, their internalization was not investigated. Notably, non-internalized NPs like pristine SiO_2_ NPs [452] and LaVO_4_:Eu^3+^ NPs [459] can still trigger eryptosis, relying on Ca^2+^ signaling, which can be mediated by their direct influence on Piezo1 channels. Furthermore, pristine SiO_2_ NPs, which could not be taken up by erythrocytes, were shown to promote ROS-mediated and caspase-3-dependent eryptosis [452] (Fig. 6). To our knowledge, there are no reports on lipid-mediated and extrinsic eryptosis triggered by nanomaterials. At the moment, the features of nanomaterial-induced eryptosis signaling are poorly elucidated due to the lack of studies. Furthermore, identification of the physical and chemical factors that affect the erythrotoxicity of nanomaterials and their ability to trigger eryptosis is of huge importance. At the moment, there is some evidence that CeO_2_ NPs of lower size are more potent inducers of eryptosis than bigger CeO_2_ NPs [462]. Shape can affect internalization of nanomaterials, which influences their ability to trigger apoptosis [459]. There is accumulating evidence that coating might reduce eryptosis-inducing properties of nanomaterials [450, 452, 457], which can be exploited to engineer NPs with a better hemocompatibility profile.

Thus, eryptosis markers in the nanotoxicological field are effective and sensitive hemocompatibility parameters that allow investigating nanomaterial-induced signaling cascades.

## Eryptosis assays in toxicology: is it a good alternative to hemolysis assays?

Translation of biomaterials from bench to bedside requires a thoughtful analysis of their toxicological profile [463]. Among multiple safety parameters that have to be assessed, hemocompatibility is among the essential criteria. In particular, hemocompatibility of blood-contacting devices is tested in vitro in accordance with the ISO standard 10993-4 guidelines, and this testing comprises evaluation of thrombosis, the blood coagulation system, the complement system, and multiple effects on platelets and leukocytes [464]. On top of that, interactions of biomaterials with erythrocytes, which are the most abundant cells in circulation, are mainly evaluated by in vitro hemolysis testing [465, 466]. However, hemolysis, which can be considered as necrosis of erythrocytes, belongs to the so-called ACD modalities, indicating that this cell death occurs in response to physical or mechanical damage to RBCs and lacks signaling pathways [4]. Thus, hemolysis reflects just the mechanically induced injury to erythrocytes and does not allow for determining specific signal transduction modules involved in cell death induction and execution. This disadvantage can be overcome by applying eryptosis for hemocompatibility in vitro testing.

In a recently published review, the advantages of eryptosis over hemolysis as a toxicological endpoint have been summarized, emphasizing that eryptosis in vitro testing has a higher sensitivity, is more accurate and reproducible, provides better quality control, and can shed light on the cytotoxicity-mediating molecular mechanisms compared with hemolysis [450]. Intuitively, eryptosis as a protective mechanism aiming at eliminating aged or injured RBCs from circulation [89] should precede hemolysis and be triggered by sub-hemolytic stress stimuli. Indeed, over 110 different compounds, including metals, their oxides, metal salts, drugs, kinase inhibitors, endogenous toxins, inhibitors of signaling pathways, alkaloids and other plant-derived biologically active substances, have been shown to induce eryptosis in vitro at lower concentrations than hemolysis or to a higher extent at the same concentrations [450]. Moreover, application of the eryptosis assay for hemocompatibility in vitro testing can be helpful for identifying potential pharmaceutical agents that can target eryptosis, which can be exploitable for treating diseases associated with either accelerated or inefficient eryptosis [467].

## How to study eryptosis?

With the growing interest in RBC pathology and the appreciation of the role of RBC death and survival in a plethora of conditions, standardized guidelines for the experimental demonstration of the various forms of cell death known to exist in RBCs are mandatory. The following discussion offers an experimental framework for the detection of RBC death through various toxic endpoints.

### Blood samples

- Research on human subjects or specimens collected from humans must comply with institutional ethical regulations. In all cases, approval of the local institutional review board is required. Authors are legally required to adhere to the Declaration of Helsinki when interacting with donors and to explicitly mention the name of the granting body and approval date, and number.
- Anticoagulants – EDTA [256, 468], sodium citrate [413], and heparin [469, 470] are acceptable. If running a complete blood count (CBC) is desired, EDTA samples are the most suitable.
- Exclusion criteria – subjects with conditions that have so far been identified as modulators of RBC survival must be excluded from participation [89].
- Since this in vitro investigation is a descriptive study, no sample size calculation is required [471]. However, at least three independent experiments, each containing two or three technical replicates, must be performed for each parameter. This brings the total number of readings for each treatment condition to 6–9. In very few cases (e.g., enzyme-linked immunosorbent assay, ELISA; Western blotting, WB, etc.), and depending on the analyte assayed, pooling of samples may be economically more appropriate.
- For in vivo studies, the number of animals used must be kept to an absolute minimum [472]. In case the study is on humans, the sample size must be calculated to achieve a statistical power of at least 80% [473].
- PS exposure is not affected by body mass index (BMI), age, gender, or ABO/Rh blood group [474]. However, prioritizing non-pregnant subjects outside the extremes of age decreases the likelihood of unforeseen confounding variables. Fasting is not required.

### RBC isolation

- A pure RBC suspension can reliably be achieved by density-gradient centrifugation (2500 RPM, 15 min, RT). Although not required, detection of glycophorin A (CD235a) and/or the absence of CD45 confirms the purity of RBC preparations [475].
- Final hematocrit upon stressor exposure – authors have used a wide range from 0.4% to 30% [422, 476–478]. It has been shown that lower densities prime cells for hemolysis and eryptosis [398]. As a rule, too few cells are more sensitive, while too many may mask potential toxicity.

### Buffers and solutions (Table S1)

- Ringer solution.
- Modified Ringer solutions.
- Combined Ringer solutions.
- Addition of antimicrobial agents:
  i. 100X Anti-Anti mixture – this is commercially available in different formulations, but a common preparation includes 10,000 U/mL penicillin, 10 mg/mL streptomycin, and 25 μg/mL amphotericin.
  ii. Penicillin (1000 U/L), streptomycin (0.1 g/L), and amphotericin B (0.25 mg/L).
- A previous study compared the effect of standard Ringer buffer with that of RPMI on the susceptibility of cells to hemolysis and eryptosis and concluded that Ringer buffer is a nutrient-poor medium while RPMI is nutrient-rich [398]. Further examination of the effect of incubation media is warranted.

### Canonical markers of hemolysis

- Hb in the supernatant can reliably be measured at 405 nm [479].
- Supernatants may be isolated by centrifugation at appropriate speed and duration (e.g., 13,000 rpm for 1 min, 500 rpm for 5 min, etc.).
- Drabkin’s reagent reacts with all Hb species except sulfhemoglobin.
- Release of K^+^, lactate dehydrogenase (LDH), and aspartate aminotransferase (AST) (colorimetric, manual, or automated).
- Although RBCs lack creatine kinase (CK), metabolites in the cytosol (ADP and glucose-6-phosphate) or enzymes in the cell membrane (adenylate kinase; AK) interfere with the assay reactions for CK, thereby falsely increasing activity values. In particular, they produce ATP, which leads to falsely high results when AK inhibitors in the CK reaction are exhausted. Therefore, CK activity can be used as a surrogate marker for the leakage of these cellular metabolites, which confirms membrane rupture.
- In all cases, using equal volumes of supernatants when detecting optical density (OD) is key for accurate comparison.

### Canonical markers of eryptosis

- PS exposure – fluorescein-conjugated annexin V by flow cytometry (FCM) (gold standard) or fluorescence microscopy; ELISA kits have also been developed [480].
- Aminophospholipid translocase – reverses the orientation of externalized PS; activity is measured by a fatty acid-labeled phosphatidylserine (NBD-PS) fluorescent probe [136].
- Cell shrinkage or swelling – FSC (gold standard) or MCV by an automated hematology analyzer
- Surface granularity – SSC or microscopy.
- Cellular morphology – light, phase-contrast, or electron microscopy
- Intracellular calcium – Fluo4/AM or X-Rhod-1, AM [398].
- Oxidative stress:
  i. ROS – DCF (H2DCFDA staining).
  ii. Specific ROS (colorimetric or fluorogenic substrate-based methods):
    1. Hydrogen peroxide (H_2_O_2_) – Abcam #ab138874; Amplex Red.
    2. Superoxide anion (O_2_^-^) – Abcam #ab139477 (FCM).
    3. Hydroxyl radicals (OH^-^) – Abcam #ab219931 (microscopy).
    4. Singlet oxygen (^1^O_2_) – Thermo #S36002 (in lysates – fluorometry).
    5. HO, HOCl, and NOO – aminophenyl fluorescein (Thermo #A36003).
    6. HO and NOO – hydroxyphenyl fluorescein (Thermo #H36004).
  iii. Lipid peroxides – BODIPY dyes or colorimetric kits.
  iv. Protein carbonylation – colorimetric kits.
  v. Protein sulfenylation – colorimetric kits.
  vi. Protein damage – tryptophan fluorescence (decrease in OD).
  vii. 3-nitrotyrosine – Abcam #ab116691.
  viii. Antioxidant enzymes (CAT, SOD) – colorimetric/ELISA/FCM kits.
  ix. Glutathione system (GSH, GSSG, GPx, glutathione S-transferase (GST), GR) – colorimetric kits/FCM (mercury orange 1(4-chloromercuryphenyl-azo-2-naphthol; Ex/Em = 488/576 nm)) [481].
  x. Total thiols – 5-Chloromethylfluorescein diacetate (5-CMFDA) [482].
  xi. TAC – colorimetric kits.
- μ-Calpain – increased [422, 423, 483] and decreased [421] activities have been reported – CMAC (t-butyloxycarbonyl-Leu-Met conjugated to chloromethylcoumarin); Thermo #A6520.
- Nitric oxide:
  i. DAF-FM diacetate for FCM.
  ii. ii. Colorimetric.
- Energetics:
  i. Glucose uptake - colorimetric (Abcam #ab136955).
  ii. Glycolysis –HK, phosphofructokinase-1 (PFK-1), and pyruvate kinase (PK) are the rate-limiting enzymes.
  iii. PPP –G6PD, transaldolase, and transketolase are the rate-limiting enzymes [484]
  iv. Luebering–Rapoport pathway – 2,3-DPG.
  v. ATP and ADP – ATP depletion [485] and accumulation [486] have been reported – colorimetric or chemiluminescent kits.
  vi. NAD^+^ and NADH.
  vii. NADP^+^ and NADPH.
  viii. Lactate [487].
  ix. Intracellular pH – Cayman #15922.
  x. Na^+^/K^+^-ATPase activity – colorimetric kits.
- B_12_ and folic acid – colorimetric kits, manual or automated.
- Membrane potential by DiOC2(3) (3,3’-Diethyloxacarbocyanine Iodide).
- Detection of changes in membrane proteins by electrophoresis – proteins are readily identifiable based on their respective size; WB is also possible.
- Senescence – β-galactosidase [488] by fluorogenic probes.
- Intracellular iron – Phen Green SK diacetate.
- Cellular aggregation – ESR (Westergren tubes or automated).

### Canonical markers of necroptosis

- Detection of necroptosome assembly by WB or ELISA [25, 27, 82].
  i. p-RIPK1.
  ii. p-RIPK3.
  iii. p-MLKL.
- Detection of Syk and Src phosphorylation by WB or ELISA.
- Inhibition of cell death by blocking RIPK1 (e.g., Nec-2).
- Inhibition of cell death by blocking RIPK3 (e.g., GSK’872 or HS-1731.)
- Inhibition of cell death by blocking MLKL (e.g., NSA).
- Inhibition of cell death by blocking Syk kinase (e.g., BAY 61-3606).
- Inhibition of cell death by blocking Src kinase (e.g., PPI).
- Lack of inhibition of cell death by blocking caspases (e.g., Z-VAD-FMK).

### Signaling

- Since genetic manipulations (e.g., knockdown, knockout, overexpression, etc.) are not possible in RBCs, pharmacological modulation of surface and intracellular proteins by specific antibodies, inhibitors, and agonists remains the only currently available approach. A wide assortment of modulators has been used and validated; some of the most common are presented in Table S2.

Inhibitor concentration and pre- and co-treatment protocols must be empirically determined based on cell density, duration of exposure, and unique drug-drug interactions.

Many other inhibitors/agonists may also be used.

Small molecule inhibitors as tools to scan for active pathways do not conclusively rule out the participation of a signaling mediator if a negative result is obtained (i.e., no inhibition/augmentation of a toxic endpoint). The pathway may still be active, but may nonetheless not be necessary for the endpoint being investigated. Many inhibitors also have off-target effects, which become more pronounced at high concentrations. A more comprehensive approach would be to concurrently measure actual protein levels (e.g., WB, ELISA, etc.) of potential targets in addition to inhibitor assays.

### Inhibition of cell death

To investigate the potential protective effects of a given compound against RBC death, experiments must be designed to subject the cells to different physical and chemical stressors with and without the compound of interest, and multiple toxic endpoints are then examined. The experimental framework through which studies may reliably assess the ameliorative potential of the tested compound is available in Table S3.

## Is eryptosis therapeutically exploitable?

### Which mechanisms or molecules can be targeted in eryptosis signaling pathways?

Eryptosis, analogous to apoptosis in nucleated cells, is a physiological process that eliminates senescent or defective erythrocytes before hemolysis occurs, thereby preventing toxicity resulting from the release of hemoglobin into the bloodstream. This process plays a crucial role in maintaining the balance between erythropoiesis and RBC destruction. Inhibitors of eryptosis could potentially be exploited for therapeutic purposes, particularly in conditions where excessive or insufficient erythrocyte removal contributes to disease progression [134]. Stimulators of eryptosis may be applicable in malaria to accelerate eryptosis and thus trigger the removal of infected erythrocytes [211].

Modulating eryptosis, whether by inducing or inhibiting the process, could help regulate erythrocyte survival to prevent premature cell loss or promote the removal of damaged erythrocytes, thereby contributing to the proper balance of blood cell counts. In this context, it has been reviewed that certain substances can stimulate tumor cell apoptosis and, by the same token, inhibit eryptosis, which may avoid side effects such as anemia found in cancer patients subjected to chemotherapy. For instance, those dual effects are triggered by teriflunomide, which may thus prove useful in the treatment of anemia in patients with malignancy [489]. On the other hand, β-cryptoxanthin (or other eryptosis inductors) exhibited proeryptotic and hemolytic effects, a fact that could accelerate the death of erythrocytes infected by plasmodium and help fight malaria [490].

For this purpose, the adjunctive therapeutic use of small molecules that either inhibit or stimulate eryptosis could represent a valid strategy for the treatment or prevention of pathological complications associated with erythrocyte dysfunction. Further experimental investigations are needed to determine whether the beneficial effects of any small molecules that stimulate or inhibit erythrocyte apoptosis are effective in the ex vivo or in vivo treatment of anemia, malaria, or systemic pathologies associated with eryptosis [211]. Likewise, it has been recently reported that more research is needed in this field to evaluate the real role of eryptosis as a biomarker of cardiovascular risk prognosis [491].

The main molecular mechanisms involved in the modulation of eryptosis studied to date, including intracellular calcium levels, ceramide, ROS, NO, changes in cellular energy metabolism, PS externalization, ion channel regulation, Fas-mediated signaling pathways, kinase-associated, and caspases activation, among others represent potential targets for inducing or inhibiting erythrocyte death.

Understanding these targets, the mechanisms of apoptotic signaling pathways, and identifying additional factors that trigger or inhibit the apoptotic process could represent potential therapeutic strategies to enhance erythrocyte health and overall human well-being.

### Eryptosis induction

Excessive eryptosis is associated with a broad range of diseases and is triggered by various endogenous and exogenous molecules.

Table S4 presents a list of articles from Lang’s research group, as well as some recent studies, on substances that can trigger eryptosis both ex vivo and in vivo.

In particular, inhibitors or activators targeting cellular components, as well as biomolecules, enzymes, and nutrients, can stimulate apoptotic signaling pathways. Furthermore, natural compounds and phytochemicals used to counteract or prevent diseases can lead to premature erythrocyte death. Many drugs, including antibiotics, anti-inflammatories, chemotherapeutics, antipsychotics, and others, have demonstrated cytotoxic effects on erythrocytes. Nanomaterials, along with toxins, mycotoxins, and ionophores listed in Table S4, induce cytotoxicity leading to apoptosis of erythrocytes. Additionally, exposure of erythrocytes to heavy metals, pollutants, and xenobiotics triggers eryptosis through various mechanisms of cell death.

The compounds listed in Table S4, tested at various times and at concentrations with statistically significant effect, induce eryptosis dependent on calcium, ceramide, and ROS. Furthermore, for specific compounds, additional key mechanisms such as Fas-mediated signal transduction, loss of cellular redox homeostasis, ATP depletion, changes in ion channels, and activation of kinases and caspases, among others, have been highlighted.

Given the experimental evidence mentioned above, investigating new proeryptotic and antieryptotic molecules is essential for expanding our understanding of the cellular mechanisms underlying the programmed death of erythrocytes and for opening new avenues in the study of pathologies associated with eryptosis. In this regard, the implementation of in vivo animal studies and clinical trials to evaluate the suicidal death of erythrocytes in tissue and organ contexts represents a key objective to be pursued.

### Eryptosis inhibitors

A growing body of evidence from ex vivo experiments and animal models highlights the role of biomolecules or synthetic compounds, whether from dietary sources or administered as pharmaceuticals, respectively, in mitigating eryptosis (Table S5). These molecules exhibit a range of biological activities, such as antioxidant, antiinflammatory, and ion-modulating effects, which collectively contribute to the inhibition of the apoptotic process. By targeting key cellular mechanisms, including oxidative stress, calcium influx, and membrane phospholipid asymmetry, these agents offer potential therapeutic uses for disorders associated with excessive erythrocyte death, such as hemolytic anemias, CKD, and metabolic syndromes. However, in vivo studies primarily in humans are needed to confirm these promising preclinical findings. Moreover, blocking eryptosis with these bioactive molecules or compounds could offer a new therapeutic or complementary approach to prevent diseases linked to elevated levels of circulating eryptotic RBCs. Consequently, adopting an appropriate dietary style rich in functional foods could provide a preventive strategy against eryptosis.

## Challenges, direction for further research, and concluding remarks

Since the term *eryptosis* was coined in 2005 by a group of researchers from the University of Tübingen (Germany) led by Florian Lang [24], a steady and continuous stream of novel papers focusing on eryptosis has been observed. The search using the PubMed platform revealed over 600 relevant papers on eryptosis at the end of 2024. Undoubtedly, eryptosis represents a distinct RCD of erythrocytes differing from senescence, hemolysis, and necroptosis. Recent studies have pushed forward the frontiers of our knowledge on eryptosis, revealing that mature erythrocytes have a more complex cell death signaling network than conventionally thought. This intricate eryptosis-regulating network seems to be simpler and less intractable than the complex apoptosis-regulating mechanisms. However, it is still sufficient to ensure rapid clearance of damaged erythrocytes and prevent their lytic cell death. Eryptosis is defined as an RCD modality of mature erythrocytes critically dependent on Ca^2+^ signaling, associated with cell shrinkage, membrane blebbing, and PS externalization. Thus, confirmation of eryptosis requires identification of at least PS externalization and elevation of intracellular Ca^2+^ levels.

Compelling evidence indicates that ion transport is of fundamental importance for eryptosis [90]. Ca^2+^ is a master regulator of eryptosis, mediating the Gardos effect-associated RBC dehydration and reduction of cell volume (cell shrinkage), calpain activation-mediated cytoskeleton remodeling (membrane blebbing), and PS externalization, which are major hallmarks of eryptosis. Eryptosis shrinkage is mediated by K^+^ efflux through Gardos channels. Additionally, deprivation of Cl^-^ facilitates Ca^2+^ influx by promoting the release of PGE2 [91]. It has been clear that due to the lack of epigenetic, transcriptional, and translational regulation of eryptosis, ion transport and adjustment of cation channels to fine-tune ion fluxes have come to the forefront in this RCD of erythrocytes. However, to deepen our knowledge of eryptosis regulation, investigations aiming at uncovering the role of ion transport in eryptosis should be endorsed. Additionally, involvement of multiple kinases in eryptosis regulation might suggest that post-translational modification (phosphorylation) might also be important in eryptosis regulation. Intensive research efforts are required to meet the demand of identifying novel eryptosis-associated kinases and investigating their potential pharmaceutical targetability. Moreover, little is currently known about the downstream effectors of eryptosis-associated kinases.

Notably, ROS signaling is involved in senescence, necroptosis of erythrocytes, and eryptosis. It is not clear how ROS contributes to the selection of a particular cell fate in erythrocytes, which might be an interesting topic for further research, especially given that sources of ROS differ in erythrocytes as compared with nucleated cells [54].

There is compelling evidence that the MOMP regulated by Bcl-2 family proteins is a pivotal checkpoint in apoptosis [492, 493]. Like in the case of apoptosis, the eryptotic threshold is probably dictated by a combination of proeryptotic and antieryptotic inputs. Is Ca^2+^ elevation a critical point that irreversibly switches the erythrocyte program towards inevitable death?

Experimental evidence supporting extrinsic Fas-mediated eryptosis suggests that eryptosis can be induced by external perturbations. Moreover, eryptosis has been demonstrated to be induced by NPs [459] or high-molecular-weight polysaccharides [494] without their uptake but with accumulation around the cells, suggesting that eryptosis might be induced by shear stress via piezo1 mechanosensitive Ca^2+^ ion channels.

Comprehensive analysis supports the fundamental difference between senescence and eryptosis, evidenced primarily by much faster clearance of eryptotic cells compared to senescent erythrocytes [78]. It is challenging to define cellular senescence in erythrocytes, since these cells are terminally differentiated and their aging is not accompanied by the senescence-associated secretory phenotype (SASP) like in the case of nucleated cells [495]. Since the inability to proliferate and the emergence of SASP are key events defining cellular senescence in nucleated cells, it is a much more rigorous task to distinguish senescence and eryptosis in RBCs than senescence and apoptosis in nucleus-containing cells. However, to simplify research interpretation in the field, PS externalization may be considered a decent marker of eryptosis, indicating the occurrence of this RCD even in aged cells expressing senescence-associated markers. Further studies are required to provide more evidence about the differences between RBC senescence and eryptosis.

Discovery of eryptosis fueled an interest in the investigation of the cell death signaling network in erythrocytes, resulting in the discovery of a regulated necrosis of erythrocytes [25]. Nowadays, the distinctness between eryptosis and necroptosis of erythrocytes has been firmly established, and it can be assumed that erythrocytes have a branched cell death network. Identification of cell-specific features of erythrocyte necroptosis has led to announcing the term *erythronecroptosis* to define a regulated necrosis of erythrocytes [54]. Complex cell death signaling networks revealed in erythrocytes have fueled an interest in unveiling the crosstalk between lethal subroutines. Mounting evidence on the mutual exclusiveness of eryptosis and necroptosis indicates that erythrocytes are capable of context-dependent decision-making in response to internal and external perturbations. We believe that significant efforts should be put into investigating in greater detail the pathways shared by eryptosis and erythronecroptosis, like caspase-8, ROS signaling, Fas/FasL signaling, to collect more evidence on the interplay between these erythrocyte-specific RCDs. A more complex decision landscape revealed in erythrocytes might shift the equilibrium in the discussion of perception of mature erythrocytes as living or non-living entities [4] in the direction of the former.

Among over 20 distinct RCD modalities reported, ferroptosis, described as a ROS-dependent non-apoptotic iron-driven cell death [496], has attracted considerable attention as a potential therapeutic target in cancer [497]. Erythrocytes are filled with iron-containing hemoglobin, prone to oxidative stress, critical for ferroptosis, and contain GPX4, an important regulator of ferroptosis [78, 498]. Importantly, ferroptosis has been shown to be exploited by RBC precursors, regulating their differentiation [40]. At the same time, erythrocytes lack components of the ferroptosis execution system. Currently available data suggest that iron overload in hemochromatosis patients is associated with PS externalization and calpain activation, typical hallmarks of eryptosis [83]. At the moment, it is not clear whether iron overload induces eryptosis in erythrocytes or might trigger a unique RCD similar to ferroptosis of nucleated cells. However, it can be stated with a certain degree of confidence that iron overload-dependent cell death of erythrocytes, if it exists, will significantly differ from its counterpart in nucleated cells.

Uniqueness of the eryptosis phenomenon as a cell death of enucleated cells should be put under scrutiny by comparing eryptosis to RCD of platelets, which constitute the other enucleated blood entities [78]. Platelet intrinsic apoptosis relies on the mitochondrial apoptosome-mediated caspase-9/caspase-3-dependent mechanism, is tightly regulated by Bcl-XL, Bak, and Bax, and lacks the Fas receptor present on erythrocytes [499]. Not surprisingly, cell-specific features of eryptosis are rather associated with the clearance of the mitochondria in reticulocytes, and not with the enucleation process in RBC precursors during maturation. In addition, identification of novel differences between eryptotic and apoptotic lethal subroutines might expand our opportunities to discover selectively targetable pathways for therapeutics to modulate eryptosis. More and more studies reveal the role of eryptosis in human pathology, indicating that accelerated eryptosis contributes to anemia, enhanced blood clotting, and endothelial dysfunction. Eryptosis inhibition seems to be a promising strategy to prevent such complications. The issue of eryptosis druggability is of huge importance. Since the above-mentioned eryptosis-associated detrimental effects are mainly associated with PS exposure, the eryptosis-targeting drugs should prevent PS externalization. Search for novel steps and molecules that could be therapeutically targeted should be prioritized to translate scientific discoveries in the field “from bench to bedside”. Some studies have provided insights that eryptosis might be of diagnostic and prognostic significance. In addition, it may also serve as a tool for assessing the effectiveness of treatment [243, 287, 500].

Moreover, the field of eryptosis research focuses primarily on the preclinical in vitro evaluation of compounds that might theoretically induce eryptosis. Thus, the lack of in vivo experiments significantly impedes progress. Immunogenic consequences of apoptotic cell death, as well as necroptotic death of erythrocytes, seem to be an underappreciated topic, especially taking into account that erythrocytes are the most abundant circulating cells of the human body.

In conclusion, eryptosis targeting is a promising therapeutic approach in multiple human diseases that should not be neglected. This review aims to summarize the currently available data on eryptosis and provides a generally accepted nomenclature in order to add fresh impetus to the field. Moreover, we believe that recent advances in understanding the complexity and uniqueness of this distinct cell death might question the status of erythrocytes as entities between life and death.

## Supplementary information

Supplementary file
