# Supplementary material for: Current understanding of eryptosis: mechanisms, physiological functions, role in disease, pharmacological applications, and nomenclature recommendations
Source: Cell Death Dis. 2025 Jul 1;16(1):467. doi: 10.1038/s41419-025-07784-w (PMC12216432; doi:10.1038/s41419-025-07784-w)
Supplement: Supplementary file 1 — Supplementary file [file 41419_2025_7784_MOESM1_ESM.docx]

**Current understanding of eryptosis: mechanisms, physiological functions, role in disease, pharmacological applications, and nomenclature recommendations**

Anton Tkachenko^1,2^, Mohammad A. Alfhili^3^, Jawaher Alsughayyir^4^, Alessandro Attanzio^5^, Abdulla Al Mamun Bhuyan^6^, Bożena Bukowska^7^, Antonio Cilla^8^, Martha A. Quintanar-Escorza^9^, Michael Föller^10^, Ondrej Havranek^2,11^, Kashif Jilani^12^, Anatolii Onishchenko^1^, Etheresia Pretorius^13,14^, Volodymyr Prokopiuk^1,15^, Ignazio Restivo^5^, Luisa Tesoriere^5^, Grazia Maria Virzì^16,17^, Thomas Wieder^18^

^1^Department of Cryobiochemistry, Institute for Problems of Cryobiology and Cryomedicine of the National Academy of Sciences of Ukraine, Kharkiv 61015, Ukraine

^2^BIOCEV, First Faculty of Medicine, Charles University, Průmyslová 595, 25250 Vestec, Czech Republic

^3^Chair of Medical and Molecular Genetics Research, Department of Clinical Laboratory Sciences, College of Applied Medical Sciences, King Saud University, Riyadh 12372, Saudi Arabia

^4^Department of Clinical Laboratory Sciences, College of Applied Medical Sciences, King Saud University, Riyadh 12372, Saudi Arabia

^5^Department of Biological, Chemical and Pharmaceutical Sciences and Technologies, University of Palermo,

Via Archirafi 28, 90123 Palermo, Italy

^6^Department of Veterinary and Animal Sciences, University of Rajshahi-6205, Bangladesh

^7^University of Lodz, Faculty of Biology and Environmental Protection, Department of Biophysics of Environmental Pollution, Pomorska Str. 141/143, 90-236 Lodz, Poland

^8^Nutrition and Food Science Area, Faculty of Pharmacy and Food Sciences, University of Valencia, Avda. Vicente Andrés Estellés s/n, 46100 Burjassot, Spain

^9^Medicine and Nutrition Faculty, Universidad Juárez del Estado de Durango, Av. Universidad s/n, Los Angeles, 34076 Durango, Dgo; México

^10^Department of Physiology, University of Hohenheim, Garbenstr. 30, 70599 Stuttgart, Germany

^11^First Department of Internal Medicine-Hematology, General University Hospital and First Faculty of Medicine, Charles University, Prague, Czech Republic

^12^Department of Biochemistry, University of Agriculture, 03802 Faisalabad, Pakistan

^13^Department of Physiological Sciences, Faculty of Science, Stellenbosch University, Stellenbosch 7600, South Africa

^14^Department of Biochemistry and Systems Biology, Institute of Systems, Molecular and Integrative Biology, Faculty of Health and Life Sciences, University of Liverpool, L69 7ZB, UK

^15^Research Institute of Experimental and Clinical Medicine, Kharkiv National Medical University,

Nauky ave 4, 61022 Kharkiv, Ukraine

^16^Department of Nephrology, Dialysis and Transplant, St Bortolo Hospital, Viale F Rodolfi, 37, 36100 Vicenza, Italy

^17^IRRIV - International Renal Research Institute Vicenza, Viale F Rodolfi, 37, 36100 Vicenza, Italy

^18^Institute of Physiology I, Eberhard Karls University Tübingen, Wilhelmstr. 56, 72074 Tübingen, Germany

Table S1

**Composition of Ringer buffers**

| **Ingredient** | **Standard** | **Ca^2+^-free** | **KCl** | **Urea** | **Glucose** | **Sucrose** | **Mannitol** | **Adenine** | **Guanosine** | **Dextran** | **PEG** | **Trolox** | **Heparin** |
| --- | --- | --- | --- | --- | --- | --- | --- | --- | --- | --- | --- | --- | --- |
| **NaCl** | 8.0 | 8.0 | – | 8.2 | 8.0 | 7.6 | 7.6 | 8.0 | 8.0 | 8.0 | 8.0 | 8.0 | 8.0 |
| **KCl** | 0.37 | 0.37 | 137 | 0.37 | 0.37 | 0.37 | 0.37 | 0.37 | 0.37 | 0.37 | 0.37 | 0.37 | 0.37 |
| **CaCl_2_** | 0.11 | 0.11 | 0.11 | 0.11 | 0.11 | 0.11 | 0.11 | 0.11 | 0.11 | 0.11 | 0.11 | 0.11 | 0.11 |
| **MgSO_4_** | 0.12 | 0.12 | 0.12 | 0.12 | 0.12 | 0.12 | 0.12 | 0.12 | 0.12 | 0.12 | 0.12 | 0.12 | 0.12 |
| **Glucose** | 0.9 | 0.9 | 0.9 | 0.9 | 4.5 | 0.9 | 0.9 | 0.9 | 0.9 | 0.9 | 0.9 | 0.9 | 0.9 |
| **HEPES** | 7.62 | 7.62 | 7.62 | 7.62 | 7.62 | 7.62 | 7.62 | 7.62 | 7.62 | 7.62 | 7.62 | 7.62 | 7.62 |
| **Urea** | – | – | – | 1.5 | – | – | – | – | – | – | – | – | – |
| **Sucrose** | – | – | – | – | – | 13.6 | – | – | – | – | – | – | – |
| **Mannitol** | – | – | – | – | – | – | 6 | – | – | – | – | – | – |
| **Adenine** | – | – | – | – | – | – | – | 0.27 | – | – | – | – | – |
| **Guanosine** | – | – | – | – | – | – | – | – | 0.56 | – | – | – | – |
| **Dextran 500,000 MW** | – | – | – | – | – | – | – | – | – | 10 | – | – | – |
| **PEG 8,000** | – | – | – | – | – | – | – | – | – | – | 10 | – | – |
| **Trolox** | – | – | – | – | – | – | – | – | – | – | – | 0.12 | – |
| **Heparin** | – | – | – | – | – | – | – | – | – | – | – | – | 0.2 |
| **Osmolarity** | 326 | 323 | 316 | 357 | 346 | 352 | 345 | 328 | 328 | 326 | 338 | 312 | 312 |

**Note:**

- All values are in g/L. Dextran and PEG are in *w*/*v*. Osmolarity is in mOsm/L.
- Combinations of the above ingredients are possible (e.g., Ca^2+^-free and K^+^-rich) provided that the resulting buffer remains isosmotic. This requires modifying the concentrations of each ingredient based on its nature (a penetrating or a non-penetrating solute) and on the intended purpose.
- Unique interactions with the studied toxicant must be borne in mind when formulating these buffers as lower or higher concentrations of the added ingredient(s) may be required.
- It is important to consider that some ingredients may be antihemolytic but proeryptotic (e.g., PEG) and vice versa.
- Urea, sucrose, glucose, and mannitol may be increased to study the behavior of the compound of interest under conditions of hyperosmotic stress.
- Lipid-rich bovine serum albumin “Albumax” may be added at 5 g/L as it contains pro-survival factors and has a drug-binding capacity^1^.
- ATP may be added at 0.12 or 0.25 g/L^2^.
- Caffeine may be added at 0.1 g/L^3^.
- Uric acid (0.168 g/L) may also be added as it demonstrates cytoprotective effects^4^.

Table S2

**Modulators of eryptosis**

| **Target** | **Modulators** | **Effect** | **References** |
| --- | --- | --- | --- |
| **I. Surface proteins** | | | |
| K^+^ (Gardos) channels | Senicapoc; clotrimazole | Inhibition | ^5^,^6^ |
| Nonselective cation (Ca^2+^ and Na^+^) channels; Ca^2+^-permeable cation/Na^+^ channel;  Na^+^/H^+^ exchanger | Amiloride; amlodipine; HOE 642 and HOE 694; EPO | Inhibition | ^7^,^8^,^9^ |
| CaCC | Niflumic acid; CaCCinh-A01 | Inhibition | ^10^,^11^ |
| Na^+^/K^+^-ATPase pump | Ouabain; rostafuroxin | Inhibition | ^12^,^13^ |
| AE1 | DIDS | Inhibition | ^14^ |
| GLUT1 | WZB117 | Inhibition | ^15^ |
| EPOR | Anti-EpoR | Inhibition | ^16^ |
| TNFα | J1D9 (anti-TNFα) | Inhibition | ^17^ |
| FasL (CD178) | NOK-1 (anti-FasL) | Inhibition | ^17^ |
| TRAIL (CD253) | RIK-2 (anti-TRAIL) | Inhibition | ^17^ |
| PAF receptor | Apafant (WEB2086); foropafant (SR27417) | Inhibition | ^18^,^19^ |
| TAUT | Guanidinoethyl sulfonate (taurocyamine) | Inhibition | ^20^ |
| **II. Eryptosis mediators** | | | |
| Intracellular Ca^2+^ | BAPTA-AM | Inhibition | ^21^ |
| Caspase | zVAD; emricasan | Inhibition | ^22^,^23^ |
| p38 MAPK | SB203580; p38 inhibitor III | Inhibition | ^24^ |
| CK1α | D4476 | Inhibition | ^22^ |
| PKC | Staurosporine | Inhibition | ^25^ |
| Rac1 GTPase | NSC 23766 | Inhibition | ^26^ |
| COX | Acetylsalisylic acid; ibuprofen | Inhibition | ^27^ |
| PLA_2_ | Quinacrine | Inhibition | ^28^ |
| Serine palmitoyltransferase | Myriocin (Thermozymocidin) | Inhibition | ^29^ |
| Lipoxygenase | Bay-Y5884 | Inhibition | ^30^ |
| JAK3 | JANEX-1 | Inhibition | ^31^ |
| MSK1/2 | SB-747651-A | Inhibition | ^32^ |
| Akt | LY294002 | Inhibition | ^26^ |
| μ-Calpain | Calpain inhibitors I and II | Inhibition | ^27^ |
| AMPK | Compound C; resveratrol | Inhibition | ^33^ |
|  | AICAR phosphate; cordycepin | Activation | ^33^,^34^ |
| cGKI | 8-Bromo-cGMP | Inhibition | ^35^,^36^ |
|  | 8-pCPT-cGMP, TEA | Activation |  |
| PAK2 | FRAX486; G-5555 | Inhibition | ^37^,^38^ |
| PDK1 | BX-320 | Inhibition | ^39^ |
| CDK4 | CDK4 inhibitor II | Inhibition | ^40^ |
| **III. Necroptosis mediators** | | | |
| RIPK1 | Necrostatin-1 | Inhibition | ^41^ |
| RIPK3 | GSK’872; HS-1371 | Inhibition | ^17^, ^42^ |
| MLKL | Necrosulfonamide | Inhibition | ^43^ |
| Syk | Bay 61-3606 | Inhibition | ^44^ |
| Src | PP1 | Inhibition | ^17^ |
| **IV. ROS/RNS neutralization** | | | |
| – | Vitamin C |  | ^45^ |
| – | Vitamin E |  | ^46^ |
| – | Trolox |  | ^47^ |
| – | GSH |  | ^48^ |
| – | NAC |  | ^49^ |
| – | Melatonin |  | ^50^ |
| – | Catalase |  | ^51^ |
| AGEs | Pyridoxamine | Inhibition | ^44^ |
| NADPH oxidase | VAS2870; DPI | Inhibition | ^26^ |
| NOS | L-NAME | Inhibition | ^26^ |
| NO donor | Nitroprusside; papanonoate |  | ^52^ |

**Note:** AE1, Anion exchanger 1; AGEs, Advanced glycation end products; Akt, Protein kinase B; AMPK, AMP-activated protein kinase; CaCC, Ca^2+^-activated Cl^-^ channels; cGKI, cGMP-dependent protein kinase type I; cGMP, Cyclic guanosine monophosphate; CK1α, Casein kinase 1 alpha; CDK4, Cyclin-dependent kinase 4; COX, Cyclooxygenase; DIDS, 4,4′-Diisothiocyanatostilbene-2,2′-disulfonic acid; DPI, Diphenyleneiodonium; EPOR, Erythropoietin receptor; FasL, Fas ligand, GLUT1, Glucose transporter 1; GSH, Reduced glutathione; JAK3, Janus kinase 3; MLKL, Mixed lineage kinase domain like pseudokinase; MSK1/2, Mitogen- and stress-activated protein kinase; NAC, N-acetyl-L-cysteine; NO, Nitric oxide; NOS, Nitric oxide synthase; p38 MAPK, p38 Mitogen-activated protein kinase; PAF, Platelet-activating factor; PAK2, p21-Activated kinase 2; PDK1, Phosphoinositide-dependent kinase-1; PKC, Protein kinase C; PLA_2_, Phospholipase A_2_; RIPK1, Receptor-interacting serine/threonine-protein kinase 1; RIPK3, Receptor-interacting serine/threonine-protein kinase 3; RNS, Reactive nitrogen species; ROS, Reactive oxygen species; Syk, Spleen-associated tyrosine kinase; TAUT, Taurine transporter; TEA, Tetraethylammonium chloride; TNFα, Tumor necrosis factor alpha; TRAIL, TNF-related apoptosis-inducing ligand.

Table S3

**Investigation of eryptosis inhibition**

| **Mechanism** | **Approach** | **References** |
| --- | --- | --- |
| Membrane rupture | Hemolytic agents (e.g., AAPH) | ^53^ |
| Ca^2+^ influx | Ca^2+^ ionophores (ionomycin or A23187) | ^9^,^54^ |
| Oxidative stress | Oxidants (e.g., hydrogen peroxide, tBuOOH, PEITC, etc.) | ^55^,^56^ |
| Hyperosmotic shock | Sucrose addition | ^24^ |
| Metabolic exhaustion | Glucose elimination | ^56^ |
| Cl^-^ efflux | Cl^-^ elimination | ^57^ |
| Ceramide formation | Sphingomyelinase or C_6_ ceramide | ^52^ |
| Heat shock | Incubation at 43-55 ^o^C for 30 min | ^58^ |

**Note:** AAPH, 2,2′-Azobis (2-methylpropionamidine) dihydrochloride; tBuOOH, *tert*-butyl hydroperoxide; PEITC, 2-Phenethyl isothiocyanate.

Table S4

**Stimulators that significantly induce eryptosis mainly obtained from Lang’s group investigations**

| **Compound** | **Treatment** | | **Mechanism** | | | **Reference** |
| --- | --- | --- | --- | --- | --- | --- |
|  | **Concentration** | **Time** | **Calcium** | **Ceramide** | **Other** |  |
| ***Inhibitors/Activators*** | | | | | | |
| Bay 11-7082 | 7-20 µM | 6-48 h | + |  | GSH | ^59^ |
| Bay-Y5884 | 20-40 μM | Ca^2+^: 1-6 h  Annexin: 24 h | + |  |  | ^30^ |
| BI2536 | 25-100 µM | 48 h | + | + |  | ^60^ |
| IPA3 | 5 μM | 48 h |  |  | PAK2 | ^61^ |
| Phorbol-12 myristate-13 acetate | 6 μM | Ca^2+^: 1-30 min  Annexin: 30 min | + |  | PKC | ^62^ |
| Vanadate | 5-20 μg/mL | 48 h | + |  | ATP | ^63^ |
| ***Biomolecules/Enzymes/Nutrients*** | | | | | | |
| 4-Hydroxynonenal | 10-100 µM | 24 h | + | + | PGE_2_  Caspase-3 | ^64^ |
| 15-Deoxy-delta-12,14-prostaglandin J2 | 3-10 µM | Ca^2+^: 6 h  Annexin: 24 h | + |  |  | ^65^ |
| α-Lipoic acid | 20-100 µM | 48 h | + | + | ATP  Caspase-3 | ^66^ |
| Adarotene | 6-9 µM | 48 h | + | + | ROS | ^67^ |
| Amyloid | 0.1-2 μM | 24 h |  | + |  | ^68^ |
| Anandamide | 2.5-10 μM | Ca^2+^: 60-180-360 min  Annexin: 24 h | + |  |  | ^69^ |
| Anti-A IgG | 0.5 μg/mL | Ca^2+^: 6 h  Annexin: 24 h | + |  |  | ^70^ |
| Ceramide (acylsphingosine) | 50 μM | 4-8 h |  |  | Hyperosmotic shock | ^71^ |
| Glycation | 40-100 mM glucose | 48 h | + |  |  | ^72^ |
| Hemin | 1-10 μM | 48 h | + | + |  | ^73^ |
| Leukotriene C(4) | 5-10 µM | Ca^2+^: 5-60 min  Annexin/Ca^2+^: 48 h | + |  | Caspase-8 and caspase-3 | ^74^ |
| Lipopeptides | 1-10 µg/mL | 48 h | + | + |  | ^75^ |
| Lysophosphatidic acid | 2.5 μM | Ca^2+^: 1-30 min  Annexin:15-120 min | + |  | PKC | ^62^ |
| Methylglyoxal | 0.3-300 μM | 6-48 h |  |  | ATP  GSH | ^76^ |
| Mushroom tyrosinase | 5-7 U/mL | 24 h | + | + |  | ^77^ |
| Oxysterols | 2-20 μM | 2-48 h | + |  | ROS  Nitric oxide  GSH  Rac GTPase  PKC  PGE_2_ | ^78^,^26^ |
| PAF | 0.5-3.8 μM | 30 min-24 h | + | + |  | ^79^ |
| Peptidoglycan | 5-100 μg/mL | 48 h | + | + | ATP | ^80^,^81^ |
| Phosphate | 0.5-5 mM | 48 h | + |  |  | ^82^ |
| Phosphatidic acid | 1-25 µM | 15 min | + |  | ATP  PKC | ^83^ |
| PGE_2_ | Annexin: 20-50 µM  Ca^2+^: 0.1 nM-50 µM | 20 min-24 h  1 min-4 h | + |  |  | ^27^ |
| Retinoic acid | 3-10 µM | 24 h | + |  |  | ^84^ |
| Selenium (sodium selenite) | 100-500 µg/L | 48 h | + | + |  | ^85^ |
| Sphingomyelinase | 1-10 mU/mL | 6 h | + | + |  | ^86^ |
| Sphingosine | 10 μM | 48 h | + |  |  | ^87^ |
| Vitamin K(3) | 1-30 µM | 48 h |  | + | ATP | ^88^ |
| Zinc | 10-50 µM | 6-24 h | + | + |  | ^89^ |
| ***Natural compounds/Phytochemicals*** | | | | | | |
| Apigenin | 1-15 μM | 48 h | + | + | ATP | ^90^ |
| Aristolochic Acid | 75-100 μg/mL | 48 h | + | + |  | ^91^ |
| Betulinic acid | 15-240 μM | 3-24 h | + |  |  | ^92^ |
| Cordycepin | 30-250 µM | 24 h | + |  |  | ^93^ |
| Costunolide | 10-15 μg/mL | 48 h | + | + | ROS | ^94^ |
| Cryptotanshinone | 5-10 μM | 48 h | + |  |  | ^95^ |
| Curcurmin | 0.1-10 μM | 0.5-24 h | + | + |  | ^96^ |
| Dermaseptin | 50 μM | 48 h | + | + |  | ^97^ |
| Fascaplysin | 1-10 µM | 48 h | + | + | ROS | ^98^ |
| Ferutinin | 10-40 µM | 24 h | + |  |  | ^99^ |
| Gambogic acid | 100-500 nM | 48 h | + | + |  | ^100^ |
| Gedunin | 12-24 µM | 48 h | + |  |  | ^101^ |
| Gossypol | 0.25-1 μM | 48 h | + |  |  | ^102^ |
| Honokiol | 5-15 μM | 6-48 h | + | + |  | ^103^ |
| Myricetin | 2-8 µM | 24 h | + | + | ROS | ^104^ |
| Naphthoquinone derivatives | 10-50 μM | 4-24 h |  |  | ATP  ROS  GSH | ^105^ |
| Oridonin | 10-50 µM | 48 h | + | + |  | ^106^ |
| Parthenolide | 30-100 μM | 6-48 h | + |  | Interference with NF-κB signaling | ^59^,^107^ |
| Penta-O-galloyl-β-D-glucose | 10-50 μM | 48 h | + | + |  | ^108^ |
| Phloretin | 100-300 μM | 48 h |  | + |  | ^109^ |
| Physcion | 25-100 µM | 24 h | + |  |  | ^110^ |
| Phytic acid | 1-5 mM | 48 h |  |  | ATP | ^111^ |
| Piceatannol | 10-20 µM | 48 h |  | + | ROS | ^112^ |
| Plumbagin | 0.5-2 μM | Ca^2+^: 1-48 h  Annexin: 48 h | + | + |  | ^113^ |
| Polyphyllin D | 0.32-2.5 µM | 24 h | + |  | Caspase-3  Membrane permeabilization | ^114^ |
| Rotenone | 1-10 μM | 48 h | + | + |  | ^115^ |
| Shikonin | 0.5-1 μM | 24-48 h | + | + | ATP | ^116^ |
| Tannic acid | 1-50 µM | 48 h |  | + |  | ^117^ |
| Tanshinone IIA | 10-25 μM | 48 h | + | + | ATP | ^118^ |
| Thymoquinone | 3-20 µM | 48 h |  |  | Unknown mechanism | ^119^ |
| Trans-cinnamaldehyde | 20-30 µM | 48 h | + |  |  | ^120^ |
| Ursolic acid | 1-10 µM | 48 h | + | + |  | ^121^ |
| Withaferin A | 5-10 μM | 48 h | + | + | ROS | ^122^ |
| ***Drugs*** | | | | | | |
| Afatinib | 4-8 μg/mL | 48 h | + | + | ROS | ^123^ |
| Amantadine | 0.2-1 μg/mL | 48 h | + |  |  | ^124^ |
| Amiodarone | 0.01-1 μM | Ca^2+^: 60-180-360 min  Annexin: 24 h  Hyperosmotic shock: 6 h | + |  |  | ^125^ |
| Amphotericin B | 0.1-1 μg/mL | 48 h | + |  | ATP | ^126^ |
| Artesunate | 6-35 μg/mL | 48 h | + | + | ROS | ^127^ |
| Auranofin | 5-10 μg/mL | 24 h |  |  | ROS | ^128^ |
| Azathioprine | 1-10 μg/mL | 48 h | + |  |  | ^129^, ^130^ |
| Benzethonium | 2.5 -5 μM | 48 h | + |  |  | ^131^ |
| Carmustine | 25-100 μM | 48 h | + |  |  | ^132^ |
| Celecoxib | 10-15 μM | 48 h | + |  |  | ^133^ |
| Chlorpromazine | 10-50 μM | Ca^2+^: 6 h  Annexin: 24 h  GSH: 24-48 h | + |  | ATP  GSH  Hyperosmotic shock | ^134^ |
| Ciglitazone | 2-10 μM | Ca^2+^: 6 h  Annexin: 24 h | + |  |  | ^65^ |
| Cisplatin | 1-10 µM | 48 h | + |  | ATP | ^135^ |
| Cyclosporine | 5-10 μM | 24-48 h |  | + | ATP | ^136^,^137^ |
| Dicoumarol | 1-30 μM | 48 h | + |  |  | ^138^ |
| Dimethylfumarate | 35-140 μM | 24-48 h | + |  | GSH | ^139^ |
| Estramustine | 50-100 µM | 24-72 h | + | + |  | ^140^ |
| Fluoxetine | 25-50 μM | 48 h | + |  |  | ^141^ |
| FTY720 | 5-10 μM | 48 h | + |  |  | ^142^ |
| Gefitinib | 2-3 μg/mL | 48 h |  |  | ROS | ^143^ |
| Geldanamycin | 5-50 μM | 48 h |  | + |  | ^144^ |
| Ipratropium bromide | 0.4-1 nM | 48 h | + |  |  | ^145^ |
| Lithium | 0.1-3 mM | 1-48 h | + |  |  | ^146^ |
| Lumefantrine | 3-24 μg/mL | 48 h |  |  | Unknown mechanism | ^147^ |
| Methyldopa | 6-50 μg/mL | 48 h |  | + | GSH | ^148^ |
| Miltefosine | 2.45-12.25 μM | 6-48 h | + |  |  | ^149^ |
| Mitotane | 5-15 μg/mL | 2.5-48 h | + |  |  | ^150^ |
| Mitoxantrone | 5-25 μg/mL | 48 h |  | + | ROS | ^151^ |
| Naproxen Sodium | 10-25 µM | 48 h | + |  | Oxidative stress | ^152^ |
| Nitazoxanide | 10-50 μg/mL | 48 h |  | + |  | ^153^ |
| Novobiocin | 500 μM | 48 h | + | + |  | ^154^ |
| Nystatin | 5-15 μg/mL | 48 h | + |  |  | ^155^ |
| Paclitaxel | 1-10 μM | 20 min-48 h | + | + |  | ^156^,^157^ |
| Pazopanib | 25-50 μg/mL | 48 h | + | + | ROS | ^158^ |
| Pyrvinium pamoate | 1-10 μM | 24-48 h | + |  |  | ^159^,^160^ |
| Ribavirin | 8-16 μg/mL | 48 h | + |  |  | ^161^ |
| Rifampicin | 24-120 μg/mL | 48 h | + | + |  | ^162^ |
| Salinomycin | 5-100 nM | 48 h | + |  | ROS | ^163^ |
| Simvastatin | 0.5-2 μg/mL | 48 h | + |  | ROS  p38 MAPK | ^164^ |
| Sorafenib | 0.5-10 μM | 48 h | + |  | ROS | ^165^ |
| Sulindac sulfide | 5-20 μM | 48 h | + | + |  | ^166^ |
| Sunitinib | 10-20 μM | 48 h | + |  | p38 MAPK  Caspases | ^167^ |
| Taurolidine | 15-60 μg/mL | 48 h | + | + |  | ^168^ |
| Thioridazine | 12-30 μM | 48 h | + |  | p38 MAPK | ^169^ |
| Topotecan | 125-175 μg/mL | 48 h | + | + |  | ^170^ |
| Triclosan | 25-100 µM | 1-4 h | + |  | p38 MAPK  RIPK1 | ^41^ |
| ***Toxins/Mycotoxins/Ionophores*** | | | | | | |
| A23187 | 2 µM | Ca^2+^: 5-30 min  Annexin: 15-120 min | + |  |  | ^62^ |
| Beauvericin | 1-10 μM | 48 h | + |  | ATP | ^171^ |
| Enniatin A | 1-5 μM | 48 h | + |  | ATP | ^172^ |
| Fumagillin | 10-100 μM | 48 h | + | + |  | ^173^ |
| Hemolysin | 0.1-0.5 U/mL | 0.5-10 h | + |  |  | ^174^ |
| Indoxyl sulfate | 50-600 μM | 48 h | + | + |  | ^175^ |
| Listeriolysin | 10-250 ng/mL | 30 min | + |  |  | ^176^ |
| Monensin | 0.5-10 µM | 24 h | + |  | ATP | ^177^ |
| Narasin | 1-25 ng/mL | 48 h | + | + |  | ^178^ |
| Ochratoxin A | 2.5-10 μM | 48 h | + | + |  | ^179^ |
| Patulin | 2.5-10 μM | 48 h | + |  |  | ^180^ |
| Valinomycin | 1 nM and 10 nM | 24 h |  |  | K^+^ loss | ^181^ |
| Zearalenone | 10-50 μM | 48 h | + |  |  | ^182^ |
| ***Heavy metals/Pollutants/Xenobiotics*** | | | | | | |
| Acrolein | 15-50 µM | 48 h | + | + |  | ^183^ |
| Aluminum | 100 µM | Ca^2+^: 24 h  Other: 21 days | + |  | ROS  GSH  Cell morphology | ^184^ |
| Arsenic | 7-10 μM | 48 h | + | + | ATP | ^185^ |
| Bismuth chloride | 0.5-2 μg/mL | 48 h | + | + |  | ^186^ |
| Bromfenvinphos | 100-350 µM | 48 h | + |  | ROS | ^187^ |
| Cadmium | 0.1-10 µg/mL | 48 h | + |  | ATP | ^188^ |
| Carbon monoxide | 5-10 μM | 24 h | + |  |  | ^189^ |
| Chromium | 10-20 μM | 48 h | + |  | ATP | ^190^ |
| Cigarette Smoke Extract | 5-20 % | Ceramide: 20 min-6 h  Annexin: 24 h |  | + | p38 MAPK  DISC  Caspase-8 and caspase-3 | ^191^ |
| Copper | 1-3 µM | 24 h |  | + |  | ^192^ |
| Gold chloride | 0.25-1 μg/mL | 48 h | + |  |  | ^193^ |
| Hexavalent chromium | 20 µM | 6-48 h | + |  | ATP  ROS | ^194^ |
| Lead | 0.3-3 µM | Ca^2+^: 20 min  Annexin: 24 h | + |  |  | ^6^ |
| Mercury | 1-10 μM | K^+^-selective conductance: 1-4 min  Annexin: 24 h |  | + | K^+^ loss | ^195^ |
| Nickel chloride | 5-10 mM | 24 h | + |  | ROS  p38 MAPK | ^196^ |
| Radiocontrast agent | 0.76-5 mM | 10 min-48 h | + |  |  | ^197^ |
| Silver ions | 50-1,000 nM | 48 h |  |  | ATP | ^198^ |
| Tin | 3-100 µM | 48 h | + | + | ATP | ^199^ |

**Note:** ATP, Adenosine triphosphate; DISC, Death-inducing signaling complex; GSH, Reduced glutathione; IPA3, PAK1 inhibitor; NF-κB, Nuclear factor kappa B; p38 MAPK, p38 Mitogen-activated protein kinase; PAF, Platelet-activating factor; PAK2, p21-Activated kinase 2; PGE2, Prostaglandin E2; PKC, Protein kinase C; RIPK1, Receptor-interacting serine/threonine-protein kinase 1; ROS, Reactive oxygen species.

Table S5

**Major inhibitors of eryptosis**

| **Compound** | **Treatment** | | |  | **References** |
| --- | --- | --- | --- | --- | --- |
|  | **Concentration** | **Inducer** | **Time** | **Parameters** |  |
| Adenosine | 10-100 µM | Glucose depletion | 24-48 h | FSC | ^200^ |
| *Antirhea Borbonica*  extract | 500 mg/L in zebrafish | Methylglyoxal | 24-48 h | ROS  Antiglycative | ^201^ |
| Acetylsalycilic acid | 50 µM | 4-Hydroxynonenal | 24 h | Ca^2+^ | ^64^ |
| Caffeine | 50-500 µM | Glucose depletion | 48 h | Ca^2+^ | ^3^ |
| Chelerythrine | 1-10 µM | Costunolide | 24 h | FSC | ^202^ |
| Erythropoietin | 760 pM | Osmotic shock | 4 h | ROS | ^8^ |
| Erythropoietin | 1-40 U/mL | H_2_O_2_ | 2 h | Ca^2+^, ROS | ^9^ |
| Fisetin | 15 mg/kg in rats | D-galactose | 6 weeks | ROS, GSH | ^203^ |
| Hydroxytyrosol | 1 µM | Lysophosphatidic acid | 24 h | Ca^2+^, ATP | ^204^ |
| Hydroxytyrosol | 5 µM | Mercury | 4 h | GSH, ATP | ^205^ |
| Indicaxanthin | 1-5 µM | Oxysterols | 48 h | ROS, GSH, PGE_2_, Ca^2+^, Ceramide | ^206^ |
| Indicaxanthin | 1-5 µM | CSE | 4 h | Ceramide, DISC, ATP | ^207^ |
| Isoproterenol | 10 µM | Osmotic shock | 24 h | Ca^2+^ | ^208^ |
| L-Carnitine | 200 umol/L | Uremic serum | 24-48 h | ROS, GSH | ^209^ |
| N-Acetylcysteine | 5 mM | Aluminum | 21 days | ROS, GSH, Ca^2+^ | ^184^ |
| Naringin | 20-40 µM | tBuOOH | 48 h | FSC, Ca^2+^ | ^210^ |
| Phlorizin | 10-100 µM | Glucose depletion | 0.5-48 h | FSC, Ca^2+,^ | ^211^ |
| Plant sterols | 22 µM | tBuOOH | 24-48 h | ROS, GSH | ^212^ |
| Plant sterols | 22 µM | CSE | 4 h | Ceramide, DISC | ^213^ |
| Pyrogallol | 2-8 µM | Glucose depletion | 0.5-48 h | FSC | ^214^ |
| Resveratrol | 10 µM | Glucose depletion | 48 h | Ca^2+^ | ^215^ |
| Resveratrol | 40 µM  in rabbit | Cyadox | 3h | ROS, GSH, LDH | ^216^ |
| Salidroside | 60-280 µM | H_2_O_2_ | 24 h | ROS, Ca^2+^, Caspase-3 | ^217^ |
| Staurosporine | 1 µM | Glucose depletion | 48 h | FSC, PKCɑ | ^218^ |
| Tamarind seed  Extract | 50–200 µg/mL | AAPH | 24 h | ROS, GSH, Ca^2+^ | ^219^ |
| Thymol | 2.5–20 µg/mL | tBuOOH | 0.5-48 h | FSC, Ca^2+^ | ^220^ |
| Urea | 650 mM | Osmotic shock | 6 h |  | ^57^ |
| Vitamin C | 1 mg/mL | H_2_O_2_ | 0.5 h | FSC, Caspase-3 | ^221^ |
| Vitamin C | 280 µM | Glucose depletion | 48 h | Ca^2+^ | ^45^ |
| Wogonin | 100 µM | Ionomycin, Osmotic shock, PEITC | 24–48 h | FSC, Ca^2+^, ROS | ^56^ |
| Xanthohumol | 0.25–1 µM | tBuOOH | 0.5-48 h | FSC, Ca^2+^ | ^55^ |
| Amitriptyline | 50-100 µM in mice | *Plasmodium*  *berghei* | 48 h | FSC, Ceramide | ^222^ |
| D4476 | 10 µM | tBuOOH | 48 h | CK1 isoform α, Ca^2+^ | ^159^ |
| Dibutyryl-cGMP | 1 mM | Ionomycin | 4 h | Ca^2+^ | ^52^ |
| Ethyl isopropyl amiloride | 100 µM | Ionomycin | 6 h |  | ^7^ |
| Flufenamic acid | 10 µM | *Plasmodium falciparum* | 48 h | FSC, Ca^2+^ | ^14^ |
| Furosemide | 10-100 µM | ATP depletion | 45 min | Ca^2+^ | ^223^ |
| Nitroprusside | 1 µM | Ionomycin | 4 h |  | ^52^ |
| p38 Inh III | 1 µM | Osmotic shock | 5 h | FSC, p38 MAPK, Ca^2+^ | ^24^ |
| Zidovudine | 2 µg/mL | Glucose depletion | 2 h | Ca^2+^ | ^224^ |

**Note:** AAPH, 2,2′-Azobis (2-methylpropionamide)-dihydrochloride; ATP, Adenosine triphosphate; cGMP, Cyclic guanosine monophosphate; CK1, Casein kinase 1; CSE, Cigarette smoke extract; DISC, Death-inducing signaling complex; FSC, Forward scatter; GSH, Reduced glutathione; , LDH, Lactate dehydrogenase; p38 MAPK, p38 Mitogen-activated protein kinase; PEITC, 2-Phenethyl isothiocyanate; PGE2, Prostaglandin E2; PKCɑ, Protein kinase C alpha; ROS, Reactive oxygen species; tBuOOH, *tert*-butyl hydroperoxide**.**

**References**

1. Boulet C, Gaynor TL, Carvalho TG. Eryptosis and Malaria: New Experimental Guidelines and Re-Evaluation of the Antimalarial Potential of Eryptosis Inducers. *Front Cell Infect Microbiol* 2021, **11:** 630812.

2. Alfhili MA, Alghareeb SA, Alotaibi GA, Alsughayyir J. Galangin Triggers Eryptosis and Hemolysis Through Ca(2+) Nucleation and Metabolic Collapse Mediated by PKC/CK1α/COX/p38/Rac1 Signaling Axis. *Int J Mol Sci* 2024, **25**(22).

3. Floride E, Föller M, Ritter M, Lang F. Caffeine inhibits suicidal erythrocyte death. *Cell Physiol Biochem* 2008, **22**(1-4)**:** 253-260.

4. Tzounakas VL, Anastasiadi AT, Arvaniti VZ, Lelli V, Fanelli G, Paronis EC*, et al.* Supplementation with uric and ascorbic acid protects stored red blood cells through enhancement of non-enzymatic antioxidant activity and metabolic rewiring. *Redox Biol* 2022, **57:** 102477.

5. Stocker JW, De Franceschi L, McNaughton-Smith GA, Corrocher R, Beuzard Y, Brugnara C. ICA-17043, a novel Gardos channel blocker, prevents sickled red blood cell dehydration in vitro and in vivo in SAD mice. *Blood* 2003, **101**(6)**:** 2412-2418.

6. Kempe DS, Lang PA, Eisele K, Klarl BA, Wieder T, Huber SM*, et al.* Stimulation of erythrocyte phosphatidylserine exposure by lead ions. *Am J Physiol Cell Physiol* 2005, **288**(2)**:** C396-402.

7. Lang KS, Myssina S, Tanneur V, Wieder T, Huber SM, Lang F*, et al.* Inhibition of erythrocyte cation channels and apoptosis by ethylisopropylamiloride. *Naunyn Schmiedebergs Arch Pharmacol* 2003, **367**(4)**:** 391-396.

8. Myssina S, Huber SM, Birka C, Lang PA, Lang KS, Friedrich B*, et al.* Inhibition of erythrocyte cation channels by erythropoietin. *J Am Soc Nephrol* 2003, **14**(11)**:** 2750-2757.

9. Vota DM, Maltaneri RE, Wenker SD, Nesse AB, Vittori DC. Differential erythropoietin action upon cells induced to eryptosis by different agents. *Cell Biochem Biophys* 2013, **65**(2)**:** 145-157.

10. Myssina S, Lang PA, Kempe DS, Kaiser S, Huber SM, Wieder T*, et al.* Cl- channel blockers NPPB and niflumic acid blunt Ca(2+)-induced erythrocyte 'apoptosis'. *Cell Physiol Biochem* 2004, **14**(4-6)**:** 241-248.

11. Tian XQ, Ma KT, Wang XW, Wang Y, Guo ZK, Si JQ. Effects of the Calcium-Activated Chloride Channel Inhibitors T16Ainh-A01 and CaCCinh-A01 on Cardiac Fibroblast Function. *Cell Physiol Biochem* 2018, **49**(2)**:** 706-716.

12. Huang S, Dong W, Lin X, Xu K, Li K, Xiong S*, et al.* Disruption of the Na(+)/K(+)-ATPase-purinergic P2X7 receptor complex in microglia promotes stress-induced anxiety. *Immunity* 2024, **57**(3)**:** 495-512.e411.

13. Xiong M, Liu X, Liang T, Ban Y, Liu Y, Zhang L*, et al.* The Alpha-1 Subunit of the Na(+)/K(+)-ATPase (ATP1A1) Is a Host Factor Involved in the Attachment of Porcine Epidemic Diarrhea Virus. *Int J Mol Sci* 2023, **24**(4).

14. Kasinathan RS, Föller M, Koka S, Huber SM, Lang F. Inhibition of eryptosis and intraerythrocytic growth of Plasmodium falciparum by flufenamic acid. *Naunyn Schmiedebergs Arch Pharmacol* 2007, **374**(4)**:** 255-264.

15. Zhang Z, Tai Y, Liu Z, Pu Y, An L, Li X*, et al.* Effects of d-ribose on human erythrocytes: Non-enzymatic glycation of hemoglobin, eryptosis, oxidative stress and energy metabolism. *Blood Cells, Molecules, and Diseases* 2023, **99:** 102725.

16. Elliott S, Busse L, McCaffery I, Rossi J, Sinclair A, Spahr C*, et al.* Identification of a sensitive anti-erythropoietin receptor monoclonal antibody allows detection of low levels of EpoR in cells. *J Immunol Methods* 2010, **352**(1-2)**:** 126-139.

17. LaRocca TJ, Stivison EA, Mal-Sarkar T, Hooven TA, Hod EA, Spitalnik SL*, et al.* CD59 signaling and membrane pores drive Syk-dependent erythrocyte necroptosis. *Cell Death & Disease* 2015, **6**(5)**:** e1773-e1773.

18. Zhang F, Liu L, Zhang H, Liu ZL. Effect of Platelet-Activating Factor on Barrier Function of ARPE-19 Cells. *Drug Des Devel Ther* 2020, **14:** 4205-4214.

19. Beaudeux JL, Said T, Ninio E, Ganné F, Soria J, Delattre J*, et al.* Activation of PAF receptor by oxidised LDL in human monocytes stimulates chemokine releases but not urokinase-type plasminogen activator expression. *Clin Chim Acta* 2004, **344**(1-2)**:** 163-171.

20. Suárez LM, Muñoz MD, González JC, Bustamante J, Del Río RM, Solís JM. The taurine transporter substrate guanidinoethyl sulfonate mimics the action of taurine on long-term synaptic potentiation. *Amino Acids* 2016, **48**(11)**:** 2647-2656.

21. Alghareeb SA, Alsughayyir J, Alfhili MA. Stimulation of Hemolysis and Eryptosis by α-Mangostin through Rac1 GTPase and Oxidative Injury in Human Red Blood Cells. *Molecules* 2023, **28**(18).

22. Sultan SA, Khawaji MH, Alsughayyir J, Alfhili MA, Alamri HS, Alrfaei BM. Antileukemic activity of sulfoxide nutraceutical allicin against THP-1 cells is associated with premature phosphatidylserine exposure in human erythrocytes. *Saudi J Biol Sci* 2020, **27**(12)**:** 3376-3384.

23. Harrison SA, Goodman Z, Jabbar A, Vemulapalli R, Younes ZH, Freilich B*, et al.* A randomized, placebo-controlled trial of emricasan in patients with NASH and F1-F3 fibrosis. *J Hepatol* 2020, **72**(5)**:** 816-827.

24. Gatidis S, Zelenak C, Fajol A, Lang E, Jilani K, Michael D*, et al.* p38 MAPK activation and function following osmotic shock of erythrocytes. *Cell Physiol Biochem* 2011, **28**(6)**:** 1279-1286.

25. Alghareeb SA, Alfhili MA, Alsughayyir J. Stimulation of Hemolysis and Eryptosis by β-Caryophyllene Oxide. *Life (Basel)* 2023, **13**(12).

26. Attanzio A, Frazzitta A, Cilla A, Livrea MA, Tesoriere L, Allegra M. 7-Keto-Cholesterol and Cholestan-3beta, 5alpha, 6beta-Triol Induce Eryptosis through Distinct Pathways Leading to NADPH Oxidase and Nitric Oxide Synthase Activation. *Cell Physiol Biochem* 2019, **53**(6)**:** 933-947.

27. Lang PA, Kempe DS, Myssina S, Tanneur V, Birka C, Laufer S*, et al.* PGE(2) in the regulation of programmed erythrocyte death. *Cell Death Differ* 2005, **12**(5)**:** 415-428.

28. Officioso A, Alzoubi K, Manna C, Lang F. Clofazimine Induced Suicidal Death of Human Erythrocytes. *Cell Physiol Biochem* 2015, **37**(1)**:** 331-341.

29. Han S, Ye X, Yang J, Peng X, Jiang X, Li J*, et al.* Host specific sphingomyelin is critical for replication of diverse RNA viruses. *Cell Chem Biol* 2024.

30. Shumilina E, Kiedaisch V, Akkel A, Lang P, Hermle T, Kempe DS*, et al.* Stimulation of suicidal erythrocyte death by lipoxygenase inhibitor Bay-Y5884. *Cell Physiol Biochem* 2006, **18**(4-5)**:** 233-242.

31. Bhavsar SK, Gu S, Bobbala D, Lang F. Janus kinase 3 is expressed in erythrocytes, phosphorylated upon energy depletion and involved in the regulation of suicidal erythrocyte death. *Cell Physiol Biochem* 2011, **27**(5)**:** 547-556.

32. Naqvi S, Macdonald A, McCoy CE, Darragh J, Reith AD, Arthur JS. Characterization of the cellular action of the MSK inhibitor SB-747651A. *Biochem J* 2012, **441**(1)**:** 347-357.

33. Föller M, Sopjani M, Koka S, Gu S, Mahmud H, Wang K*, et al.* Regulation of erythrocyte survival by AMP-activated protein kinase. *Faseb j* 2009, **23**(4)**:** 1072-1080.

34. Hawley SA, Ross FA, Russell FM, Atrih A, Lamont DJ, Hardie DG. Mechanism of Activation of AMPK by Cordycepin. *Cell Chem Biol* 2020, **27**(2)**:** 214-222.e214.

35. Rogacka D, Rachubik P, Audzeyenka I, Szrejder M, Kulesza T, Myślińska D*, et al.* Enhancement of cGMP-dependent pathway activity ameliorates hyperglycemia-induced decrease in SIRT1-AMPK activity in podocytes: Impact on glucose uptake and podocyte function. *Biochim Biophys Acta Mol Cell Res* 2022, **1869**(12)**:** 119362.

36. Wu R, Yao K, Flammer J, Haefliger IO. Role of anions in nitric oxide-induced short-circuit current increase in isolated porcine ciliary processes. *Invest Ophthalmol Vis Sci* 2004, **45**(9)**:** 3213-3222.

37. Liu Z, Liu Y, Qian L, Jiang S, Gai X, Ye S*, et al.* A proteomic and phosphoproteomic landscape of KRAS mutant cancers identifies combination therapies. *Mol Cell* 2021, **81**(19)**:** 4076-4090.e4078.

38. Fu C, Hu X, Wang S, Yu X, Zhang Q, Zhang L*, et al.* Inhibition of PAK1 generates an ameliorative effect on MPLW515L mouse model of myeloproliferative neoplasms by regulating the differentiation and survival of megakaryocytes. *Exp Hematol* 2023, **127:** 59-69.e52.

39. Feldman RI, Wu JM, Polokoff MA, Kochanny MJ, Dinter H, Zhu D*, et al.* Novel small molecule inhibitors of 3-phosphoinositide-dependent kinase-1. *J Biol Chem* 2005, **280**(20)**:** 19867-19874.

40. Lang E, Zelenak C, Eberhard M, Bissinger R, Rotte A, Ghashghaeinia M*, et al.* Impact of cyclin-dependent kinase CDK4 inhibition on eryptosis. *Cell Physiol Biochem* 2015, **37**(3)**:** 1178-1186.

41. Alfhili MA, Weidner DA, Lee MH. Disruption of erythrocyte membrane asymmetry by triclosan is preceded by calcium dysregulation and p38 MAPK and RIP1 stimulation. *Chemosphere* 2019, **229:** 103-111.

42. Park HH, Park SY, Mah S, Park JH, Hong SS, Hong S*, et al.* HS-1371, a novel kinase inhibitor of RIP3-mediated necroptosis. *Exp Mol Med* 2018, **50**(9)**:** 1-15.

43. Alharthy FH, Alsughayyir J, Alfhili MA. Linolenic acid stimulates eryptosis and hemolysis through oxidative stress and CK1α/MLKL: protective role of melatonin, urea, and polyethylene glycol. *Drug Chem Toxicol* 2024**:** 1-11.

44. McCaig WD, Hodges AL, Deragon MA, Haluska RJ, Jr., Bandyopadhyay S, Ratner AJ*, et al.* Storage Primes Erythrocytes for Necroptosis and Clearance. *Cell Physiol Biochem* 2019, **53**(3)**:** 496-507.

45. Mahmud H, Qadri SM, Föller M, Lang F. Inhibition of suicidal erythrocyte death by vitamin C. *Nutrition* 2010, **26**(6)**:** 671-676.

46. Jain SK. Vitamin E and stabilization of membrane lipid organization in red blood cells with peroxidative damage. *Biomed Biochim Acta* 1983, **42**(11-12)**:** S43-47.

47. Föller M, Harris IS, Elia A, John R, Lang F, Kavanagh TJ*, et al.* Functional significance of glutamate-cysteine ligase modifier for erythrocyte survival in vitro and in vivo. *Cell Death Differ* 2013, **20**(10)**:** 1350-1358.

48. Dumaswala UJ, Wilson MJ, Wu YL, Wykle J, Zhuo L, Douglass LM*, et al.* Glutathione loading prevents free radical injury in red blood cells after storage. *Free Radic Res* 2000, **33**(5)**:** 517-529.

49. Jain SK, Palmer M, Chen Y. Effect of vitamin E and N-acetylcysteine on phosphatidylserine externalization and induction of coagulation by high-glucose-treated human erythrocytes. *Metabolism* 1999, **48**(8)**:** 957-959.

50. Tesoriere L, D'Arpa D, Conti S, Giaccone V, Pintaudi AM, Livrea MA. Melatonin protects human red blood cells from oxidative hemolysis: new insights into the radical-scavenging activity. *J Pineal Res* 1999, **27**(2)**:** 95-105.

51. Klingelhoeffer C, Kämmerer U, Koospal M, Mühling B, Schneider M, Kapp M*, et al.* Natural resistance to ascorbic acid induced oxidative stress is mainly mediated by catalase activity in human cancer cells and catalase-silencing sensitizes to oxidative stress. *BMC Complement Altern Med* 2012, **12:** 61.

52. Nicolay JP, Liebig G, Niemoeller OM, Koka S, Ghashghaeinia M, Wieder T*, et al.* Inhibition of suicidal erythrocyte death by nitric oxide. *Pflugers Arch* 2008, **456**(2)**:** 293-305.

53. Yang HL, Korivi M, Lin MK, Chang HC, Wu CR, Lee MS*, et al.* Antihemolytic and antioxidant properties of pearl powder against 2,2'-azobis(2-amidinopropane) dihydrochloride-induced hemolysis and oxidative damage to erythrocyte membrane lipids and proteins. *J Food Drug Anal* 2017, **25**(4)**:** 898-907.

54. Bigdelou P, Farnoud AM. Induction of Eryptosis in Red Blood Cells Using a Calcium Ionophore. *J Vis Exp* 2020(155).

55. Qadri SM, Mahmud H, Föller M, Lang F. Inhibition of suicidal erythrocyte death by xanthohumol. *J Agric Food Chem* 2009, **57**(16)**:** 7591-7595.

56. Alfhili MA, Basudan AM, Alsughayyir J. Antiproliferative Wnt inhibitor wogonin prevents eryptosis following ionophoric challenge, hyperosmotic shock, oxidative stress, and metabolic deprivation. *J Food Biochem* 2021, **45**(11)**:** e13977.

57. Lang KS, Myssina S, Lang PA, Tanneur V, Kempe DS, Mack AF*, et al.* Inhibition of erythrocyte phosphatidylserine exposure by urea and Cl. *Am J Physiol Renal Physiol* 2004, **286**(6)**:** F1046-1053.

58. Han JH, Karki R, Malireddi RKS, Mall R, Sarkar R, Sharma BR*, et al.* NINJ1 mediates inflammatory cell death, PANoptosis, and lethality during infection conditions and heat stress. *Nat Commun* 2024, **15**(1)**:** 1739.

59. Ghashghaeinia M, Toulany M, Saki M, Bobbala D, Fehrenbacher B, Rupec R*, et al.* The NFĸB pathway inhibitors Bay 11-7082 and parthenolide induce programmed cell death in anucleated Erythrocytes. *Cell Physiol Biochem* 2011, **27**(1)**:** 45-54.

60. Jemaà M, Mokdad Gargouri R, Lang F. Polo-like kinase inhibitor BI2536 induces eryptosis. *Wien Med Wochenschr* 2023, **173**(5-6)**:** 152-157.

61. Zelenak C, Föller M, Velic A, Krug K, Qadri SM, Viollet B*, et al.* Proteome analysis of erythrocytes lacking AMP-activated protein kinase reveals a role of PAK2 kinase in eryptosis. *J Proteome Res* 2011, **10**(4)**:** 1690-1697.

62. Nguyen DB, Wagner-Britz L, Maia S, Steffen P, Wagner C, Kaestner L*, et al.* Regulation of phosphatidylserine exposure in red blood cells. *Cell Physiol Biochem* 2011, **28**(5)**:** 847-856.

63. Föller M, Sopjani M, Mahmud H, Lang F. Vanadate-induced suicidal erythrocyte death. *Kidney Blood Press Res* 2008, **31**(2)**:** 87-93.

64. Allegra M, Restivo I, Fucarino A, Pitruzzella A, Vasto S, Livrea MA*, et al.* Proeryptotic Activity of 4-Hydroxynonenal: A New Potential Physiopathological Role for Lipid Peroxidation Products. *Biomolecules* 2020, **10**(5).

65. Niemoeller OM, Mahmud H, Föller M, Wieder T, Lang F. Ciglitazone and 15d-PGJ2 induced suicidal erythrocyte death. *Cell Physiol Biochem* 2008, **22**(1-4)**:** 237-244.

66. Bhavsar SK, Bobbala D, Xuan NT, Föller M, Lang F. Stimulation of suicidal erythrocyte death by α-lipoic acid. *Cell Physiol Biochem* 2010, **26**(6)**:** 859-868.

67. Mischitelli M, Jemaàa M, Fezai M, Almasry M, Lang F, Faggio C. Stimulation of Erythrocyte Cell Membrane Scrambling by Adarotene. *Cell Physiol Biochem* 2017, **41**(2)**:** 519-529.

68. Nicolay JP, Gatz S, Liebig G, Gulbins E, Lang F. Amyloid induced suicidal erythrocyte death. *Cell Physiol Biochem* 2007, **19**(1-4)**:** 175-184.

69. Bentzen PJ, Lang F. Effect of anandamide on erythrocyte survival. *Cell Physiol Biochem* 2007, **20**(6)**:** 1033-1042.

70. Attanasio P, Shumilina E, Hermle T, Kiedaisch V, Lang PA, Huber SM*, et al.* Stimulation of eryptosis by anti-A IgG antibodies. *Cell Physiol Biochem* 2007, **20**(5)**:** 591-600.

71. Lang KS, Myssina S, Brand V, Sandu C, Lang PA, Berchtold S*, et al.* Involvement of ceramide in hyperosmotic shock-induced death of erythrocytes. *Cell Death Differ* 2004, **11**(2)**:** 231-243.

72. Kucherenko YV, Bhavsar SK, Grischenko VI, Fischer UR, Huber SM, Lang F. Increased cation conductance in human erythrocytes artificially aged by glycation. *J Membr Biol* 2010, **235**(3)**:** 177-189.

73. Gatidis S, Föller M, Lang F. Hemin-induced suicidal erythrocyte death. *Ann Hematol* 2009, **88**(8)**:** 721-726.

74. Foller M, Mahmud H, Gu S, Wang K, Floride E, Kucherenko Y*, et al.* Participation of leukotriene C(4) in the regulation of suicidal erythrocyte death. *J Physiol Pharmacol* 2009, **60**(3)**:** 135-143.

75. Wang K, Mahmud H, Föller M, Biswas R, Lang KS, Bohn E*, et al.* Lipopeptides in the triggering of erythrocyte cell membrane scrambling. *Cell Physiol Biochem* 2008, **22**(5-6)**:** 381-386.

76. Nicolay JP, Schneider J, Niemoeller OM, Artunc F, Portero-Otin M, Haik G, Jr.*, et al.* Stimulation of suicidal erythrocyte death by methylglyoxal. *Cell Physiol Biochem* 2006, **18**(4-5)**:** 223-232.

77. Frauenfeld L, Alzoubi K, Abed M, Lang F. Stimulation of erythrocyte cell membrane scrambling by mushroom tyrosinase. *Toxins (Basel)* 2014, **6**(3)**:** 1096-1108.

78. Tesoriere L, Attanzio A, Allegra M, Cilla A, Gentile C, Livrea MA. Oxysterol mixture in hypercholesterolemia-relevant proportion causes oxidative stress-dependent eryptosis. *Cell Physiol Biochem* 2014, **34**(4)**:** 1075-1089.

79. Lang PA, Kempe DS, Tanneur V, Eisele K, Klarl BA, Myssina S*, et al.* Stimulation of erythrocyte ceramide formation by platelet-activating factor. *J Cell Sci* 2005, **118**(Pt 6)**:** 1233-1243.

80. Abed M, Towhid ST, Pakladok T, Alesutan I, Götz F, Gulbins E*, et al.* Effect of bacterial peptidoglycan on erythrocyte death and adhesion to endothelial cells. *Int J Med Microbiol* 2013, **303**(4)**:** 182-189.

81. Föller M, Biswas R, Mahmud H, Akel A, Shumilina E, Wieder T*, et al.* Effect of peptidoglycans on erythrocyte survival. *Int J Med Microbiol* 2009, **299**(1)**:** 75-85.

82. Voelkl J, Alzoubi K, Mamar AK, Ahmed MS, Abed M, Lang F. Stimulation of suicidal erythrocyte death by increased extracellular phosphate concentrations. *Kidney Blood Press Res* 2013, **38**(1)**:** 42-51.

83. Noh JY, Lim KM, Bae ON, Chung SM, Lee SW, Joo KM*, et al.* Procoagulant and prothrombotic activation of human erythrocytes by phosphatidic acid. *Am J Physiol Heart Circ Physiol* 2010, **299**(2)**:** H347-355.

84. Niemoeller OM, Foller M, Lang C, Huber SM, Lang F. Retinoic acid induced suicidal erythrocyte death. *Cell Physiol Biochem* 2008, **21**(1-3)**:** 193-202.

85. Sopjani M, Föller M, Gulbins E, Lang F. Suicidal death of erythrocytes due to selenium-compounds. *Cell Physiol Biochem* 2008, **22**(5-6)**:** 387-394.

86. Abed M, Towhid ST, Mia S, Pakladok T, Alesutan I, Borst O*, et al.* Sphingomyelinase-induced adhesion of eryptotic erythrocytes to endothelial cells. *Am J Physiol Cell Physiol* 2012, **303**(9)**:** C991-999.

87. Qadri SM, Bauer J, Zelenak C, Mahmud H, Kucherenko Y, Lee SH*, et al.* Sphingosine but not sphingosine-1-phosphate stimulates suicidal erythrocyte death. *Cell Physiol Biochem* 2011, **28**(2)**:** 339-346.

88. Qadri SM, Eberhard M, Mahmud H, Föller M, Lang F. Stimulation of ceramide formation and suicidal erythrocyte death by vitamin K(3) (menadione). *Eur J Pharmacol* 2009, **623**(1-3)**:** 10-13.

89. Kiedaisch V, Akel A, Niemoeller OM, Wieder T, Lang F. Zinc-induced suicidal erythrocyte death. *Am J Clin Nutr* 2008, **87**(5)**:** 1530-1534.

90. Zbidah M, Lupescu A, Jilani K, Fajol A, Michael D, Qadri SM*, et al.* Apigenin-induced suicidal erythrocyte death. *J Agric Food Chem* 2012, **60**(1)**:** 533-538.

91. Malik A, Bissinger R, Calabrò S, Faggio C, Jilani K, Lang F. Aristolochic acid induced suicidal erythrocyte death. *Kidney Blood Press Res* 2014, **39**(5)**:** 408-419.

92. Gao M, Lau PM, Kong SK. Mitochondrial toxin betulinic acid induces in vitro eryptosis in human red blood cells through membrane permeabilization. *Arch Toxicol* 2014, **88**(3)**:** 755-768.

93. Lui JC, Wong JW, Suen YK, Kwok TT, Fung KP, Kong SK. Cordycepin induced eryptosis in mouse erythrocytes through a Ca2+-dependent pathway without caspase-3 activation. *Arch Toxicol* 2007, **81**(12)**:** 859-865.

94. Fink M, Al Mamun Bhuyan A, Zacharopoulou N, Lang F. Stimulation of Eryptosis, the Suicidal Erythrocyte Death, by Costunolide. *Cell Physiol Biochem* 2018, **50**(6)**:** 2283-2295.

95. Bissinger R, Lupescu A, Zelenak C, Jilani K, Lang F. Stimulation of eryptosis by cryptotanshinone. *Cell Physiol Biochem* 2014, **34**(2)**:** 432-442.

96. Bentzen PJ, Lang E, Lang F. Curcumin induced suicidal erythrocyte death. *Cell Physiol Biochem* 2007, **19**(1-4)**:** 153-164.

97. Abed M, Zoubi KA, Theurer M, Lang F. Effect of dermaseptin on erythrocytes. *Basic Clin Pharmacol Toxicol* 2013, **113**(5)**:** 347-352.

98. Mischitelli M, Jemaà M, Almasry M, Faggio C, Lang F. Triggering of Suicidal Erythrocyte Death by Fascaplysin. *Cell Physiol Biochem* 2016, **39**(4)**:** 1638-1647.

99. Gao M, Wong SY, Lau PM, Kong SK. Ferutinin induces in vitro eryptosis/erythroptosis in human erythrocytes through membrane permeabilization and calcium influx. *Chem Res Toxicol* 2013, **26**(8)**:** 1218-1228.

100. Lupescu A, Jilani K, Zelenak C, Zbidah M, Shaik N, Lang F. Induction of programmed erythrocyte death by gambogic acid. *Cell Physiol Biochem* 2012, **30**(2)**:** 428-438.

101. Lupescu A, Bissinger R, Warsi J, Jilani K, Lang F. Stimulation of erythrocyte cell membrane scrambling by gedunin. *Cell Physiol Biochem* 2014, **33**(6)**:** 1838-1848.

102. Zbidah M, Lupescu A, Shaik N, Lang F. Gossypol-induced suicidal erythrocyte death. *Toxicology* 2012, **302**(2-3)**:** 101-105.

103. Zbidah M, Lupescu A, Herrmann T, Yang W, Foller M, Jilani K*, et al.* Effect of honokiol on erythrocytes. *Toxicol In Vitro* 2013, **27**(6)**:** 1737-1745.

104. Liu J, Mamun Bhuyan AA, Ma K, Zhu X, Zhou K, Lang F. Myricetin-induced suicidal erythrocyte death. *Mol Biol Rep* 2023, **50**(5)**:** 4253-4260.

105. Noh JY, Park JS, Lim KM, Kim K, Bae ON, Chung SM*, et al.* A naphthoquinone derivative can induce anemia through phosphatidylserine exposure-mediated erythrophagocytosis. *J Pharmacol Exp Ther* 2010, **333**(2)**:** 414-420.

106. Jilani K, Qadri SM, Zelenak C, Lang F. Stimulation of suicidal erythrocyte death by oridonin. *Arch Biochem Biophys* 2011, **511**(1-2)**:** 14-20.

107. Ghashghaeinia M, Cluitmans JC, Toulany M, Saki M, Köberle M, Lang E*, et al.* Age sensitivity of NFκB abundance and programmed cell death in erythrocytes induced by NFκB inhibitors. *Cell Physiol Biochem* 2013, **32**(4)**:** 801-813.

108. Alzoubi K, Honisch S, Abed M, Lang F. Triggering of suicidal erythrocyte death by penta-O-galloyl-β-D-glucose. *Toxins (Basel)* 2013, **6**(1)**:** 54-65.

109. Bissinger R, Fischer S, Jilani K, Lang F. Stimulation of erythrocyte death by phloretin. *Cell Physiol Biochem* 2014, **34**(6)**:** 2256-2265.

110. Akiel M, Alsughayyir J, Basudan AM, Alamri HS, Dera A, Barhoumi T*, et al.* Physcion Induces Hemolysis and Premature Phosphatidylserine Externalization in Human Erythrocytes. *Biol Pharm Bull* 2021, **44**(3)**:** 372-378.

111. Eberhard M, Föller M, Lang F. Effect of phytic acid on suicidal erythrocyte death. *J Agric Food Chem* 2010, **58**(3)**:** 2028-2033.

112. Signoretto E, Castagna M, Lang F. Stimulation of Eryptosis, the Suicidal Erythrocyte Death by Piceatannol. *Cell Physiol Biochem* 2016, **38**(6)**:** 2300-2310.

113. Lupescu A, Jilani K, Zbidah M, Lang E, Lang F. Enhanced Ca2+ entry, ceramide formation, and apoptotic death of erythrocytes triggered by plumbagin. *J Nat Prod* 2012, **75**(11)**:** 1956-1961.

114. Gao M, Cheung KL, Lau IP, Yu WS, Fung KP, Yu B*, et al.* Polyphyllin D induces apoptosis in human erythrocytes through Ca²⁺ rise and membrane permeabilization. *Arch Toxicol* 2012, **86**(5)**:** 741-752.

115. Lupescu A, Jilani K, Zbidah M, Lang F. Induction of apoptotic erythrocyte death by rotenone. *Toxicology* 2012, **300**(3)**:** 132-137.

116. Lupescu A, Bissinger R, Jilani K, Lang F. In vitro induction of erythrocyte phosphatidylserine translocation by the natural naphthoquinone shikonin. *Toxins (Basel)* 2014, **6**(5)**:** 1559-1574.

117. Abed M, Herrmann T, Alzoubi K, Pakladok T, Lang F. Tannic acid induced suicidal erythrocyte death. *Cell Physiol Biochem* 2013, **32**(4)**:** 1106-1116.

118. Zelenak C, Pasham V, Jilani K, Tripodi PM, Rosaclerio L, Pathare G*, et al.* Tanshinone IIA stimulates erythrocyte phosphatidylserine exposure. *Cell Physiol Biochem* 2012, **30**(1)**:** 282-294.

119. Qadri SM, Mahmud H, Föller M, Lang F. Thymoquinone-induced suicidal erythrocyte death. *Food Chem Toxicol* 2009, **47**(7)**:** 1545-1549.

120. Theurer M, Shaik N, Lang F. Stimulation of suicidal erythrocyte death by trans-cinnamaldehyde. *Phytomedicine* 2013, **20**(12)**:** 1119-1123.

121. Jilani K, Abed M, Zelenak C, Lang E, Qadri SM, Lang F. Triggering of erythrocyte cell membrane scrambling by ursolic acid. *J Nat Prod* 2011, **74**(10)**:** 2181-2186.

122. Jilani K, Lupescu A, Zbidah M, Shaik N, Lang F. Withaferin A-stimulated Ca2+ entry, ceramide formation and suicidal death of erythrocytes. *Toxicol In Vitro* 2013, **27**(1)**:** 52-58.

123. Al Mamun Bhuyan A, Lang F. Stimulation of Eryptosis by Afatinib. *Cell Physiol Biochem* 2018, **47**(3)**:** 1259-1273.

124. Föller M, Geiger C, Mahmud H, Nicolay J, Lang F. Stimulation of suicidal erythrocyte death by amantadine. *Eur J Pharmacol* 2008, **581**(1-2)**:** 13-18.

125. Nicolay JP, Bentzen PJ, Ghashghaeinia M, Wieder T, Lang F. Stimulation of erythrocyte cell membrane scrambling by amiodarone. *Cell Physiol Biochem* 2007, **20**(6)**:** 1043-1050.

126. Mahmud H, Mauro D, Qadri SM, Föller M, Lang F. Triggering of suicidal erythrocyte death by amphotericin B. *Cell Physiol Biochem* 2009, **24**(3-4)**:** 263-270.

127. Alzoubi K, Calabrò S, Bissinger R, Abed M, Faggio C, Lang F. Stimulation of suicidal erythrocyte death by artesunate. *Cell Physiol Biochem* 2014, **34**(6)**:** 2232-2244.

128. Alzoubi K, Egler J, Abed M, Lang F. Enhanced eryptosis following auranofin exposure. *Cell Physiol Biochem* 2015, **37**(3)**:** 1018-1028.

129. Bobbala D, Koka S, Geiger C, Föller M, Huber SM, Lang F. Azathioprine favourably influences the course of malaria. *Malar J* 2009, **8:** 102.

130. Geiger C, Föller M, Herrlinger KR, Lang F. Azathioprine-induced suicidal erythrocyte death. *Inflamm Bowel Dis* 2008, **14**(8)**:** 1027-1032.

131. Lang E, Jilani K, Zelenak C, Pasham V, Bobbala D, Qadri SM*, et al.* Stimulation of suicidal erythrocyte death by benzethonium. *Cell Physiol Biochem* 2011, **28**(2)**:** 347-354.

132. Jilani K, Lang F. Carmustine-induced phosphatidylserine translocation in the erythrocyte membrane. *Toxins (Basel)* 2013, **5**(4)**:** 703-716.

133. Lupescu A, Bissinger R, Jilani K, Lang F. Triggering of suicidal erythrocyte death by celecoxib. *Toxins (Basel)* 2013, **5**(9)**:** 1543-1554.

134. Akel A, Hermle T, Niemoeller OM, Kempe DS, Lang PA, Attanasio P*, et al.* Stimulation of erythrocyte phosphatidylserine exposure by chlorpromazine. *Eur J Pharmacol* 2006, **532**(1-2)**:** 11-17.

135. Mahmud H, Föller M, Lang F. Suicidal erythrocyte death triggered by cisplatin. *Toxicology* 2008, **249**(1)**:** 40-44.

136. Niemoeller OM, Akel A, Lang PA, Attanasio P, Kempe DS, Hermle T*, et al.* Induction of eryptosis by cyclosporine. *Naunyn Schmiedebergs Arch Pharmacol* 2006, **374**(1)**:** 41-49.

137. Bobbala D, Koka S, Lang C, Boini KM, Huber SM, Lang F. Effect of cyclosporine on parasitemia and survival of Plasmodium berghei infected mice. *Biochem Biophys Res Commun* 2008, **376**(3)**:** 494-498.

138. Qadri SM, Kucherenko Y, Zelenak C, Jilani K, Lang E, Lang F. Dicoumarol activates Ca2+-permeable cation channels triggering erythrocyte cell membrane scrambling. *Cell Physiol Biochem* 2011, **28**(5)**:** 857-864.

139. Ghashghaeinia M, Bobbala D, Wieder T, Koka S, Brück J, Fehrenbacher B*, et al.* Targeting glutathione by dimethylfumarate protects against experimental malaria by enhancing erythrocyte cell membrane scrambling. *Am J Physiol Cell Physiol* 2010, **299**(4)**:** C791-804.

140. Bissinger R, Modicano P, Frauenfeld L, Lang E, Jacobi J, Faggio C*, et al.* Estramustine-induced suicidal erythrocyte death. *Cell Physiol Biochem* 2013, **32**(5)**:** 1426-1436.

141. Jilani K, Enkel S, Bissinger R, Almilaji A, Abed M, Lang F. Fluoxetine induced suicidal erythrocyte death. *Toxins (Basel)* 2013, **5**(7)**:** 1230-1243.

142. Eberhard M, Ferlinz K, Alizzi K, Cacciato PM, Faggio C, Föller M*, et al.* FTY720-induced suicidal erythrocyte death. *Cell Physiol Biochem* 2010, **26**(4-5)**:** 761-766.

143. Al Mamun Bhuyan A, Wagner T, Cao H, Lang F. Triggering of Suicidal Erythrocyte Death by Gefitinib. *Cell Physiol Biochem* 2017, **41**(4)**:** 1697-1708.

144. Jilani K, Qadri SM, Lang F. Geldanamycin-induced phosphatidylserine translocation in the erythrocyte membrane. *Cell Physiol Biochem* 2013, **32**(6)**:** 1600-1609.

145. Shaik N, Alhourani E, Bosc A, Liu G, Towhid S, Lupescu A*, et al.* Stimulation of suicidal erythrocyte death by ipratropium bromide. *Cell Physiol Biochem* 2012, **30**(6)**:** 1517-1525.

146. Nicolay JP, Gatz S, Lang F, Lang UE. Lithium-induced suicidal erythrocyte death. *J Psychopharmacol* 2010, **24**(10)**:** 1533-1539.

147. Alzoubi K, Alktifan B, Oswald G, Fezai M, Abed M, Lang F. Breakdown of phosphatidylserine asymmetry following treatment of erythrocytes with lumefantrine. *Toxins (Basel)* 2014, **6**(2)**:** 650-664.

148. Mahmud H, Föller M, Lang F. Stimulation of erythrocyte cell membrane scrambling by methyldopa. *Kidney Blood Press Res* 2008, **31**(5)**:** 299-306.

149. Munoz C, Alzoubi K, Jacobi J, Abed M, Lang F. Effect of miltefosine on erythrocytes. *Toxicol In Vitro* 2013, **27**(6)**:** 1913-1919.

150. Jacobi J, Lang E, Bissinger R, Frauenfeld L, Modicano P, Faggio C*, et al.* Stimulation of erythrocyte cell membrane scrambling by mitotane. *Cell Physiol Biochem* 2014, **33**(5)**:** 1516-1526.

151. Arnold M, Bissinger R, Lang F. Mitoxantrone-induced suicidal erythrocyte death. *Cell Physiol Biochem* 2014, **34**(5)**:** 1756-1767.

152. Ilyas S, Jilani K, Sikandar M, Siddiq S, Riaz M, Naveed A*, et al.* Stimulation of Erythrocyte Membrane Blebbing by Naproxen Sodium. *Dose Response* 2020, **18**(1)**:** 1559325819899259.

153. Arnold M, Lang E, Modicano P, Bissinger R, Faggio C, Abed M*, et al.* Effect of nitazoxanide on erythrocytes. *Basic Clin Pharmacol Toxicol* 2014, **114**(5)**:** 421-426.

154. Lupescu A, Bissinger R, Herrmann T, Oswald G, Jilani K, Lang F. Induction of suicidal erythrocyte death by novobiocin. *Cell Physiol Biochem* 2014, **33**(3)**:** 670-680.

155. Malik A, Bissinger R, Jilani K, Lang F. Stimulation of erythrocyte cell membrane scrambling by nystatin. *Basic Clin Pharmacol Toxicol* 2015, **116**(1)**:** 47-52.

156. Koka S, Bobbala D, Lang C, Boini KM, Huber SM, Lang F. Influence of paclitaxel on parasitemia and survival of Plasmodium berghei infected mice. *Cell Physiol Biochem* 2009, **23**(1-3)**:** 191-198.

157. Lang PA, Huober J, Bachmann C, Kempe DS, Sobiesiak M, Akel A*, et al.* Stimulation of erythrocyte phosphatidylserine exposure by paclitaxel. *Cell Physiol Biochem* 2006, **18**(1-3)**:** 151-164.

158. Signoretto E, Zierle J, Bissinger R, Castagna M, Bossi E, Lang F. Triggering of Suicidal Erythrocyte Death by Pazopanib. *Cell Physiol Biochem* 2016, **38**(3)**:** 926-938.

159. Zelenak C, Eberhard M, Jilani K, Qadri SM, Macek B, Lang F. Protein kinase CK1α regulates erythrocyte survival. *Cell Physiol Biochem* 2012, **29**(1-2)**:** 171-180.

160. Kucherenko Y, Zelenak C, Eberhard M, Qadri SM, Lang F. Effect of casein kinase 1α activator pyrvinium pamoate on erythrocyte ion channels. *Cell Physiol Biochem* 2012, **30**(2)**:** 407-417.

161. Oswald G, Alzoubi K, Abed M, Lang F. Stimulation of suicidal erythrocyte death by ribavirin. *Basic Clin Pharmacol Toxicol* 2014, **114**(4)**:** 311-317.

162. Abed M, Towhid ST, Shaik N, Lang F. Stimulation of suicidal death of erythrocytes by rifampicin. *Toxicology* 2012, **302**(2-3)**:** 123-128.

163. Bissinger R, Malik A, Jilani K, Lang F. Triggering of erythrocyte cell membrane scrambling by salinomycin. *Basic Clin Pharmacol Toxicol* 2014, **115**(5)**:** 396-402.

164. Al Mamun Bhuyan A, Nüßle S, Cao H, Zhang S, Lang F. Simvastatin, a Novel Stimulator of Eryptosis, the Suicidal Erythrocyte Death. *Cell Physiol Biochem* 2017, **43**(2)**:** 492-506.

165. Lupescu A, Shaik N, Jilani K, Zelenak C, Lang E, Pasham V*, et al.* Enhanced erythrocyte membrane exposure of phosphatidylserine following sorafenib treatment: an in vivo and in vitro study. *Cell Physiol Biochem* 2012, **30**(4)**:** 876-888.

166. Zbidah M, Lupescu A, Yang W, Bosc A, Jilani K, Shaik N*, et al.* Sulindac sulfide--induced stimulation of eryptosis. *Cell Physiol Biochem* 2012, **30**(4)**:** 1072-1082.

167. Shaik N, Lupescu A, Lang F. Sunitinib-sensitive suicidal erythrocyte death. *Cell Physiol Biochem* 2012, **30**(3)**:** 512-522.

168. Fink M, Al Mamun Bhuyan A, Zacharopoulou N, Lang F. Taurolidine Sensitivity of Eryptosis, the Suicidal Erythrocyte Death. *Cell Physiol Biochem* 2018, **51**(2)**:** 501-512.

169. Lang E, Modicano P, Arnold M, Bissinger R, Faggio C, Abed M*, et al.* Effect of thioridazine on erythrocytes. *Toxins (Basel)* 2013, **5**(10)**:** 1918-1931.

170. Bissinger R, Bouguerra G, Stockinger K, Abbès S, Lang F. Triggering of Suicidal Erythrocyte Death by Topotecan. *Cell Physiol Biochem* 2015, **37**(4)**:** 1607-1618.

171. Qadri SM, Kucherenko Y, Lang F. Beauvericin induced erythrocyte cell membrane scrambling. *Toxicology* 2011, **283**(1)**:** 24-31.

172. Jilani K, Qadri SM, Lang E, Zelenak C, Rotte A, Bobbala D*, et al.* Stimulation of erythrocyte phospholipid scrambling by enniatin A. *Mol Nutr Food Res* 2011, **55 Suppl 2:** S294-302.

173. Zbidah M, Lupescu A, Jilani K, Lang F. Stimulation of suicidal erythrocyte death by fumagillin. *Basic Clin Pharmacol Toxicol* 2013, **112**(5)**:** 346-351.

174. Lang PA, Kaiser S, Myssina S, Birka C, Weinstock C, Northoff H*, et al.* Effect of Vibrio parahaemolyticus haemolysin on human erythrocytes. *Cell Microbiol* 2004, **6**(4)**:** 391-400.

175. Ahmed MS, Abed M, Voelkl J, Lang F. Triggering of suicidal erythrocyte death by uremic toxin indoxyl sulfate. *BMC Nephrol* 2013, **14:** 244.

176. Föller M, Shumilina E, Lam R, Mohamed W, Kasinathan R, Huber S*, et al.* Induction of suicidal erythrocyte death by listeriolysin from Listeria monocytogenes. *Cell Physiol Biochem* 2007, **20**(6)**:** 1051-1060.

177. Bhavsar SK, Eberhard M, Bobbala D, Lang F. Monensin induced suicidal erythrocyte death. *Cell Physiol Biochem* 2010, **25**(6)**:** 745-752.

178. Bouguerra G, Bissinger R, Abbès S, Lang F. Stimulation of Eryptosis by Narasin. *Cell Physiol Biochem* 2015, **37**(5)**:** 1807-1816.

179. Jilani K, Lupescu A, Zbidah M, Abed M, Shaik N, Lang F. Enhanced apoptotic death of erythrocytes induced by the mycotoxin ochratoxin A. *Kidney Blood Press Res* 2012, **36**(1)**:** 107-118.

180. Lupescu A, Jilani K, Zbidah M, Lang F. Patulin-induced suicidal erythrocyte death. *Cell Physiol Biochem* 2013, **32**(2)**:** 291-299.

181. Schneider J, Nicolay JP, Foller M, Wieder T, Lang F. Suicidal erythrocyte death following cellular K+ loss. *Cell Physiol Biochem* 2007, **20**(1-4)**:** 35-44.

182. Jilani K, Lang F. Ca(2+)-dependent suicidal erythrocyte death following zearalenone exposure. *Arch Toxicol* 2013, **87**(10)**:** 1821-1828.

183. Ahmed MS, Langer H, Abed M, Voelkl J, Lang F. The uremic toxin acrolein promotes suicidal erythrocyte death. *Kidney Blood Press Res* 2013, **37**(2-3)**:** 158-167.

184. Vota DM, Crisp RL, Nesse AB, Vittori DC. Oxidative stress due to aluminum exposure induces eryptosis which is prevented by erythropoietin. *J Cell Biochem* 2012, **113**(5)**:** 1581-1589.

185. Mahmud H, Föller M, Lang F. Arsenic-induced suicidal erythrocyte death. *Arch Toxicol* 2009, **83**(2)**:** 107-113.

186. Braun M, Föller M, Gulbins E, Lang F. Eryptosis triggered by bismuth. *Biometals* 2009, **22**(3)**:** 453-460.

187. Officioso A, Manna C, Alzoubi K, Lang F. Bromfenvinphos induced suicidal death of human erythrocytes. *Pestic Biochem Physiol* 2016, **126:** 58-63.

188. Sopjani M, Föller M, Dreischer P, Lang F. Stimulation of eryptosis by cadmium ions. *Cell Physiol Biochem* 2008, **22**(1-4)**:** 245-252.

189. Lang E, Qadri SM, Jilani K, Zelenak C, Lupescu A, Schleicher E*, et al.* Carbon monoxide-sensitive apoptotic death of erythrocytes. *Basic Clin Pharmacol Toxicol* 2012, **111**(5)**:** 348-355.

190. Lupescu A, Jilani K, Zelenak C, Zbidah M, Qadri SM, Lang F. Hexavalent chromium-induced erythrocyte membrane phospholipid asymmetry. *Biometals* 2012, **25**(2)**:** 309-318.

191. Restivo I, Attanzio A, Giardina IC, Di Gaudio F, Tesoriere L, Allegra M. Cigarette Smoke Extract Induces p38 MAPK-Initiated, Fas-Mediated Eryptosis. *International Journal of Molecular Sciences* 2022, **23**(23)**:** 14730.

192. Lang PA, Schenck M, Nicolay JP, Becker JU, Kempe DS, Lupescu A*, et al.* Liver cell death and anemia in Wilson disease involve acid sphingomyelinase and ceramide. *Nat Med* 2007, **13**(2)**:** 164-170.

193. Sopjani M, Föller M, Lang F. Gold stimulates Ca2+ entry into and subsequent suicidal death of erythrocytes. *Toxicology* 2008, **244**(2-3)**:** 271-279.

194. Zhang R, Xiang Y, Ran Q, Deng X, Xiao Y, Xiang L*, et al.* Involvement of calcium, reactive oxygen species, and ATP in hexavalent chromium-induced damage in red blood cells. *Cell Physiol Biochem* 2014, **34**(5)**:** 1780-1791.

195. Eisele K, Lang PA, Kempe DS, Klarl BA, Niemöller O, Wieder T*, et al.* Stimulation of erythrocyte phosphatidylserine exposure by mercury ions. *Toxicol Appl Pharmacol* 2006, **210**(1-2)**:** 116-122.

196. Alfhili MA, Alamri HS, Alsughayyir J, Basudan AM. Induction of hemolysis and eryptosis by occupational pollutant nickel chloride is mediated through calcium influx and p38 MAP kinase signaling. *Int J Occup Med Environ Health* 2022, **35**(1)**:** 1-11.

197. Föller M, Sopjani M, Schlemmer HP, Claussen CD, Lang F. Triggering of suicidal erythrocyte death by radiocontrast agents. *Eur J Clin Invest* 2009, **39**(7)**:** 576-583.

198. Sopjani M, Föller M, Haendeler J, Götz F, Lang F. Silver ion-induced suicidal erythrocyte death. *J Appl Toxicol* 2009, **29**(6)**:** 531-536.

199. Nguyen TT, Föller M, Lang F. Tin triggers suicidal death of erythrocytes. *J Appl Toxicol* 2009, **29**(1)**:** 79-83.

200. Niemoeller OM, Bentzen PJ, Lang E, Lang F. Adenosine protects against suicidal erythrocyte death. *Pflugers Arch* 2007, **454**(3)**:** 427-439.

201. Delveaux J, Turpin C, Veeren B, Diotel N, Bravo SB, Begue F*, et al.* Antirhea borbonica Aqueous Extract Protects Albumin and Erythrocytes from Glycoxidative Damages. *Antioxidants (Basel)* 2020, **9**(5).

202. Ghashghaeinia M, Koralkova P, Giustarini D, Mojzikova R, Fehrenbacher B, Dreischer P*, et al.* The specific PKC-α inhibitor chelerythrine blunts costunolide-induced eryptosis. *Apoptosis* 2020, **25**(9-10)**:** 674-685.

203. Singh S, Garg G, Singh AK, Bissoyi A, Rizvi SI. Fisetin, a potential caloric restriction mimetic, attenuates senescence biomarkers in rat erythrocytes. *Biochem Cell Biol* 2019, **97**(4)**:** 480-487.

204. Tortora F, Notariale R, Lang F, Manna C. Hydroxytyrosol Decreases Phosphatidylserine Exposure and Inhibits Suicidal Death Induced by Lysophosphatidic Acid in Human Erythrocytes. *Cell Physiol Biochem* 2019, **53**(6)**:** 921-932.

205. Officioso A, Alzoubi K, Lang F, Manna C. Hydroxytyrosol inhibits phosphatidylserine exposure and suicidal death induced by mercury in human erythrocytes: Possible involvement of the glutathione pathway. *Food Chem Toxicol* 2016, **89:** 47-53.

206. Tesoriere L, Attanzio A, Allegra M, Livrea MA. Dietary indicaxanthin from cactus pear (Opuntia ficus-indica L. Mill) fruit prevents eryptosis induced by oxysterols in a hypercholesterolaemia-relevant proportion and adhesion of human erythrocytes to endothelial cell layers. *Br J Nutr* 2015, **114**(3)**:** 368-375.

207. Restivo I, Giardina IC, Barone R, Cilla A, Burgio S, Allegra M*, et al.* Indicaxanthin prevents eryptosis induced by cigarette smoke extract by interfering with active Fas-mediated signaling. *Biofactors* 2024.

208. Lang PA, Kempe DS, Akel A, Klarl BA, Eisele K, Podolski M*, et al.* Inhibition of erythrocyte "apoptosis" by catecholamines. *Naunyn Schmiedebergs Arch Pharmacol* 2005, **372**(3)**:** 228-235.

209. Sun Y, Liu G, Li X, Shi Y, Guan G. L-Carnitine inhibits eryptosis induced by uremic serum and the related mechanisms. *Ren Fail* 2015, **37**(6)**:** 1050-1056.

210. Shaik N, Zbidah M, Lang F. Inhibition of Ca(2+) entry and suicidal erythrocyte death by naringin. *Cell Physiol Biochem* 2012, **30**(3)**:** 678-686.

211. Gatidis S, Meier A, Jilani K, Lang E, Zelenak C, Qadri SM*, et al.* Phlorhizin protects against erythrocyte cell membrane scrambling. *J Agric Food Chem* 2011, **59**(15)**:** 8524-8530.

212. Alvarez-Sala A, López-García G, Attanzio A, Tesoriere L, Cilla A, Barberá R*, et al.* Effects of Plant Sterols or β-Cryptoxanthin at Physiological Serum Concentrations on Suicidal Erythrocyte Death. *J Agric Food Chem* 2018, **66**(5)**:** 1157-1166.

213. Restivo I, Attanzio A, Tesoriere L, Allegra M, Garcia-Llatas G, Cilla A. A Mixture of Dietary Plant Sterols at Nutritional Relevant Serum Concentration Inhibits Extrinsic Pathway of Eryptosis Induced by Cigarette Smoke Extract. *Int J Mol Sci* 2023, **24**(2).

214. Liu J, Bhuyan AAM, Ma K, Zhang S, Cheng A, Lang F. Inhibition of suicidal erythrocyte death by pyrogallol. *Mol Biol Rep* 2020, **47**(7)**:** 5025-5032.

215. Qadri SM, Föller M, Lang F. Inhibition of suicidal erythrocyte death by resveratrol. *Life Sci* 2009, **85**(1-2)**:** 33-38.

216. Farag MR, Alagawany M, Tufarelli V. In vitro antioxidant activities of resveratrol, cinnamaldehyde and their synergistic effect against cyadox-induced cytotoxicity in rabbit erythrocytes. *Drug Chem Toxicol* 2017, **40**(2)**:** 196-205.

217. Qian EW, Ge DT, Kong SK. Salidroside protects human erythrocytes against hydrogen peroxide-induced apoptosis. *J Nat Prod* 2012, **75**(4)**:** 531-537.

218. Klarl BA, Lang PA, Kempe DS, Niemoeller OM, Akel A, Sobiesiak M*, et al.* Protein kinase C mediates erythrocyte "programmed cell death" following glucose depletion. *Am J Physiol Cell Physiol* 2006, **290**(1)**:** C244-253.

219. Kengaiah J, Nandish SKM, Ramachandraiah C, Chandramma, Shivaiah A, Vishalakshi GJ*, et al.* Protective Effect of Tamarind Seed Coat Ethanol Extract on Eryptosis Induced by Oxidative Stress. *Biochemistry (Mosc)* 2020, **85**(1)**:** 119-129.

220. Mahmud H, Mauro D, Foller M, Lang F. Inhibitory effect of thymol on suicidal erythrocyte death. *Cell Physiol Biochem* 2009, **24**(5-6)**:** 407-414.

221. Shan F, Yang R, Ji T, Jiao F. Vitamin C Inhibits Aggravated Eryptosis by Hydrogen Peroxide in Glucose-6-Phosphated Dehydrogenase Deficiency. *Cell Physiol Biochem* 2016, **39**(4)**:** 1453-1462.

222. Brand V, Koka S, Lang C, Jendrossek V, Huber SM, Gulbins E*, et al.* Influence of amitriptyline on eryptosis, parasitemia and survival of Plasmodium berghei-infected mice. *Cell Physiol Biochem* 2008, **22**(5-6)**:** 405-412.

223. Kucherenko YV, Lang F. Inhibitory effect of furosemide on non-selective voltage-independent cation channels in human erythrocytes. *Cell Physiol Biochem* 2012, **30**(4)**:** 863-875.

224. Kucherenko Y, Geiger C, Shumilina E, Föller M, Lang F. Inhibition of cation channels and suicidal death of human erythrocytes by zidovudine. *Toxicology* 2008, **253**(1-3)**:** 62-69.
